# Methods for analyzing recurrent and terminal events considering composite and competing endpoints in clinical trials: a practical overview

**DOI:** 10.1186/s12874-026-03008-7

**Published:** 2026-09-12

**Authors:** Alidan Duoerkongjiang, Antonia Zapf, Paulus Kirchhof, Ann-Kathrin Ozga

**Affiliations:** 1 Institute of Medical Biometry and Epidemiology, University Medical Center Hamburg-Eppendorf, Hamburg, Germany; 2 Department of Cardiology, University Heart and Vascular Center Hamburg, University Medical Center Hamburg-Eppendorf, Hamburg, Germany

**Keywords:** Time to event, Multiple events, Overview, Method comparison, Tutorial in R

## Abstract

Time to event endpoints like time to myocardial infarction, relapse, or death are often of primary interest in clinical trials. To describe the whole disease process and gain a complete picture of treatment effects, methods for recurrent time to event analysis considering terminal events were proposed, i.e. methods considering all events that occur per individual. However, these methods are not often applied in clinical studies. This might be due to the researchers’ uncertainties about underlying model assumptions and interpretation of results. We therefore aim to give a comprehensive overview of methods for the analysis of multiple time to events comparing model assumptions and interpretation. We illustrate the methods using a cardiovascular trial and provide the corresponding R code.

**Supplementary Information:**

The online version contains supplementary material available at https://doi.org/10.1186/s12874-026-03008-7.

## Background

In clinical trials, the analysis of time to event endpoints such as time to disease recurrence, hospitalization, myocardial infarction, or death is of interest for evaluating treatment efficacy and patient outcomes. Thereby, mostly the time to the first occurring event for a patient is considered [1, 2]. However, to evaluate the efficacy of a treatment for the whole disease process, it might be attractive to include all information available for a patient, i.e. all events experienced by a patient like recurrent events or different event types. Traditional time to first event analysis methods like the Cox proportional hazards model [3] or Kaplan-Meier estimate [4] ignore subsequent events and fail to utilize the additional information provided by recurrent events. Furthermore, they typically do not differentiate between the clinical relevance of different event types, treating fatal and non-fatal events as equally relevant, which may not accurately reflect their true impact on patients. The common time to first event methods also neglect that there might be competing events, i.e. different types of events vie for occurrence, but only the first event is observed, introducing potential bias in estimation. Additionally, the inter-relationship between events can impact their likelihood or timing, necessitating more sophisticated analytical approaches. For instance, a myocardial infarction might influence the risk of subsequent events, such as another myocardial infarction, heart failure or death, indicating that events can be both competing and correlated. To address these challenges, alternative approaches for analyzing recurrent events have been explored by Andersen and Gill [5] or Prentice et al. [6]. More recently, Ozga et al. [7] have evaluated these methods through simulation studies in the context of composite endpoints that include both non fatal recurrent events and fatal events, allowing for the consideration of clinical relevance [8–12]. However, their investigation primarily focused on settings with only one type of non fatal recurrent event and one fatal event. It remains unanswered how to appropriately model more complex scenarios involving multiple different types of correlated event processes and underlying patient heterogeneity. Other approaches that are mentioned here for the analysis of such more complex settings with multiple events per patient include joint frailty methods [13, 14], the win ratio [15], or multi-state approaches [16]. However, these methods are not widely used in practice [17]. This may partly reflect limited awareness among applied researchers and uncertainty about the advantages and limitations of the different methods, what they analyze, how their results should be interpreted, and how they address the complexities of multiple time-to-event endpoints. Hence, it is of interest to evaluate which methods have the potential to be extended to capture more features. In subsequent work, it is furthermore of interest to compare these methods systematically via a simulation study. To bridge the gap between these complex methodologies and applied research, here we aim to give an overview of methods, their estimation, underlying assumptions, and interpretation. The contribution of this paper therefore lies not in the development of a new statistical method, but in providing an integrated, practice-oriented synthesis that links scientific questions, estimands, outcome structures, assumptions, interpretation, software availability, and clinical applicability within one decision-guided framework. We focus on randomized clinical trials. The methods are further illustrated with a real data example. Corresponding R Code [18] is given. The paper is structured as follows: We start by describing the data situation. Afterwards we subsequently describe the analysis methods. The methods are applied to a real data example. We end with some concluding remarks.

## Setting of interest

**Fig. 1:**
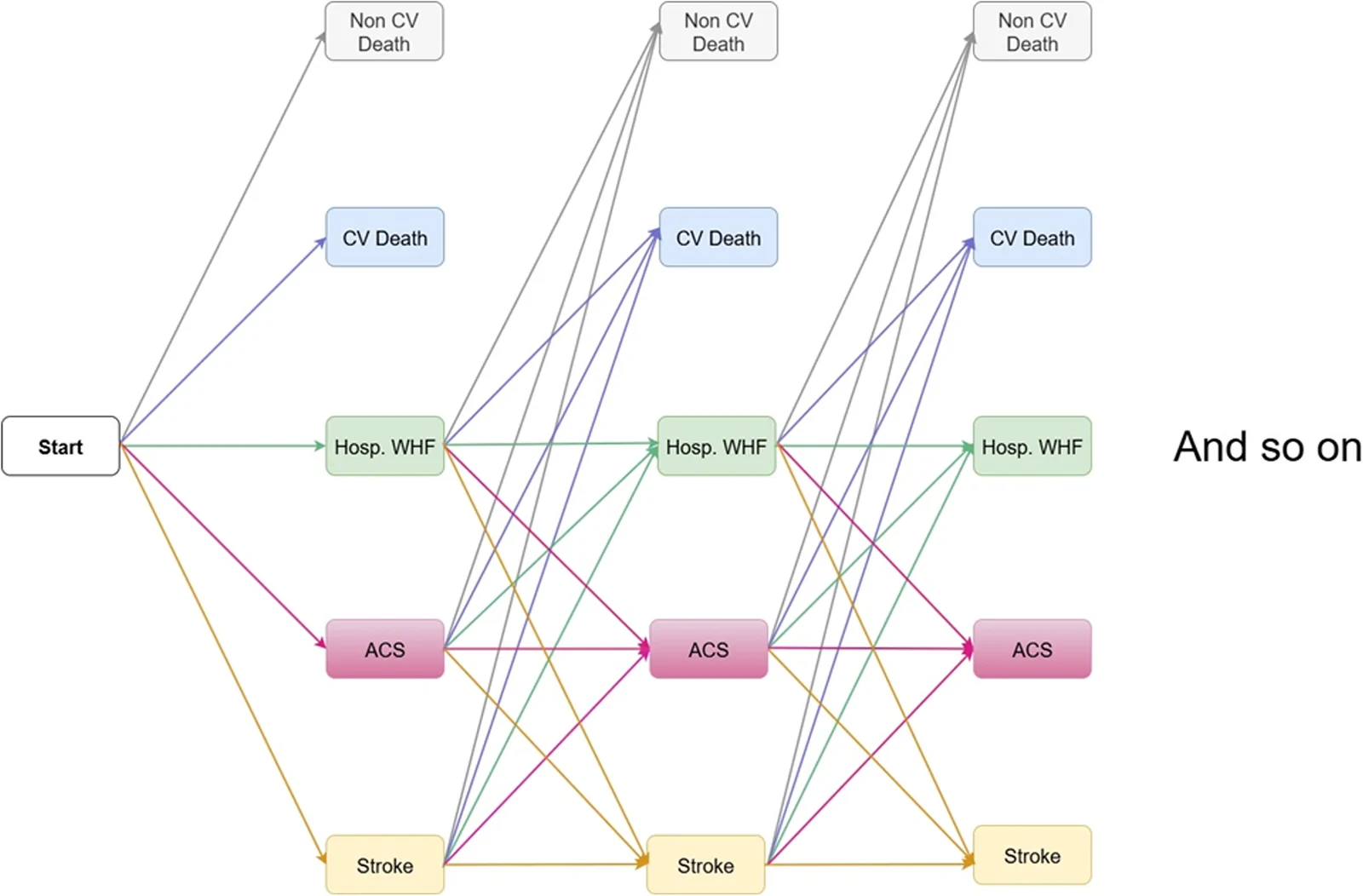
Setting of interest: CV = cardiovascular; Hosp. = hospitalization; WHF = worsening heart failure; ACS = acute coronary syndrome

The primary aim of this paper is to give an overview of methods for the analysis of multiple time to event endpoints in randomized clinical trials. Endpoints could correspond to different event types. E.g., in cardiovascular trials, one could observe non-cardiovascular death (non-CV death), cardiovascular death (CV death), hospitalization for worsening heart failure (Hosp. WHF), acute coronary syndrome (ACS), and/or stroke [1, 2]. Figure 1 illustrates the scenario of interest, taking into account all events that might occur per patient and not only the first.

Other example settings could be recurrent seizures in epileptic patients, multiple sclerosis recurrence, or tumor recurrences in cancer patients. All events that occur per patient shall be included in the analysis and not, as commonly done, only the first occurring event.

In this work, we focus on a randomized controlled trial with two groups (intervention vs. control); i.e. we are only interested in comparing the two groups and will not adjust for other variables.

## Methods

**Fig. 2:**
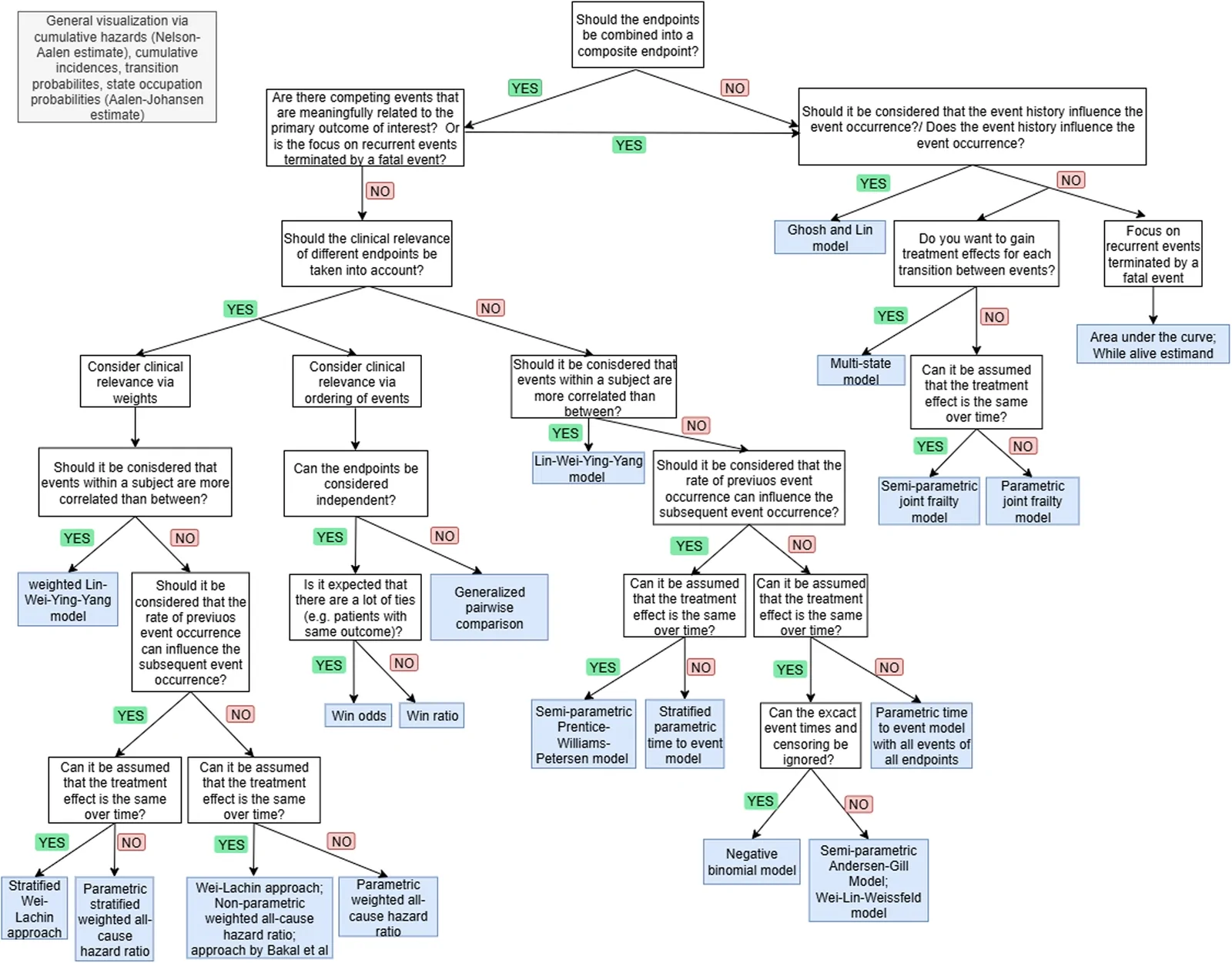
Selection criteria for different statistical approaches

In the following, we present a structured, decision-guided framework (illustrated in Fig. 2) for selecting appropriate statistical methods to analyze time-to-event data with recurrent, competing, and terminal events. The framework is intended as a practical navigation aid, not a ranking of interchangeable methods estimating one common treatment effect. Methods in different branches may target different estimands, such as marginal rate or intensity summaries, conditional or sequential effects, latent-variable model parameters, prioritized pairwise comparisons, or state-transition summaries. Therefore, the choice of method should be guided first by the scientific question and the target estimand. In the following method descriptions, data structure refers to the conceptual outcome structure required by each method; implementation-level data structures are provided with the R code in Additional file 2 (Appendix 2). The framework reflects clinical and statistical decision points commonly encountered in the design and analysis of clinical trials. In the following section, we first discuss methods for composite endpoints that do not consider clinical relevance. Subsequently, we present approaches that account for it, either by assigning weights or by using ordered endpoints. Finally, we review methods for composite endpoints considering correlated competing events, and alternative approaches using no composite endpoints. Key aspects of these methods and software availability are summarized in Table 1a (parts 1–4), Table 1b, and c. Detailed aspects, including model specification, estimation, interpretation, and underlying assumptions, are provided in Additional File 1 (Appendix 1) for conciseness.

**Table 1:** Summary of methods for composite endpoints: clinical relevance not considered

Category		Method	Short description	Model assumptions	Software	Suitable application
Clinical relevance not considered	Time-to-event methods	Andersen-Gill (AG) model [5, 7]	Semi-parametric; Cox model-based; one overall hazard ratio (HR).	Proportional hazards (PH) assumption; Markov assumption; same clinical relevance for endpoints.	R coxph [19]; STATA [20]; SAS [21].	Best suited when a composite endpoint is of interest, clinical relevance and event order can be neglected.
		Lin-Wei-Ying-Yang (LWYY) model [19, 22]	Semi-parametric rate model; one overall rate ratio (RR).	Ratio of mean cumulative count curves constant across time; same clinical relevance for endpoints.	R coxph [19]; STATA [20]; SAS [21].	Best suited when a composite endpoint is of interest, clinical relevance and event order can be neglected; preferable to AG when patient heterogeneity in event processes should be considered.
		Parametric time-to-event model with all events of all endpoints [23]	Parametric accelerated failure time (AFT) model; one overall acceleration factor as treatment effect.	Specific parametric event time distribution assumed; same clinical relevance for endpoints.	R flexsurvreg [24]; STATA [20]; SAS [21].	May be useful when a composite endpoint is of interest and the PH assumption of the AG model is not fulfilled.
		Prentice-Williams-Peterson (PWP) model [6, 7]	Semi-parametric; stratified Cox model-based; subsequent event occurs if the previous event was observed; one overall HR or strata-specific HRs.	Strata-specific PH assumption; less restrictive Markov assumption; same clinical relevance for endpoints.	R coxph [19]; STATA [20]; SAS [21].	Best suited when a composite endpoint is of interest, clinical relevance can be neglected but event order should be considered.
		Stratified parametric time-to-event model [23, 25]	Stratified parametric AFT model; one overall or stratified acceleration factor as treatment effect; subsequent event occurs if the previous event was observed.	Specific parametric distribution; same clinical relevance for endpoints.	R flexsurvreg [24]; STATA [20]; SAS [21].	Best suited when a composite endpoint is of interest; clinical relevance can be neglected, but event order and non-PH should be considered.
		Wei-Lin-Weissfeld (WLW) model [7, 26]	Semi-parametric; stratified Cox model-based; subsequent event can occur although no previous event was observed; one overall HR or strata-specific HRs.	Strata-specific PH assumption; Markov assumption; same clinical relevance for endpoints; subsequent event can occur although the previous event was not observed.	R survival::coxph [19]; STATA [20]; SAS [21].	Less suitable when a clearly interpretable treatment effect is required, due to unrealistic assumptions and difficult HR interpretation.
	Event count methods	Negative binomial (NB) model [27, 28]	Parametric distribution for event counts; incidence rate ratio (IRR).	Independence of individual observations; constant patient-specific incidence rate across time; same clinical relevance for endpoints.	R MASS::glm.nb [29]; STATA [20]; SAS [21].	Less suitable when event timing or censoring is important, because the method focuses on event counts.
Clinical relevance considered	Weighted methods	Weighted LWYY model [30]	Semi-parametric rate model; one overall weighted RR.	See LWYY, but clinical relevance is considered.	R Wcompo::CompoML [31].	Best suited when clinical relevance should be considered within a composite endpoint, event order can be neglected, and patient heterogeneity should be considered.
		Parametric weighted all-cause hazard ratio [8]	One overall weighted HR; weights work on cause-specific parametric hazards; non-negative weights.	Specific parametric event time distribution.	Not readily available yet.	Best suited when clinical relevance within a composite endpoint and non-PH should be considered, event order can be neglected.
		Parametric stratified weighted all-cause hazard ratio [8, 10]	One overall weighted HR; weights work on cause-specific parametric stratified hazards; non-negative weights.	Specific parametric event time distribution.	Not readily available yet.	Best suited when clinical relevance within a composite endpoint, non-PH and event order should be considered.
		Non-parametric weighted all-cause hazard ratio [8–10]	One overall weighted HR; weights work on cause-specific non-parametric cumulative hazards; non-negative weights.	Equal cause-specific baseline hazards.	Not readily available yet.	Best suited when clinical relevance within a composite endpoint should be considered and a time-constant treatment effect can be assumed.
		Wei-Lachin approach [10–12]	Semi-parametric; Cox model-based; one overall weighted HR; weights work on coefficients; weights sum to 1.	See AG, but clinical relevance is considered.	Not readily available yet.	Best suited when clinical relevance within a composite endpoint should be considered and a time-constant treatment effect can be assumed.
		Stratified Wei-Lachin approach [10]	Semi-parametric; stratified Cox model-based; one overall weighted HR; weights work on coefficients; weights sum to 1.	See PWP, but clinical relevance is considered.	Not readily available yet.	Best suited when clinical relevance within a composite endpoint, event order should be considered and a time-constant treatment effect can be assumed.
		Approach by Bakal [10, 32, 33]	Non-parametric; weighted Kaplan-Meier; weighted log-rank; no underlying model.	No risk change for subsequent events; patients remain at risk while under follow-up.	Not readily available yet.	Not suitable because no underlying model is defined, i.e. it is not clear what is estimated.
	Ordered endpoints	Win ratio/win odds [15, 34]	Non-parametric; ordered independent endpoints; one overall ratio.	Net effect; endpoints are independent.	R WINS::win.stat [35]; STATA [20].	Best suited when clinical relevance should be considered through endpoint prioritization for a composite endpoint. Endpoints have to be independent and clearly defined, i.e. how to handle recurrent events.
		Generalized pairwise comparisons of prioritized outcomes [36, 37]	Non-parametric; ordered endpoints can be dependent; net chance of a better outcome.	Net effect.	R BuyseTest [38].	Best suited when clinical relevance should be considered through prioritized outcomes within a composite endpoint. Endpoints have to be clearly defined and not independent, i.e. how to handle recurrent events.
Gain several treatment effects for different endpoints		Semi-parametric joint frailty model [13, 14, 39]	Cox model-based; two or more correlated hazards; two or more hazard ratios (HRs).	Proportional hazards (PH) and Markov assumption conditional on frailties; same clinical relevance of endpoints within the composite endpoint.	R frailtypack::frailtyPenal [40]; STATA [20].	Best suited when correlated event processes should be modeled and PH can be assumed.
		Parametric joint frailty model [14, 39]	Two or more correlated parametric hazards; two or more HRs.	Specific parametric event time distribution; same clinical relevance of endpoints within the composite endpoint.	R frailtypack::frailtyPenal [40]; STATA [20].	Best suited when correlated event processes should be modeled and non-PH should be considered.
		Multi-state model [41, 42]	Often semi-parametric, for example Cox-based; HRs for transitions between multiple states over time.	PH and Markov assumption in most cases.	R survival package [19]; STATA [20]; SAS [21].	May be useful for exploratory description of transitions between event states.
		Ghosh and Lin model [43]	Semi-parametric; marginal rate model; two rate ratios.	Proportional means/rates assumption for recurrent events; same clinical relevance of endpoints within the composite endpoint.	R reReg::reReg [44]; STATA [20]; SAS [21].	May be useful when the Markov assumption of the Andersen-Gill model is not fulfilled.
Gain one treatment effect		Area under the curve [45]	Non-parametric; cumulative mean events; one overall ratio or difference at a fixed time point.	Same clinical relevance for endpoints within the composite.	R MCC::CompareAUCs [46].	Best suited when the treatment effect on non-fatal event burden over time is of interest.
		While alive estimate [47, 48]	Non-parametric; expected number of recurrent events per unit of survival time; rate ratio of recurrent events while alive.	Same clinical relevance for endpoints within the composite.	R WA::LRfit [49].	Best suited when the treatment effect on non-fatal event burden among those alive is of interest.
General visualization/Data description	Cumulative hazards	Nelson-Aalen estimate [41, 42]	Non-parametric; describes cumulative cause-specific or transition hazards.	Markov assumption; same clinical relevance for endpoints within the composite.	R mvna::mvna [50]; STATA [20]; SAS [21].	Useful for descriptive illustration of event distributions over time.
	Cumulative incidences/Transition probabilities	Aalen-Johansen estimate [41, 42]	Non-parametric; estimates cumulative incidences and transition probabilities.	Markov assumption; same clinical relevance for endpoints within the composite.	R etm::etm [51]; STATA [20]; SAS [21].	Useful for descriptive illustration of cumulative incidences or transition probabilities over time.
		State occupation probabilities via the Aalen-Johansen estimate [41, 42]	Non-parametric; describes average length of stay in a recurrent event state and the mean number of recurrent events.	Same clinical relevance for endpoints within the composite.	R etm::etm [51].	Useful for descriptive illustration of the average time spent in event states.

### Composite endpoints

A fundamental question is whether to combine distinct clinical endpoints (e.g., hospitalizations, death) into a composite endpoint. This strategy is frequently used to improve statistical efficiency by increasing the overall event rate. Furthermore, composite endpoints combine different clinical events that all have similar impact on patients’ lives. Composite endpoint methods can broadly be divided according to whether the clinical relevance of each endpoint should be considered or not.

#### Methods that do not consider the clinical relevance of endpoints

These methods treat all endpoints as equally relevant. Common approaches include the following:

##### Andersen-Gill (AG) model

**Fig. 3:**
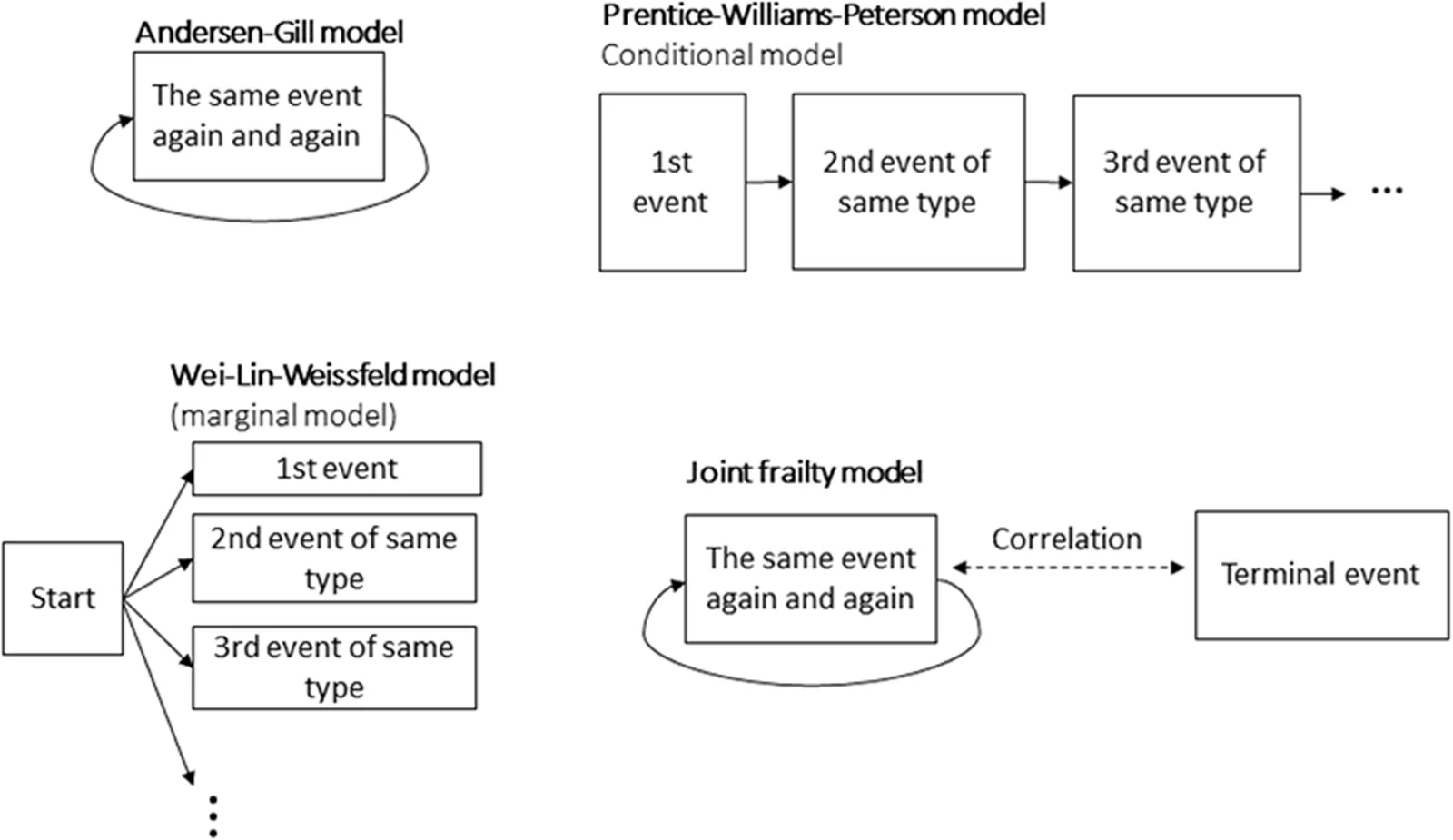
Model illustration

**Introduction** The AG model [5] is a semi-parametric extension of the Cox proportional hazards model [3] for analyzing recurrent time to event data. In the model, all individuals remain at risk for an event as long as they are under observation, i.e. also after experiencing an event. The method was described for the situation where only one endpoint is of interest, i.e. the same event occurs over and over again. Figure 3A illustrates the corresponding setting. However, we are interested in multiple different endpoints. Ozga et al. [7] described and evaluated the method in the setting of a composite endpoint with two components (=endpoints). For example, if we are interested in recurrent myocardial infarction and death due to cardiovascular reasons, all recurrent events per patient are included, and the fatal event of death ends the recurrent event process but is included as the last event for the corresponding individual. In case of competing events, e.g. another cause of death which is not part of the composite endpoints, it is dealt with as independent right-censoring, which seems appropriate for our setting of interest because Troendle et al. [52] argued that for randomized controlled trials the effect estimation via a Cox-type model leads to less/no bias compared to a model using subdistribution hazards. Thus, in this composite-endpoint setting, the AG model addresses whether treatment changes the overall intensity of the composite recurrent-event process.

**Estimand** The target quantity is the treatment contrast in the event intensity among individuals who remain under observation in the AG risk set. It is expressed as a hazard ratio comparing the intervention and control groups. When several event types are combined in one composite endpoint, the resulting all-cause hazard ratio refers to the selected composite endpoint as a whole and not to the individual components separately [7].

**Advantages/Limitations** The proximity of the AG model to the Cox model as a commonly applied and hence well-understood method is an advantage, also for interpretation since the well-known hazard ratio is the main outcome often applied for the analysis in clinical studies. Ozga et al. [7] showed that the AG model can be applied quite well in the setting of composite endpoints. Moreover, if hospitalization is one event of interest, one can incorporate that while a patient is in hospital, the person is not at risk for hospitalization.

Because individuals remain at risk after previous events, the estimate may also be influenced by how previous events affect subsequent event risk. Hence, if the Markov assumption (i.e. the transition hazards do not depend on the previous event, the number of previous events, or previous event times) is not fulfilled, the treatment effect cannot easily be parameterized and might therefore be considered difficult to interpret. Interpretation can also be problematic if the treatment effects within the components point in different directions. In such situations, or when components differ substantially in clinical relevance, a single overall hazard ratio may mask clinically important heterogeneity between components. If component-specific treatment effects are of primary interest, additional component-specific analyses are required.

Another advantage is that it is implemented in standard statistical software and hence easy to use.

##### Lin-Wei-Ying-Yang (LWYY) model

**Introduction** The model by LWYY [22] is an extension of the AG model in that it widens the class of rate functions. In contrast to the AG model, the LWYY model allows for between-patient heterogeneity. The point estimates from AG and LWYY will be the same, but standard error estimates will differ if there is dependence among recurrent events for an individual. Since the LWYY model includes a wider class of rate functions than the AG model, the LWYY model is often referred to as the AG model with a robust variance estimator. In a composite endpoint, the endpoints of interest are combined into one. In case of competing events, it is dealt with as independent right-censoring. Thus, in the composite-endpoint setting considered here, the LWYY model addresses whether treatment changes the marginal rate function of the selected components analyzed together as one composite event process.

**Estimand** The target estimand is a marginal treatment contrast in the rate function of the composite event process. It is expressed as a rate ratio comparing the intervention and control groups; when several event types are combined, this corresponds to an all-cause rate ratio.

**Advantages/Limitations** The LWYY model has the advantage that it allows valid inference in the presence of within-patient dependence between recurrent events by using a robust variance estimator. However, it assumes a time-constant rate ratio; if this assumption is not met, the resulting ratio estimate cannot be interpreted as a simple average of time-specific rate ratios and has no direct clinical interpretation. In composite endpoints, the all-cause rate ratio for the composite event process may mask clinically important differences between components, especially if component-specific treatment effects differ substantially or point in different directions. The model is implemented in standard statistical software.

##### Parametric time to event model with all events of all endpoints

**Introduction** Analogously to the semi-parametric estimation within the AG model, a parametric time to event model can be used for effect estimation, e.g., the accelerated failure time (AFT) model. Thereby, the event distribution (including all events that occur per individual) is estimated under the assumption of a specific distribution like a Weibull, log-normal, log-logistic, or Gompertz distribution. In the setting considered here, this approach addresses a similar composite recurrent-event question as the AG model, but describes the composite event-time process through a specified parametric distribution.

**Estimand** The target estimand is the treatment contrast in the time scale of the composite event-time process. In an AFT formulation, this contrast is expressed by the acceleration factor (AF), which indicates whether event times in the intervention group are stretched or shortened relative to the control group. In case of multiple events per patient, the AF refers to all selected event times analyzed together.

**Advantages/Limitations** The parametric time to event model is a flexible approach and can be used when the proportional hazards (PH) assumption is not appropriate for the composite endpoint; i.e. if the PH assumption is not fulfilled for the composite endpoint, it may be considered instead of the semi-parametric approaches. Its parametric structure allows direct estimation of event-specific hazard functions and effects, enabling prediction of event-free probabilities. However, the model relies on correct specification of the underlying parametric distributions. The same clinical relevance for the endpoints combined is assumed as well as the same treatment effect for all components within a composite endpoint. Consequently, the AF provides one overall time-scale contrast for the composite endpoint and does not distinguish between the individual components. Standard statistical software can be used for this approach.

##### Prentice-Williams-Peterson (PWP) model

**Introduction** The PWP model [6] is another extension of the Cox proportional hazards model including all events experienced by an individual. In contrast to the AG model, the PWP model orders recurrent events and defines separate risk sets according to the number of previously experienced events. It is therefore a conditional or event-order-specific model. In this extension, strata are incorporated: in the first stratum, all first events are included; in the second stratum, all second events; and so on. Figure 3B illustrates the corresponding setting. As in the AG model, the PWP model was described for the situation where only one endpoint is of interest. Since we are interested in the combination of different events, we base our description within this work on the work by Ozga et al. [7], who described the method in the setting of composite endpoints and evaluated the method for a composite endpoint with two components. As in the AG model, all recurrent events are included and, if a fatal component is part of the composite endpoint, it is included as the last event. A competing event is treated as independent right-censoring.

**Estimand** The target estimand is a conditional treatment contrast in the hazard of the next composite event within event-order strata, among individuals with the same number of previous composite events who remain under observation. It is expressed as an all-cause hazard ratio for the selected composite endpoint, as in the AG model.

**Advantages/Limitations** This method is easy to use since it is implemented in standard statistical software and uses the well-known hazard ratio for treatment effect description. However, in the composite endpoint, the same clinical relevance is assumed for all components, and the overall treatment effect is a combination of indirect and direct effects, which might be difficult to interpret. Despite this, Ozga et al. [7] showed that the PWP model can be recommended for the analysis of composite endpoints with recurrent events. Also because it considers that the occurrence of a subsequent event might be influenced by the occurrence of a previous event, which is more likely to be the case in clinical practice. Events can be distinguished such that strata specific baseline hazards are assumed and one could also gain strata specific treatment effects. Thereby, the number of events should not be too small per stratum. Event types could be distinguished if one assumes a stratum for e.g., the fatal event alone (thereby different cause-specific baseline hazards can be used). Interpretation can be problematic if the treatment effects within the components point into different directions.

##### Stratified parametric time to event model

**Introduction** The stratified parametric time to event model is a parametric extension of the semi-parametric Prentice-Williams-Peterson model, i.e. an AFT model can be used including strata for the number of events, as in the PWP model.

**Estimand** See Section for the parametric model above.

**Advantages/Limitations** See Section for the parametric model above. Additionally, in the stratified approach, we consider the order of events.

##### Wei-Lin-Weissfeld (WLW) model

**Introduction** The WLW model [26] is an extension of the Cox PH model and uses like the PWP model strata where in the first stratum are all first events in the second stratum are all second events, and so on. The difference from the PWP model is that an individual is at risk for all events; i.e. although an individual did not have a second event, it is at risk for a third event (marginal model). Figure 3C illustrates the setting. In this work, the focus is on multiple events and thus we will use the definition of a composite endpoint as described by Ozga et al. [7]. As in the models mentioned above, all recurrent events and the fatal event as the last event are included, and a competing event is modeled as independent right-censoring.

**Estimand** The target estimands are marginal event-order-specific treatment contrasts for the composite event process, with event-order strata defined by the order of occurrence. In a proportional hazards formulation, these contrasts can be expressed as event-order-specific HRs.

**Advantages/Limitations** The WLW model is implemented in standard statistical software. Event orders can be distinguished such that stratum-specific baseline hazards are assumed, and one could also obtain stratum-specific treatment effects. Thereby, the number of events should not be too small per stratum. In the WLW model, the average HR is gained via a combined average regression coefficient by means of a simple linear combination of the strata specific parameters. Because each individual contributes to all event-order strata from the start of follow-up, the WLW risk sets are less intuitive than the conditional risk sets used in the PWP model. A limitation is that, when components are combined into one composite endpoint, component-specific baseline hazards and component-specific treatment effects are not directly distinguished. Besides the fact that the model does not capture a realistic scenario in the simulation study, higher standard deviations were seen as compared to the AG or PWP model. Additionally, interpretation can be problematic if the treatment effects within the components point in different directions. In such situations, a combined marginal hazard ratio may mask clinically important heterogeneity across components or event-order strata.

##### Negative binomial (NB) model

**Introduction** One often applied method in recurrent event analysis is the NB model (see Fig. 3D) [28]. Thereby, the events per patient are counted but time to event occurrence is not considered. Time can only be considered via the offset, for example, individual study duration. Thus, in the setting considered here, the NB model addresses whether treatment changes the number or rate of selected recurrent or composite events during follow-up.

**Estimand** In this model, the (all-cause) incidence rate ratio (IRR) between groups, i.e., the ratio of event counts including all events of primary interest for an individual, is estimated.

**Advantages/Limitations** The negative binomial model is implemented in standard statistical software. Since this is not a time-to-event method, event and censoring times can only be considered via the offset as individual study duration. Consequently, the model does not describe when events occur or how previous events affect later events. In this model, it is problematic if too many individuals have zero events, and a model that handles zero inflation may be needed. Event types or competing events are not distinguished and clinical relevance is ignored. Death or another terminal event can only be reflected through the observed follow-up time used in the offset unless it is included as part of the counted outcome. The assumption of a constant patient-specific incidence rate across time may be unrealistic for a condition that progresses.

#### Methods that consider the clinical relevance of endpoints

The clinical relevance of components in a composite endpoint can be considered via two approaches: weighting of the components/endpoints or ordering the combined endpoints.

**The weighted methods are:**

In these methods, the weights are part of the endpoint definition and therefore influence the interpretation of the treatment effect.

##### Weighted Lin-Wei-Ying-Yang (WLWYY) model

**Introduction** Mao et al. [30] introduced a weighted effect measure based on a semi-parametric model (extension of LWYY). They define a weighted composite endpoint as the cumulative number of recurrent and terminal events weighted by the relative severity of each event. All events of primary interest are combined but weighted according to their clinical relevance. Competing events are assumed as independent right censoring.

**Estimand** The rate (or mean) ratio between groups for the weighted composite event process (weighted rate ratio) is of interest. This estimand summarizes the selected recurrent and terminal event components according to their prespecified weights.

**Advantages/Limitations** The method provides one weighted treatment-effect summary for all events, which also includes the clinical relevance of endpoints. Thereby, the dependency structure between endpoints is unspecified. See LWYY for further advantages/limitations. However, as for all weighted methods, the weighting scheme can influence the interpretation of the treatment effect and the statistical power and should be specified a priori, which might be difficult. The method is implemented in R [31].

##### Parametric weighted all-cause hazard ratio (WHR)

**Introduction** The WHR was introduced by Rauch et al. [8]. Thereby, non-negative pre-specified cause-specific weights are multiplied with cause- and group-specific hazards. These cause- and group-specific hazards can be estimated via a parametric estimation approach. The approach was first described for a composite endpoint, where the time to the first occurring event per individual was of interest. Thereby, the clinical relevance of components for the composite was considered by assigning weights to the cause-specific hazards. Ozga et al. [10] described the extension to include possible recurrent events. In case of competing events, they are dealt with as independent right-censoring. Thus, the method addresses whether treatment groups differ with respect to the clinically weighted composite hazard.

**Estimand** The WHR is defined as the ratio between the weighted hazard of the intervention and the weighted hazard of the control group. Thereby, the weights are assigned to the cause-specific hazards. The estimand is therefore a treatment contrast in a prespecified weighted sum of cause-specific hazards at the time point of interest.

**Advantages/Limitations** The approach is not implemented in statistical software yet but standard software can be used to do so. Confidence intervals can only be gained via bootstrapping. One has to define a specific time point at which the effect is of primary interest. As the hazard function estimator depends on the number of observed events, a high weight can still have a low impact if the underlying event rate is small. Thus, the composite effect is determined by the distribution of the clinically more relevant event. The clinical relevance of the endpoints is considered. However, the weighting scheme can influence the interpretation of the WHR and the statistical power and should be specified a priori, which might be difficult.

##### Parametric stratified WHR

**Introduction** In the parametric WHR, it can be incorporated that a previous event might influence the occurrence of subsequent events, i.e. stratification like in the PWP model was introduced. Thus, compared with the non-stratified parametric WHR, this approach allows the weighted treatment effect to be evaluated within event-order-specific strata.

**Estimand** See parametric WHR.

**Advantages/Limitations** See parametric WHR. Additionally, the order of events is considered through the stratified risk-set construction.

##### Non-parametric WHR

**Introduction** Ozga et al. [9] introduced a non-parametric estimation for the WHR. Thereby, the cause-specific cumulative hazards are used (estimated via the non-parametric Nelson-Aalen estimate). The main difference to the parametric WHR is therefore the non-parametric estimation of the cause-specific cumulative hazards, while the clinical weighting principle remains the same.

**Estimand** See parametric WHR.

**Advantages/Limitations** See parametric WHR. However, there is one more limitation: proportional cause-specific hazards are assumed for the estimation, but Ozga et al. [9] showed that the method is robust against deviations from this assumption.

##### Wei-Lachin (WL) approach

**Introduction** Wei and Lachin and Lachin et al. [11, 12] described a weighted semi-parametric approach for the analysis of composite time to first event endpoints. Ozga and Rauch [10] showed how the approach can be extended to a composite endpoint including recurrent events. The idea is to weight the cause-specific effects with relevance weights within commonly known semi-parametric time to event methods. In case of competing events, it is dealt with as independent right-censoring. Thus, the method addresses whether treatment has a weighted summary effect across the selected endpoint-specific treatment effects, with the weights reflecting their prespecified clinical relevance.

**Estimand** The target estimand is a weighted summary of the cause-specific treatment effects for the selected endpoint components, comparing intervention and control. The weights are applied to the log-transformed cause-specific hazard ratios and not directly to the hazard functions or event counts.

**Advantages/Limitations** One can use standard statistical software to conduct the methods but there is no readily available function to conduct all steps of the method. Using the weights on the cause-specific effects, implies that the influence of the weight is independent from the underlying number of events, and as a consequence a high weight has a large impact even if the corresponding cause-specific hazard ratio is estimated based on a low number of events. The different clinical relevance of endpoints is considered through the prespecified weights. The resulting treatment-effect summary is not component-specific. See also Sections for the AG model for more advantages/limitations.

##### Stratified Wei-Lachin (WL) approach

**Introduction** The WL approach can be extended with strata as it is done, e.g., in the PWP model, i.e., in the first stratum are all first events, in the second stratum are all second events, and so on. This extension allows the weighted treatment-effect summary to account for the order of recurrent events through stratum-specific risk sets.

**Estimand** See section WL approach.

**Advantages/Limitations** See section WL approach and PWP model. Additionally, the order of events is considered.

##### Approach by Bakal

**Introduction** The approach by Bakal [32, 33] is a weighted non-parametric estimation method for the survival probabilities, i.e. it produces a weighted version of the Kaplan-Meier estimate where all (recurrent) events can be considered. A corresponding non-parametric test, i.e. a weighted version of the log-rank test, for the comparison of these survival probabilities was also described. The scientific question is whether the weighted event experience differs between treatment groups when recurrent events are incorporated according to their clinical relevance. Competing events are treated as independent right-censoring.

**Estimand** No underlying model-based estimand is defined.

**Advantages/Limitations** A key advantage of this approach is that the clinical relevance of the endpoints is considered. However, the method has several fundamental limitations. It is based on so-called “weighted survival functions”, but the weighting scheme is only described at the estimation level. Furthermore, as no underlying model with a clear estimand is defined, it remains unclear what the method actually estimates, thereby making the formulation of formal test hypotheses impossible. Consequently, the interpretation is ambiguous: the method yields only a p-value for group comparison, without providing a quantifiable treatment effect. In addition, the method is not currently implemented in standard statistical software. Due to these conceptual and practical drawbacks, this approach is not recommended by Ozga et al. [10].

**Methods that use ordered endpoints are:**

##### Win ratio (WR) and win odds (WO)

**Introduction** Within the WR [15] or WO [34, 53] analysis, different endpoints are combined and ordered hierarchically, i.e. ordered depending on the clinical importance. The idea of the WR/WO approach is that one compares each patient in the treatment group to each patient in the control group regarding the endpoints of interest. For each patient pair, a winner, loser, or tie is assigned and the proportion of winners and losers is compared between groups. One compares the patients first within the first endpoint and if a winner/loser within this endpoint cannot be found, the next endpoint is considered. In the example of (recurrent) time to event endpoints, one could first define the winners as patients experiencing longer time to events and if that is inconclusive, one could define the winners within a next endpoint as having fewer events, while losers experience earlier events or more events. Ties occur when the time to event or event count is equal. Comparisons may sometimes be impossible due to censoring before an event occurs; such cases are ignored in the WR/WO analysis. Thus, WR and WO address whether a randomly selected patient from the treatment group has a more favorable prioritized outcome than a randomly selected patient from the control group, according to the prespecified hierarchy and comparison rules.

**Estimand** It is of interest to compare treatment and control groups regarding the hierarchically ordered endpoints and evaluate the proportion of wins (in the treatment group). The proportion of wins is defined as the total number of wins in the treatment group divided by the number of losses (or wins in the control group).

**Advantages/Limitations** The method is implemented in standard statistical software. Defining the order of events is quite crucial since another order/definition can change the result considerably. While the hierarchical order, or severity, of events is considered, their timing is ignored in the analysis. Endpoint/event correlation is not considered, but the relevance of endpoints is. Although the endpoints are hierarchically ordered, each endpoint component of the WR/WO is weighted the same and effects have to point into the same direction. Competing events can only be handled as independent right censoring in the time to event endpoints but are ignored in the other endpoints. However, one could consider using it as an additional endpoint within the WR/WO. In the WR analysis, ties are problematic because they are ignored and hence a biased effect can result. It is recommended to use WO instead [34, 53]. An advantage of the method is the increased flexibility in the composition of the endpoint in allowing the use of different types of variables, including patient-reported outcomes in combination with time to event endpoints. A limitation of this approach is that a baseline imbalance in risk within an analyzed pair may bias the outcome e.g. if one compares a young woman with an old man. It is recommended to use matched pairs or a stratified approach in this case [54]. However, it might be difficult to determine all adjustment variables. In randomized trials of sufficient size, this should be no problem and hence the use of an unmatched design is appropriate. If many dropouts are encountered in a WR analysis, a sensitivity analysis that adjusts for censoring should be mandated, but this is still under research [54]. The WR approach was also extended to a semi-parametric approach to allow adjustment for e.g. confounders [55, 56].

##### Generalized pairwise comparisons of prioritized outcomes

**Introduction** Buyse [36] et al. extended the idea behind the U-statistic of the Wilcoxon-Mann-Whitney test [57] to perform generalized pairwise comparisons between two groups of observations, e.g several (time to event) endpoints. Peron [37] extended the method for the time to event setting with right-censored data. The several outcomes have to be ordered, i.e. what is the most clinically relevant endpoint and which should be examined afterwards. In generalized pairwise comparisons, each patient in the treatment group is compared to each patient in the control group with respect to the endpoints of interest. Each comparison is classified as favorable for the treatment, unfavorable, neutral, or uninformative (e.g., due to missing observations or censoring). The concept of “in favor” is analogous to the “winner” in the WR approach, i.e. one examines the number of observations which are in favor of the treatment. For example, if the time to event is longer in the treatment group than in the control group, the outcome is favorable for the treatment; if shorter, it is unfavorable; if equal, it is neutral. Similarly, for event counts, a smaller number of events in the treatment group is favorable, a larger number is unfavorable, and equal counts are neutral. Thus, this method addresses a similar pairwise-comparison question to WR and WO, but based on prioritized pairwise comparisons between patients.

**Estimand** It is of interest to compare the treatment and control groups regarding the ordered endpoints and evaluate which endpoints are in favor of the treatment group, i.e. the expected proportion of patients for which the outcome is better in the treatment group minus the expected proportion of patients for which the outcome is better in the control group (net chance of a better outcome). The target quantity is defined over all treatment–control pairs and is based on the probabilities that a pairwise comparison is favorable, unfavorable, neutral, or uninformative according to the prespecified endpoint hierarchy. One possible treatment-effect summary is the proportion in favor of treatment (PiF); another commonly used summary is the net chance of a better outcome.

**Advantages/Limitations** No assumption of independence is required for the variables capturing the outcomes of interest. If several outcomes are evaluated, one should consider adjusting for multiple testing to preserve the overall significance level of a study, e.g. the methods developed for group sequential testing can be used for generalized pairwise comparisons. Prospectively deciding on the respective priorities of different outcomes of interest is not only a major advantage, but also a potential difficulty, of generalized pairwise comparisons. Changing the hierarchy or the comparison rules changes the clinical question and the corresponding estimand. Interpretation can be difficult for time to event endpoints or continuous endpoints, since it is not straightforward as the proportion in favor depends on the underlying distribution (i.e. location and scale of parameters). One cannot state that the proportion in favor is equal to the proportion of individuals improved by treatment. The clinical relevance of endpoints is considered. Competing events are considered as independent right-censoring for the time to event endpoints but not for others (see WR). The method is implemented in R [38].

### Composite endpoint considering correlated competing events or no composite endpoints

#### Semi-parametric joint frailty (JF) model

**Introduction** The JF model, as described by e.g. [13, 14] models the recurrent event process and the fatal event process separately but correlates them via a so-called frailty as illustrated in Fig. 3E. The rationale is that the recurrent event process can be terminated with a dependent terminal event. Thus, the JF model addresses the question of whether treatment affects the recurrent-event process and the terminal-event process while accounting for their association through a shared latent frailty. In a setting of a composite endpoint with possible competing events, one could think of two possible model scenarios:

1. The recurrent event of the composite endpoint is used in the model part for the recurrent event process and the fatal event of the composite endpoint is used in the model part for the fatal event process. Hence, we allow the occurrence of the recurrent event to be correlated to the fatal event, e.g. more recurrent events lead to a higher risk for the fatal event. Furthermore, we gain two treatment effects: one for the recurrent event and one for the fatal event. This we can also refer to as no composite endpoint. The competing event is dealt with as independent right-censoring.
2. We could use all components of the composite endpoint in the model for the recurrent event process, making this part equivalent to the situation of using the AG model for the analysis of composite endpoints with recurrent events, as described in the section above. Hence, we will gain one treatment effect for the composite endpoint. For the model part of the fatal event process, we then use a competing event allowing it to be correlated to the composite endpoint (Composite endpoint considering correlated competing events).

**Estimand** The aim is to quantify the difference between intervention and control regarding the event rates using the ratio between the event rates described by the hazards, i.e using the hazard ratio. Within the JF model, two hazards/hazard ratios are modeled conditional on the latent frailty: one for the recurrent event process and one for the fatal event process.

**Advantages/Limitations** This approach accounts for dependence between event processes and unobserved heterogeneity. Because the association is modeled through a latent frailty, the treatment effects are conditional model-based effects and should not be interpreted as marginal population-average effects. For more advantages/limitations: see also AG model with the difference that one could model the competing event within the JF model in the terminal part, i.e. using the composite endpoint of primary interest in the recurrent event part which is then as in the AG model but one could also split the composite endpoint in its possible recurrent events and terminal events and use those in the JF model and hence consider competing events as independent right censoring. In the situation of “no composite endpoint”, one has to consider that since two effects are estimated, one has to adjust for multiple testing, i.e. using hierarchically ordered or multiple or co-primary endpoints. Convergence of the proposed model could be difficult to obtain in the case of data with few events [14, 39]. The JF model was only described for the situation of one maximum of two recurrent events and one fatal event, which is already quite complex, making further extensions unlikely. The JF model is implemented in standard statistical software.

#### Parametric JF model

**Introduction** Instead of the semi-parametric JF model, one can also use parametric estimation, e.g. using a Weibull distribution. The scientific question and endpoint structure are the same as for the semi-parametric JF model, but the baseline event-time distributions are specified parametrically.

**Estimand** Depending on the choice of parametric model, acceleration factors or hazard ratios are obtained for each of the endpoints to describe the difference in event rate between the treatment groups; these treatment-group contrasts are also conditional on the latent frailty.

**Advantages/Limitations** See semi-parametric JF model. However, the parametric JF model may be useful in case of non-proportional hazards, provided that the chosen parametric distribution is appropriate.

#### Multi-state model (MSM)

**Introduction** In MSM, one is interested in the transitions between events (=states). Thus, the required data structure consists of observed states and the times at which individuals move between these states. The MSM is a statistical framework that generalizes traditional survival analysis by allowing individuals to transition between multiple, discrete states over time rather than experiencing only one single terminal event [16]. It is particularly suited for analyzing complex disease or treatment processes where subjects may experience a sequence of events, such as remission, relapse, hospitalization, or death. The model describes the probability and rate of moving between these states, providing a dynamic representation of disease progression and treatment effects. Schmeller et al. [16] showed how the AG and PWP model can be translated into MSMs. An example (Figure) is given in the appendix.

**Estimand** Transition-specific hazard ratios are used to describe the group difference in event transition rates. The target estimand is therefore a treatment contrast for a specific transition from one state to another among individuals who are currently in the origin state and at risk for that transition.

**Advantages/Limitations** The MSM provides a flexible framework for capturing complex event histories, offering deeper insight into disease progression than single-event models. It enables decomposition of treatment effects across multiple pathways and quantifies intermediate outcomes that precede terminal events. The resulting estimates are transition-specific and should not be interpreted as one overall treatment effect across the whole disease process. However, its performance depends on accurate specification of the transition structure and sufficient data to estimate each transition hazard. When transitions are rare or follow-up is short, estimates may be unstable. Furthermore, multiple effects (for each event or endpoint transition) are estimated and one has to think about adjustment for multiple testing. Moreover, Markov assumptions may oversimplify biological reality if history or time since entry influences risk, and model interpretation can become complex as the number of states increases. Statistical software is available for MSM.

#### Ghosh and Lin model (GL)

**Introduction** If the Markov assumption of the AG model is not fulfilled, one could use the rate-based equivalent to the AG model, which is the rate model from Ghosh and Lin [43]. A marginal mean function for the cumulative number of recurrent events over time is considered. Thereby the fact that death precludes further recurrences is considered. A non-parametric estimation of the marginal mean function was also described earlier [58]. Thus, this method addresses whether treatment changes the marginal recurrent-event burden over time while accounting for the fact that death terminates the recurrent-event process.

**Estimand** The aim is to quantify the difference between intervention and control regarding the marginal cumulative number of recurrent events over time. The recurrent-event treatment effect is expressed as a rate ratio for the marginal recurrent-event process, with death treated as a terminal event that precludes further recurrences. Treatment effects for the recurrent-event process and the fatal event are considered separately.

**Advantages/Limitations** One advantage of the model is that the Markov assumption is not needed. In contrast to the JF model, the dependence among recurrent events or between recurrent events and death does not need to be specified. Since two effects are estimated, one has to adjust for multiple testing, e.g. by using a hierarchical testing strategy or by defining multiple or co-primary endpoints. Ghosh and Lin [43] did also described testing procedures, e.g. sequential testing. In contrast to the AG model, the Ghosh and Lin model cannot model that a patient already in hospital cannot be at risk for hospitalization. The model requires modeling either the survival or censoring distributions which is not very appealing as such models may be misspecified. The model is also implemented in standard statistical software.

#### Area under the curve (AUC)

**Introduction** An extension of restricted mean event-free survival time for repeated events is the AUC [46]. Thereby, the average event-time for the treatment groups is set into relation. The AUC method evaluates the cumulative burden of recurrent events by choosing a fixed time point for assessment. For each event, the time elapsed between the event occurrence and the fixed time point is calculated and summed across all events for each patient. This sum represents the average event-time, which corresponds to the area under the cumulative event rate curve—a curve displaying the mean number of events per patient, adjusted for loss to follow-up. The average event-time is then compared between treatment groups. Thus, this method addresses whether treatment changes the cumulative recurrent-event burden up to a prespecified time point.

**Estimand** The interest lies in the difference or ratio between groups regarding the total time after event occurrence until a fixed time point. The target quantity is the area under the mean cumulative event-rate curve up to the fixed time point, summarizing the average cumulative event burden per patient over the chosen time horizon.

**Advantages/Limitations** An advantage is that the AUC is model-free in the sense that it does not require a proportional hazards or parametric event-time model. It is also robust and provides a clinically interpretable, time-scale summary of the treatment effect. A limitation is that one has to define a fixed time point for evaluations: in most trials, individuals have a range of follow-up times. Furthermore, the same clinical relevance for endpoints within a composite is assumed. Therefore, when applied to a composite endpoint, the AUC summary is not component-specific. The method is implemented in R [45].

#### ‘While-Alive’ Strategy (WA)

**Introduction** The idea for the while-alive estimand is that a treatment effect is estimated only for the period patients are still alive [48]. It compares recurrent events by considering the rate per unit time alive, correcting for situations where one treatment group lives longer but experiences more events overall. Thus, the method is suited for recurrent-event data with death as a terminal event, when the scientific question concerns event burden during the time patients are alive. Mao [47] extended the while-alive strategy and described a general class of while-alive estimands. A loss function is defined that measures the instantaneous loss due to new events with weights possibly dependent on the past. The while-alive loss rate is then defined as the cumulative loss averaged over the restricted mean survival time within the time window of interest. He also describes an approach where one can multiply the loss rate by the length of the time window to obtain the “survival-completed cumulative loss” [47].

**Estimand** The target quantity is the while-alive loss rate within a prespecified time window, defined as the cumulative loss from recurrent events averaged over the restricted mean survival time. The treatment contrast can be expressed as a loss rate ratio comparing intervention and control. It quantifies how the average loss, or average number of events, per unit time alive differs between treatment groups.

**Advantages/Limitations** A limitation is that the summary depends on the chosen time window and loss function, so it should be interpreted as a while-alive summary rather than as an unrestricted overall treatment effect. An advantage is that the Markov assumption is not needed. However, the causal interpretation still has to be discussed. The method is implemented in R [49]. If no differential weights are specified in the loss function, components within a composite contribute with the same clinical relevance.

### Further data description

**Introduction** Non-parametric methods to visualize the data like transition probabilities or state occupation probabilities were also described [16].

#### Nelson-Aalen (NA) estimate and Aalen-Johansen (AJ) estimate

**Estimand** For the NA estimate: Cumulative (cause-specific) hazard or transition hazards for treatment groups.

For the AJ estimate: Cumulative incidences and transition probabilities for treatment groups [41, 42]. Thus, the target quantities are treatment-group-specific descriptive quantities; no model-based treatment-effect parameter is estimated.

**Advantages/Limitations** The methods are implemented in standard statistical software. The methods are only for illustrating the results but no effect estimate or p-value is gained. The events are differentiated via the different plots for each transition, i.e. one has no overall description but a more detailed overview of event distribution. For more complex multi-state models with e.g. many event transitions enough events are needed for each transition for reliable estimation. In case of composite endpoints, the same clinical relevance for endpoints is assumed.

#### State occupation probabilities

**Estimand** State occupation probabilities describe the probability of being in a specific state at a given time point. With the state occupation probability, one can describe the average length of stay in a recurrent event state up to a specific time point and the mean number of recurrent events [59]. These are treatment-group-specific descriptive quantities rather than treatment-effect parameters.

**Advantages/Limitations** The method yields a simpler interpretation than rates or intensities. One can use the AJ estimate but without the Markov assumption. However, as for the NA and AJ estimates, the method is only used for data description and no effect estimate or p-value is gained. The method is implemented in standard statistical software. In case of composite endpoints, the same clinical relevance for endpoints is assumed.

### Further approaches

Further methods that were described in the literature for the analysis of multiple time to events can be found in the appendix. We did not describe these methods further since they are either different concepts (e.g. Bayes, pseudo-observations), not of primary interest (additive models), not implemented in standard statistical software or not easily implemented [60–63], and are basically the foundation of other methods described in the previous sections (e.g. [64]).

## Application

### Data set

We apply the methods described above to data from the Early Treatment of Atrial Fibrillation for Stroke Prevention Trial (EAST-AFNET 4) [2] for illustration. The study is an international, investigator-initiated, parallel-group, open, blinded-outcome assessment trial. Patients who had early atrial fibrillation and cardiovascular conditions were randomly assigned to either receive early rhythm control or usual care (allocation ratio 1:1). 2789 patients were randomized at 135 sites in 11 countries. In the control group, 1394 patients were included, and 1395 in the treatment group.

The study was funded by the German Ministry of Education and Research and others; EAST-AFNET 4 ISRCTN number, ISRCTN04708680; Clinical-Trials.gov number, NCT01288352; EudraCT number, 2010 −021258-20. The protocol was approved by the ethics review boards of all the institutions involved. Written informed consent was provided by all patients who participated in the trial. For more details, see also [2, 65].

The first primary outcome was a composite time to first event endpoint including the events of death from cardiovascular causes, stroke, or hospitalization with worsening of heart failure or acute coronary syndrome. 249 primary events were observed in the treatment group and 316 in the control group. Comparing the groups via a Cox proportional hazards model within time to first event analysis resulted in a hazard ratio of 0.78 (95*%* confidence interval: 0.66–0.92). In the present work, we want to include all events of primary interest that were observed for the individuals in the analysis, i.e. we are primarily interested in the composite endpoint with inclusion of all recurrent events. Within a median follow-up of 5.1 years, the following number of events were observed: 140 hospitalizations with acute coronary syndromes (ACS), 161 cardiovascular (CV) deaths, 112 strokes, 521 hospitalizations with worsening heart failure (WHF). Furthermore, 141 competing non-CV deaths were observed.

We further decided the following: we evaluate the treatment effect at the last observed time point (for approaches where a constant treatment effect over time is assumed this is irrelevant; relevant for: weighted all-cause hazard ratio (WHR); AUC and while-alive estimates).

For the parametric approach, we assume Weibull-distributed event times.

Within the weighted methods, we used the following weighting scheme for our primary analysis: within the WHR, the approach by Bakal et al., and weighted LWYY, the weights are as follows: CV death gets the full weight of 1, WHF and ACS get half of that, i.e. 0.5, and stroke gets 0.8. This corresponds to the following weights in the Wei-Lachin approaches: 0.36, 0.18, 0.29, respectively.

Within the WO/WR and the method for generalized pairwise comparisons of prioritized outcomes, we used the following order of endpoints for our primary analysis: 1. time to CV death, 2. no. of strokes per patient, 3. time to last stroke (per patient), 4. no. of WHF per patient, 5. time to last WHF (per patient), 6. no. of ACSs per patient, 7. time to last ACS (per patient).

For the AUC and while-alive estimates, we assume that only non-CV death restricts the time being alive (CV death is part of the composite and hence assumed to be in the recurrent event process).

We assume that one is at risk for hospitalization although the individual is already in hospital to make comparison of methods more fair. Methods that could handle that a person is not at risk for hospitalization when he/she is in hospital are: AG, (W)LWYY, PWP, JF model, WL, WHR, MSM, and corresponding parametric methods.

### Results

**Fig. 4:**
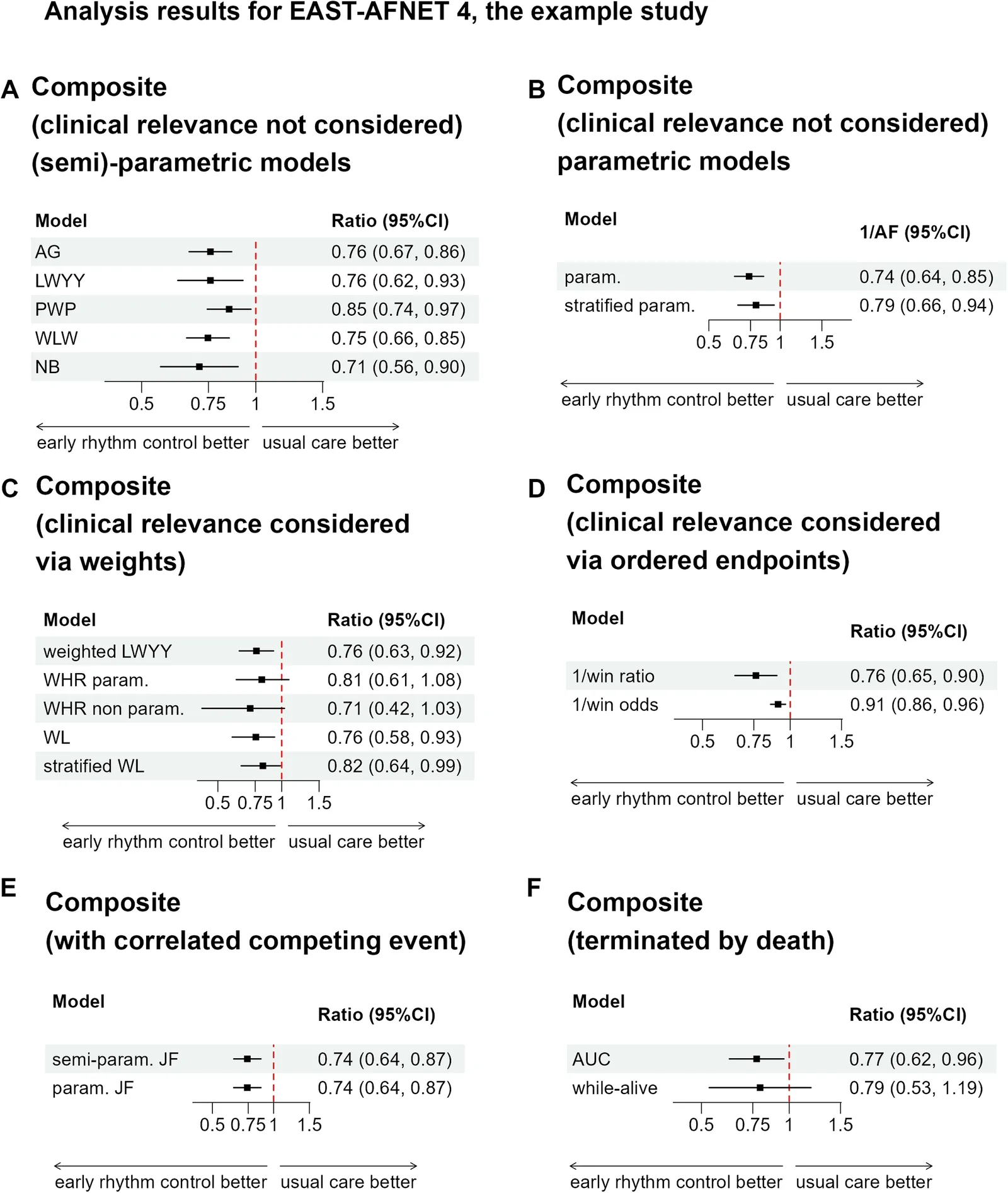
Model results: AG=Andersen-Gill; LWYY=Lin-Wei-Ying-Yang model; PWP=Prentice-Williams-Peterson; WLW= Wei-Lin-Weissfeld; NB=Negative binomial; param=parametric; WHR=weighted all-cause hazard ratio; WL=Wei-Lachin; JF=Joint-Frailty; AUC=Area under the curve; CI=confidence interval, AF=acceleration factor

**Fig. 5:**
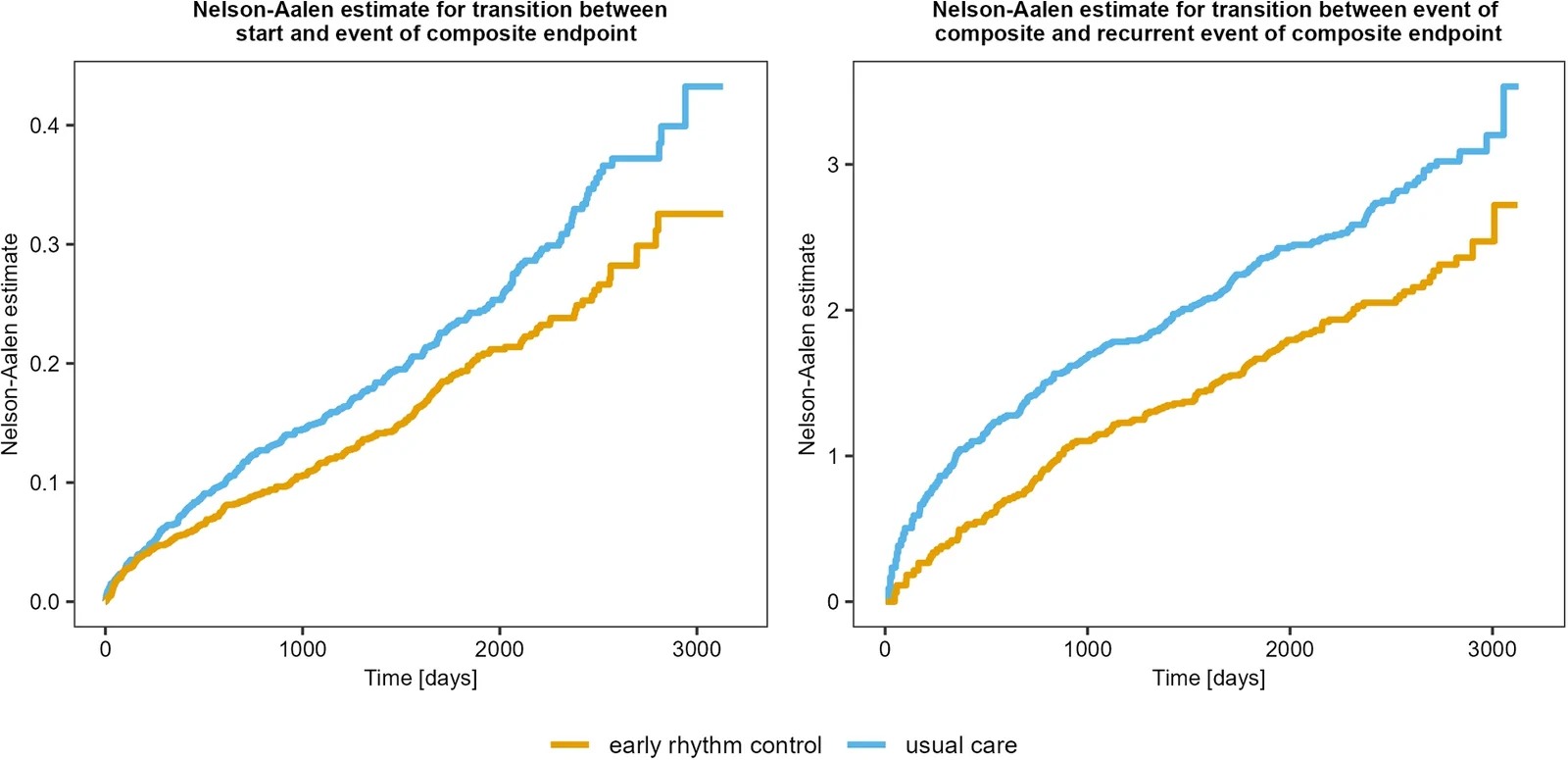
Nelson-Aalen estimate

**Fig. 6:**
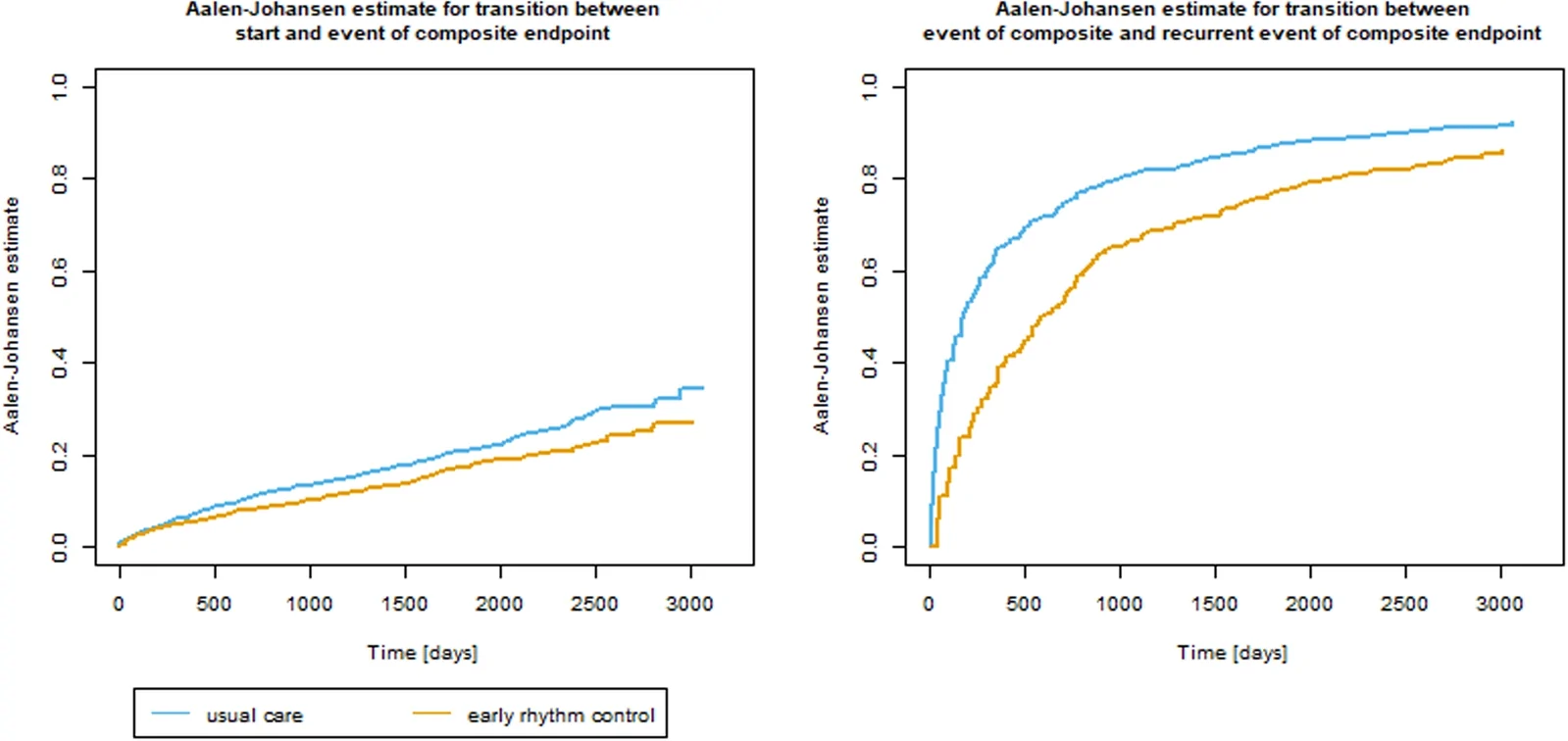
Aalen-Johansen estimate

Figure 4 presents treatment effect estimates from the different models. Figure 5 illustrates the NA estimates, and Fig. 6 illustrates the AJ estimates. A more complex scenario can be found in the appendix (i.e. all transitions). In the Appendix, further results can be found, i.e. effect estimates for a semi-parametric multi-state model and the model results where the recurrent events and CV death are not considered as one composite endpoint and hence two treatment effects are gained. To support interpretation of the estimates shown in Fig. 4, application-specific interpretations of the estimates are provided in Appendix 1, Table A1.

The treatment effects for the composite endpoint of interest point into the same direction for all approaches and are in favor of the treatment. They range from 0.71 for the negative binomial model or nonparametric WHR to 0.91 within the WO analysis (inverse of WO). For the models where unweighted HRs are gained (AG, PWP, WLW), the given HRs indicate that the hazard rate (for all events in the composite) changes with that factor between treatment groups. However, differences between those models are that the PWP model considers conditional event occurrence, and the assumption for the WLW model regarding the event connection is unclear. The treatment effect of the PWP model differs from the hazard ratio of the AG model, which might indicate event dependencies. The rate ratio of the LWYY model is the same as for the AG model (as expected) but a wider confidence interval is observed, i.e. between patient heterogeneity is observed. The interpretation of this result is similar to that of the AG model result. The model for the event counts shows an IRR of 0.71, indicating a lower rate of events in the treatment group by that factor. For the parametric or stratified parametric models for the unweighted composite, the AFs are 1/0.74=1.35 and 1/0.79=1.26 for treatment vs. control, respectively. This indicates that the time without an event is prolonged in the treatment group by these factors (Fig. 4 shows the inverse AF so that the effects point into the same direction for all figures).

Although the influence of the different event types differs in the weighted approaches compared to the unweighted composite, the effects do not change notably. However, the results differ for the WL approach and the weighted all-cause hazard ratio, so one has to decide whether the logartihmized cause-specific effects should be weighted or the cause-specific hazards. The latter is the WHR, which can be interpreted as the weighted average of cause-specific hazard ratios. For the WHR, one has also to note that the stratified parametric approach did not converge and that results between the parametric and non-parametric approaches differ. So do the WL and stratified-WL results, which are in agreement with the results from AG and PWP, since the WL/stratified WL approaches are extensions of those.

For the ordered endpoints, we only illustrate the WR/WO (Fig. 4 shows the inverse so that it points into the same direction as in the other figures). The results indicate that there are more wins in the treatment group (proportional win rate comparing treatment vs. control for WR: 31*%*; for WO: 10*%*). The WR is quite different from the WO result, which might indicate many ties (in fact, the proportion of wins in the two groups is only: treatment: 20.18*%* and control: 15.38*%*). Hence, the WR is not recommended.

For the JF model (semi-parametric and parametric), the HRs coincide (note that the function in R for the parametric JF model shows only HRs and no AF). The HRs are also in line with the HRs from the other models, which might indicate that the influence of the competing non-CV death is small.

The NA and AJ estimates support those findings since the treatment curve is mostly below the control curve for the endpoints of interest. The other non-parametric methods do so as well (Bakal approach p-value: 0.002; effect (i.e. proportion in favor of treatment) within Generalized pairwise comparisons of prioritized outcomes: 0.0674 (95*%* CI: [−0.0635, 0.196]); although this is a small group difference). The AUC ratio and while-alive ratio are also similar. However, the confidence interval for the while-alive estimate is quite wide, indicating high variability in follow-up. For the AUC, the factor of 0.77 means that, on average, patients spent less time under increased disease burden, and the corresponding total event-free time lost was reduced relative to control. For the while-alive estimate, the ratio of 0.79 means that the rate of the composite events is lower in the treatment group compared to control by that factor (for those still alive).

Note that for some methods no results could be gained, i.e. the joint frailty model did not converge in one case (appendix) and for the Ghosh and Lin model CV death could not be defined as the last event of the recurrent event process (i.e. this process has to end with censoring).

In summary, analyses of repeated events and hierarchical ordering of the composite outcome confirm the main findings of EAST-AFNET 4. Repeated event analyses yield a slightly more favorable hazard ratio (around 0.75) compared to the main analysis (time to first event, HR 0.79), potentially suggesting a continued treatment effect. Individual analyses yield nuanced results due to different assumptions and different weighting of event types.

## Discussion

In this work, we gave an overview of methods that were proposed for the analysis of multiple time to event endpoints and showed how they are implemented in R. Throughout this overview, we aimed to provide a fair and neutral comparison of the available approaches, strictly avoiding any artificial bias towards a specific model, in line with the principles emphasized by Lange et al. [66]. A lot of methods are described for the analysis of recurrent events combined with a terminal event. To find the best methods for the research question of interest, one has to put a lot of thought into what the actual aim is, reflected by the estimand, and what data structure is to be expected, i.e. the clinical background should be known and e.g. underlying event distributions or event dependencies. One has to be clear about whether the aim is to gain one overall effect estimate for the whole disease process, whether thereby the different clinical relevance should be considered, whether several treatment effects for different endpoints are important to know to answer the research question, or whether a non-parametric description should be added. In standard competing-risk settings, in which a competing event precludes the subsequent occurrence of the event of interest, regression models for the cumulative incidence can also be considered. The Fine-Gray model targets the subdistribution hazard corresponding to the cumulative incidence and therefore addresses a different scientific question than a cause-specific treatment-effect analysis [67]. This may be useful for absolute-risk or prognostic questions, but it is not the main focus of the recurrent- and terminal-event framework considered here and its use for the primary efficacy analyses in randomized clinical trials is discussed controversially [52]. Also of importance is to be sure about whether the underlying model assumptions might be fulfilled and how they can be checked. The original publications on the methods often describe how the underlying model assumptions can be checked; e.g. the proportional hazards assumption can be checked via the Schoenfeld residuals [68] and the Markov assumption can be checked by including the number or time of previous events as covariates (overview of methods to check the Markov assumption: [69]). Although some methods might be robust against deviations from their assumptions, some others are not. For example, Schmeller et al. [16] showed that the Markov assumption is crucial, whereas the non-parametric weighted all-cause hazard ratio is quite robust against a deviation from its assumption [9]. However, a systematic evaluation of the robustness against model assumptions is lacking for most of the methods and is part of future research.

Using composite endpoints either in an unweighted or weighted way, also in the context of multiple event times can be appealing since a higher power could be gained and all information available in the data is used for decision-making about a treatment. However, it can be more difficult to interpret the results since it is a mixed effect of the treatment effect of different event types and an additional effect of previous event occurrence. Especially in the case of quite different cause-specific effects, the composite endpoint is not recommended. Choosing the weights could also be not easy. But one could define weights for the main analysis and use other weights within sensitivity analysis [9].

For the non-parametric WR/WO approach [15] or the method described by Buyes [36], the researcher should also be clear about the order of the endpoints of interest as this has a great impact on the result.

Using methods that result in more than one treatment effect, like a joint frailty model [14], the model by Ghosh and Lin [43, 58], or complex multi-state models might be appealing because they give the researcher additional insights into how the treatment might work differently within the disease process. However, these models often require more observations/events compared to the composite endpoint approach, e.g. some event transitions might occur too rarely to gain reliable results or one has to adjust for multiple testing in primary confirmatory analysis.

Non-parametric estimators like the NA, AJ estimates [41], or AUC [46] are always helpful as additional analysis to gain an overview over the disease process, since transitions between events and distributions are graphically displayed.

Some methods described were already (systematically) compared, focusing on basic composite endpoint structures. Ozga et al. [7] showed in a simulation study that the model by Wei, Lin, and Weissfeld does not perform well in the composite endpoint setting for one recurrent event and one fatal event. They recommended to use the AG or PWP model. The WHR [8], the WL approach [11] and the approach by Bakal et al. [32] were also systematically compared and the approach by Bakal et al. cannot be recommended, since only a p-value is gained and it is not clear to the researcher what the method actually does [10]. Building on these established findings, our analysis here evaluates whether these recommendations remain robust in the presence of increased complexity, specifically when modeling multiple types of correlated recurrent events and underlying patient heterogeneity.

Liu [70] compared the LWYY and the negative binomial model systematically. They found that the negative binomial model maintained the Type I error rate in all investigated scenarios with increased mortality rates, whereas with the LWYY the Type I error was not controlled for in all scenarios and hence recommended to be careful when interpreting the results.

Mao et al. [30] compared their weighted LWYY method also to the unweighted LWYY method and found that the weighted method results more often in unbiased effect estimates. They also argue that the marginal rate or mean function of recurrent events which consider the fact that there is no recurrent event after the terminal event and the joint modeling for the two types of events are either affected by the distribution of the terminal event or assume that a latent variable captures the dependence among recurrent events and between recurrent and terminal events, which is an assumption that cannot be proved.

Schmeller et al. [16] also showed how complex MSMs are connected to semi-parametric AG and PWP models, i.e. that for these semi-parametric models corresponding multi-state approaches can be described.

Gregson et al. [71] compared the time to first event analysis (Cox model) with the LWYY model, the NB model, the JF model, the WR, and the AUC ratio in several cardiovascular trials. They argue that it is also important to consider how meaningful it is to patients to include recurrent events into primary analysis. They also found that the gain in statistical power when using all recurrent events in the analysis does not seem to be given in clinical practice in all studies. Why this is the case needs further research.

Claggett et al. [72] found in their simulation study that the statistical power of both recurrent events and time to first event methods is reduced by increasing heterogeneity of patient risk and that the greatest gain in statistical power from use of recurrent events occurs in the presence of high patient heterogeneity and low rates of study drug discontinuation or crossover following the first event.

This was also confirmed by Fritsch et al. [73] who compared the power and type one error for the LWYY model, the NB model, the JF model, and the WR.

Further reading regarding the estimand framework for recurrent events in the presence of a terminal event can be found in [74, 75]. They also argued that conditional models like the PWP model might have no causal interpretation and hence should be interpreted carefully in terms of treatment effect evaluation over the whole treatment process. An alternative would be to either include covariates to relax the dependence on previous events or to use the strata-specific effects.

To sum up: The applied researcher is spoiled for choice if she/he is interested in the analysis of multiple time to event endpoints. The researcher has to be clear about the study aim and model assumptions to find the ’right’ model. This work should help the researcher to find the best method for her/his research question based on a comprehensive description of methods already proposed in the literature. It is always possible to define one main analysis method and use others within sensitivity analysis. To help even better with the decision in the future, we will systematically compare the methods in our future research also with regard to model robustness against deviation from model assumptions and complex scenarios like several correlated endpoints and clustered data. Thereby, the different estimands of the methods have to be considered. Also of interest in future work is whether models can be extended to capture not only some of the data characteristics but also allow to be more flexible or can capture the whole complexity of the data like event dependencies, different clinical relevance of events, clustered data, and non-proportional hazards. Additionally, it is of interest in future work, how study planning, especially sample size calculation can be conducted for the methods described because not for every method this is straightforward.

## Conclusion

Analyses of recurrent events and hierarchical ordering of components of composite outcomes can provide valuable additional information in the analysis of clinical trials. Several methods for analysis of recurrent events have been proposed and evaluated. This overview of available analysis methods and their specific strengths and weaknesses will hopefully help researchers to select the most suitable analysis method for specific trials and research questions.

## Supplementary information

Additional file 1. Appendix 1. Format: PDF. Supplementary methodological information, including model specification, estimation, interpretation, and underlying assumptions, as well as an application-specific interpretation table for the EAST-AFNET 4 example.

Additional file 2. Appendix 2. Format: PDF. R code used for model implementation, including data preparation and implementation-level data structures required for model fitting, corresponding output, and supplementary results.

## Data Availability

The datasets generated and/or analysed during the current study are not publicly available due to confidentiality restrictions related to the original clinical trial data and sponsor authorisation requirements, but may be available from the corresponding author on reasonable request and subject to approval.

## Abbreviations

ACS: Acute coronary syndrome
AF: Atrial fibrillation
AFT: Accelerated failure time
AG: Andersen-Gill
AJ: Aalen-Johansen
AUC: Area under the curve
CI: Confidence interval
CV: Cardiovascular
HR: Hazard ratio
IRR: Incidence rate ratio
JF: Joint frailty
KM: Kaplan-Meier
LWYY: Lin-Wei-Ying-Yang
MSM: Multi-state model
NA: Nelson-Aalen
NB: Negative binomial
PH: Proportional hazards
PWP: Prentice-Williams-Peterson
RR: Rate ratio
WA: While alive
WHF: Worsening heart failure
WHR: Weighted hazard ratio
WL: Wei-Lachin
WLW: Wei-Lin-Weissfeld
WO: Win odds
WR: Win ratio
