# Supplementary material for: Methods for analyzing recurrent and terminal events considering composite and competing endpoints in clinical trials: a practical overview
Source: BMC Med Res Methodol. 2026 Sep 12;26:206. doi: 10.1186/s12874-026-03008-7 (PMC13571326; doi:10.1186/s12874-026-03008-7)
Supplement: Supplementary file 1 [file 12874_2026_3008_MOESM1_ESM.pdf]

Appendix 1: Methods for Analyzing Recurrent and Terminal Events considering composite and competing endpoints in Clinical Trials: A Practical Overview

Alidan Duoerkongjiang[1*], Antonia Zapf[1], Paulus Kirchhof[2], Ann-Kathrin Ozga[1*]

[1]Institute of Medical Biometry and Epidemiology, University Medical Center Hamburg-Eppendorf, Hamburg, Germany.
[2]Department of Cardiology, University Heart and Vascular Center Hamburg, University Medical Center Hamburg-Eppendorf, Hamburg, Germany.

*Corresponding author(s). E-mail(s): d.alidan@uke.de; a.ozga@uke.de

1 Introduction

In this appendix, further key aspects of the methods mentioned in the main part are described. Some further approaches are introduced as well. In addition, an application-specific interpretation table is provided to clarify how the estimates obtained in the EAST-AFNET 4 application can be understood.

2 Notation

Throughout the appendix, let $i = 1, \ldots, n$ index individuals. The prespecified composite endpoint (CE) consists of K component endpoints, indexed by $k = 1, \ldots, K$. The maximum follow-up time is denoted by τ, and $t \in [0, \tau]$ denotes a time point at which the relevant event process or treatment effect is evaluated.

Treatment assignment is represented by X_i, with $X_i = 0$ for the control group and $X_i = 1$ for the intervention group. For methods considering event order, let $j = 1, 2, \ldots$ denote the order of an individual's observed events, with $j = 1$ corresponding to the first event occurs, $j = 2$ to the second, and so on.

3 More aspects of the methods

3.1 Composite Endpoints

3.1.1 Methods that do not consider the clinical relevance of endpoints

Andersen-Gill (AG) model

Model specification As in the Cox model, the Andersen-Gill (AG) model combines the baseline hazard, which describes the baseline hazard of experiencing an event in the control group, with the treatment-effect parameter, which quantifies the difference between groups (i.e., the hazard ratio, HR). The difference from the conventional Cox model is that, after a non-fatal event, an individual remains at risk for subsequent events as long as the individual remains under observation [1]. Thus, the hazard for the composite endpoint is

$$\lambda_{CE,i}(t) = \lambda_{CE,0}(t) \exp(\beta_{CE} X_i), \tag{1}$$

where $\lambda_{CE,i}(t)$ is the hazard for individual i, $\lambda_{CE,0}(t)$ is the baseline hazard, and β_{CE} is the parameter comparing the intervention with the control group.

For the two-group comparison, it holds that

$$HR = \frac{\lambda_{CE,0}(t) \exp(\beta_{CE})}{\lambda_{CE,0}(t)} = \exp(\beta_{CE}). \tag{2}$$

Estimation The AG model estimates the all-cause hazard ratio for the composite endpoint (including all endpoints of interest) while accounting for independent right-censoring. It uses a partial likelihood–based method for effect estimation. Each observed event contributes to the overall likelihood. For the risk set, all individuals who are still under observation at the time of an event are included.

Interpretation The interpretation of the hazard ratio is the same as in the Cox proportional hazards model, i.e., for a comparison between an intervention group and a control group:

- $HR > 1$: the hazard rate of events (all types) is increased for the intervention group.
- $HR = 1$: no difference in hazard rates between groups.
- $HR < 1$: the hazard rate of events (all types) is decreased for the intervention group.

A corresponding two-sided null hypothesis can be formulated to test for differences between the two groups:

$$H_0 : \text{HR} = 1 \quad \text{vs.} \quad H_1 : \text{HR} \neq 1$$

Inference is made using the partial likelihood applied in the estimation step.

Assumptions Since the AG model is based on the Cox proportional hazards model,

the proportional hazards assumption also applies, i.e. the treatment effect remains constant over time. This assumption is made for the total event process, assuming equal cause-specific baseline hazards. Furthermore, it is assumed that the instantaneous risk of experiencing an event at a specific time since study entry is the same regardless of whether previous events have occurred. This implies that recurrent events are assumed to be independent, which is a strong assumption. This is also known as the Markov assumption, meaning that the transition hazard (i.e., the transition between events) does not depend on previous events, the number of prior events, or their timing. In the context of composite endpoints, the AG model assumes equal clinical relevance for all components combined. Events are not distinguished, and the same treatment effect is assumed for all components.

Lin-Wei-Ying-Yang (LWYY) Model

Model specification

The Lin-Wei-Ying-Yang (LWYY) model is a proportional rates and means model. It can be viewed as a relaxation of the AG intensity model, allowing for arbitrary dependence structures among recurrent events. The proportional rate model for the composite endpoint can be expressed as [2]:

$$d\mu_{CE}(t \mid X_i) = d\mu_{CE,0}(t)\exp(\beta_{CE}X_i) \tag{3}$$

where $\mu_{CE}(t \mid X_i) = E\{N_{CE}(t) \mid X_i\}$ is the marginal mean cumulative number of composite-endpoint events up to time t, $N_{CE}(t)$ is the corresponding counting process, $\mu_{CE,0}(t)$ is the baseline mean cumulative event function, and β_{CE} is the parameter comparing the intervention with the control group.

Since the treatment indicator X_i is time-invariant, the corresponding proportional means model is

$$\mu_{CE}(t \mid X_i) = \mu_{CE,0}(t)\exp(\beta_{CE}X_i). \tag{4}$$

For the two-group comparison, the treatment-to-control rate ratio (RR) is

$$RR = \frac{\mu_{CE}(t \mid X = 1)}{\mu_{CE}(t \mid X = 0)} = \exp(\beta_{CE}). \tag{5}$$

Estimation The LWYY model estimates the rate ratio, including all endpoints of interest while accounting for independent right-censoring. Each observed event contributes to the overall estimation of event rates across treatment groups.

Interpretation The resulting rate ratio is interpreted analogously to the hazard ratio in the AG model. A corresponding two-sided hypothesis test can be formulated as:

$$H_0 : \text{Rate Ratio} = 1 \quad \text{vs.} \quad H_1 : \text{Rate Ratio} \neq 1$$

Assumptions The LWYY model requires that the ratio of the mean cumulative

count curves comparing the two treatment arms remains constant over time. This assumption is analogous to the proportional hazards assumption in the Cox and AG models. In the context of composite endpoints, the LWYY model assumes that all endpoints have the same clinical relevance. Events are not distinguished, and the same treatment effect is assumed for all components.

Prentice-Williams-Peterson (PWP) Model

Model Specification As in the Cox model, the Prentice-Williams-Peterson (PWP) model combines a baseline hazard with a treatment-effect parameter, which quantifies the difference between groups (i.e., the hazard ratio, HR).

In the PWP model, baseline hazards are stratum-specific, and stratum-specific treatment effects can also be estimated. The event order j defines the strata; an individual enters the risk set for the jth event only after experiencing the preceding $(j-1)$ events. For a composite endpoint, the focus is usually on a single treatment effect across the event-order strata.

The PWP model has two main approaches:

- Total time approach: the time since study start is considered for all events.
- Gap time approach: the time since the previous event is considered.

In our work, we focus on the total time approach to ensure comparability with, for example, the AG model. The hazard for the composite endpoint for event-order stratum j is

$$\lambda_{CE,ij}(t) = \lambda_{CE,0j}(t)\exp(\beta_{j,CE}X_i), \qquad j = 1,\dots,J_i, \tag{6}$$

where $\lambda_{CE,0j}(t)$ is the stratum-specific baseline hazard for the control group and $\beta_{j,CE}$ is the stratum-specific log hazard-ratio parameter for the jth composite-endpoint event [1].

Under the assumption of a common treatment effect across event-order strata,

$$\beta_{1,CE} = \beta_{2,CE} = \cdots = \beta_{J,CE} = \beta_{CE}, \tag{7}$$

the treatment-to-control hazard ratio is

$$HR = \frac{\lambda_{CE,0j}(t)\exp(\beta_{CE})}{\lambda_{CE,0j}(t)} = \exp(\beta_{CE}). \tag{8}$$

Estimation The PWP model estimates the all-cause hazard ratio for the composite endpoint (including all endpoints of interest) while accounting for independent right-censoring. It uses a partial likelihood–based method for effect estimation. In this framework, risk sets are defined separately for each stratum, reflecting the event order of each individual.

Interpretation In the PWP model, the treatment effect is described by the HR, interpreted in the same way as in the AG model. However, note that the HR is derived

through stratum-specific estimation. A corresponding two-sided hypothesis test can be formulated analogously to the AG model.

Assumptions For the PWP model, proportional stratum-specific baseline hazards are assumed. The model assumes the same clinical relevance for all components combined in the composite endpoint. When endpoints are not differentiated, equal cause-specific baseline hazards are assumed; this assumption can be relaxed if stratum-specific estimates are modeled. Compared to the Andersen-Gill model, the Markov assumption is less restrictive here, since the future event process may depend on the number of previous events. It is generally assumed that the treatment effect is constant across all components.

(Stratified) Parametric model

Model Specification For the parametric approach, we assume an accelerated failure time (AFT) model [3, 4]. The event-time distribution is specified parametrically, for example using a Weibull or log-normal distribution. These distributions are characterized by distribution-specific location, scale and/or shape parameters. The treatment effect is expressed as an acceleration factor (AF), representing a "stretching" or "contracting" of the event-time scale (e.g. the probability for a dog to survive 10 years equals the probability for a human to survive 70 years).

For the unstratified approach, the model can be written as

$$\log(T_{ij}) = \alpha_{CE} + \beta_{CE} X_i + \sigma_{CE}\epsilon_{ij}, \tag{9}$$

where T_{ij} is the observed time of the jth composite-endpoint event for individual i, α_{CE} is the baseline location parameter, σ_{CE} is the scale parameter, ϵ_{ij} is an error term whose distribution determines the parametric form of the event-time distribution, and β_{CE} is the treatment-effect coefficient on the log-time scale. The corresponding acceleration factor is

$$AF = \exp(\beta_{CE}). \tag{10}$$

For the stratified approach, the event-order strata are defined as in the PWP model and the baseline distribution parameters are estimated separately for each stratum. The model can be written as

$$\log(T_{ij}) = \alpha_{CE,j} + \beta_{CE,j} X_i + \sigma_{CE,j}\epsilon_{ij}. \tag{11}$$

Here, $\alpha_{CE,j}$ and $\sigma_{CE,j}$ denote the stratum-specific location and scale parameters, respectively, and $\beta_{CE,j}$ denotes the treatment-effect coefficient for event-order stratum j. In our analysis, a common treatment effect across strata is assumed according to Equation (7), so that the common acceleration factor is Equation (10).

Estimation The AFT model estimates the (stratified) all-cause AF for the composite endpoint (including all endpoints of interest) while accounting for independent right-censoring. Parameters within the AFT model are estimated by maximizing the full log-likelihood. Each observed event contributes to the overall likelihood.

Interpretation The AF is interpreted as follows:

- AF > 1: for treatment vs. control, patients in the treatment group are longer without an event compared to control (with that factor)
- AF $= 1$: no difference in time to events
- AF < 1: for treatment vs. control, patients in the treatment group experience events earlier compared to control (with that factor)

A corresponding two-sided hypothesis test can be formulated as:

$$H_0 : \text{AF} = 1 \quad \text{vs.} \quad H_1 : \text{AF} \neq 1$$

Assumptions A specific underlying event time distribution is assumed. For composite endpoints, it is assumed that all event types have the same clinical relevance, and that the treatment effect is identical across all components.

Wei-Lin-Weissfeld (WLW) Model

Model Specification As in the Cox model, the Wei-Lin-Weissfeld (WLW) model combines baseline hazards with treatment-effect coefficients, whose exponentiated values correspond to HR.

In the WLW model, baseline hazards are stratum-specific, and stratum-specific treatment effects can also be estimated. The hazard for the jth composite-endpoint event is specified as in the PWP model. In contrast to the PWP model, an individual is considered at risk in every event-order stratum while under observation, irrespective of whether the preceding event has occurred. If the number of observed events for an individual, J_i, is smaller than the maximal number of events considered, J, artificial censored event times are defined for the remaining strata, with the corresponding event indicator set to zero.

The focus is typically on an overall hazard ratio across the entire event process, i.e., for the composite endpoint. In our application, the combined average regression coefficient is obtained as the arithmetic mean of the stratum-specific coefficients, $\beta_{CE} = \frac{1}{J}\sum_{j=1}^{J} \beta_{j,CE}$ [1]. The corresponding overall treatment effect is

$$HR = \exp(\beta_{CE}). \tag{12}$$

Estimation The WLW model estimates the all-cause hazard ratio for the composite endpoint (including all endpoints of interest) while accounting for independent right-censoring.

In this model, stratum-specific risk sets are defined. If an individual does not reach the maximum number of events observed in the dataset, artificial observations are added for that individual — repeating the last observed time and treating it as censored.

Interpretation In the WLW model, the treatment effect is expressed as a HR, interpreted in the same way as in the AG model. However, note that in the WLW model the HR is obtained through stratum-specific estimation and then averaged across strata. A corresponding two-sided hypothesis test can be formulated as described for the AG and PWP models.

Assumptions The WLW model assumes proportional stratum-specific baseline hazards. It does not assume that the occurrence of a subsequent event is influenced by previous events — that is, the Markov assumption also applies here. It is even less intuitive because the model assumes that one is at risk for all events, i.e. although no second event was observed, one is at risk for a third event. For composite endpoints, it is assumed that all event types have the same clinical relevance, and that the treatment effect is identical across all components.

Negative binomial (NB) model

Model Specification In the negative binomial model, the dependent variable is the observed event count, including all recurrent and fatal events of primary interest, which is assumed to follow a negative binomial distribution. Assume that Y_i is the number of events for individual i and that each individual has a constant event rate. Individual-specific rates are allowed to vary around the treatment-group-specific mean according to a gamma distribution. The resulting marginal distribution of the total event count is a negative binomial distribution.

The expected event count for individual i is

$$\mu_i = \exp\{\beta_0 + \beta_{CE} X_i + \log(\tau_i)\}\,, \tag{13}$$

where β_0 is the intercept, β_{CE} is the log incidence-rate ratio comparing the intervention with the control group, and τ_i is the individual follow-up time included as an offset [5].

The corresponding treatment-to-control incidence-rate ratio (IRR) is

$$IRR = \exp(\beta_{CE}). \tag{14}$$

Estimation The negative binomial model estimates the incidence rate ratio (IRR), which quantifies the relative difference in event rates between groups. The individual observation time can be incorporated as an offset in the model to account for varying exposure times across individuals. Parameters are typically estimated via maximum likelihood estimation.

Interpretation The IRR is interpreted as follows when comparing the intervention group with the control group:

- IRR $>$ 1: the event count rate (for all event types) is increased in the intervention group.

- IRR = 1: no difference in event count rates between groups.
- IRR < 1: the event count rate (for all event types) is decreased in the intervention group.

Corresponding hypothesis tests can be formulated analogously to those used in semi-parametric models.

Assumptions The negative binomial model assumes independence of individual observations and that covariates have multiplicative effects on the event rate. In addition, the model presumes a constant individual-specific incidence rate over time, implying that the underlying event-generating process remains stable during the observation period.

3.1.2 Methods that consider the clinical relevance of endpoints

Weighted Lin-Wei-Ying-Yang (WLWYY) Model

Model Specification Weighted Lin-Wei-Ying-Yang (WLWYY) model builds on the proportional rates/means framework of the LWYY model. The component-specific weights define a weighted composite event process, and the treatment effect acts multiplicatively on the marginal rate of this weighted process. The dependence structure among recurrent and terminal events is left completely unspecified, so no parametric model for their dependence is required.

Let

$$N^{w*}(t) = \sum_{k=1}^{K} w_k^{\text{lwyy}} N_k^*(t), \tag{15}$$

where $N_k^*(t)$ is the cumulative number of events of component k experienced by time t, and w_k^{lwyy} is the corresponding prespecified component-specific weight.

The proportional rates model for the weighted composite endpoint is

$$E\{dN^{w*}(t) \mid X_i\} = d\mu_{CE,0}^{w}(t) \exp(\beta_{CE}^{w} X_i) \tag{16}$$

where $\mu_{CE,0}^{w}(t)$ is the baseline mean function of the weighted composite event process and β_{CE}^{w} is the parameter for defining the difference between treatment and control [6]. Since the treatment indicator X_i is time-invariant, the corresponding proportional means model is

$$\mu_{CE}^{w}(t \mid X_i) = E\{N^{w*}(t) \mid X_i\} = \mu_{CE,0}^{w}(t) \exp(\beta_{CE}^{w} X_i). \tag{17}$$

The corresponding treatment-to-control weighted rate ratio is

$$RR^{w} = \frac{\mu_{CE}^{w}(t \mid X = 1)}{\mu_{CE}^{w}(t \mid X = 0)} = \exp(\beta_{CE}^{w}). \tag{18}$$

Estimation The model proposed by Mao [7] provides an estimator for the weighted

rate or mean ratio, extending the LWYY framework. It incorporates the clinical relevance of combined endpoints via weights applied to the event rates, thereby accounting for the relative importance of different outcomes in the composite measure.

Interpretation The resulting rate ratio can be interpreted similarly to the standard LWYY rate ratio. However, the choice of weights can substantially affect the statistical power and interpretation of the results. For example, if the treatment effect on the terminal event is similar to or smaller than that on recurrent events, giving more weight to the terminal event may reduce statistical power. Hypothesis tests can be formulated as in the LWYY model, but based on the weighted treatment effect.

Assumptions The assumptions of the Weighted LWYY model are similar to those of the standard LWYY model, with the additional consideration that the clinical relevance of endpoints is incorporated through the assigned weights. This includes proportional rates for covariates and independence of individual observations while the weighted composite accounts for differences in clinical importance across endpoints.

(Stratified) Weighted all-cause hazard ratio (WHR)

Model specification Within this approach, weights reflecting clinical relevance are assigned to the cause-specific hazards of the components. Analogously to the unweighted all-cause hazard ratio commonly used for the analysis of composite endpoints, the weighted cause-specific hazards are used to calculate the treatment effect, i.e., the weighted all-cause hazard ratio. A corresponding confidence interval is obtained via bootstrap sampling.

For the (stratified) parametric approach, the cause-specific hazards are estimated using a prespecified parametric survival model. For the non-parametric approach, cause-specific cumulative hazards are used, and a corresponding (stratified) weight-based log-rank test has been described. The prespecified non-negative relevance weights can be chosen as proposed by Ozga et al. [8], where a weight of 1 is assigned to the most clinically relevant event. The unstratified weighted all-cause hazard is

$$\lambda_{CE}^{w}(t \mid X_i = x) = \sum_{k=1}^{K} w_k^{\mathrm{WHR}} \lambda_k(t \mid X_i = x), \tag{19}$$

where $w_k^{\mathrm{WHR}} \geq 0$ is the prespecified clinical relevance weight for component k, and $\lambda_k(t \mid X_i = x)$ is the corresponding cause-specific hazard. The WHR is then

$$HR^{w}(t) = \frac{\lambda_{CE}^{w}(t \mid X_i = 1)}{\lambda_{CE}^{w}(t \mid X_i = 0)} = \frac{\sum_{k=1}^{K} w_k^{\mathrm{WHR}} \lambda_k(t \mid X_i = 1)}{\sum_{k=1}^{K} w_k^{\mathrm{WHR}} \lambda_k(t \mid X_i = 0)}. \tag{20}$$

Analogously, the stratified WHR can be defined:

$$HR_{\mathrm{strat}}^{w}(t) = \frac{1}{J} \sum_{j=1}^{J} \frac{\sum_{k=1}^{K} w_k^{\mathrm{WHR}} \lambda_{k,j}(t \mid X_i = 1)}{\sum_{k=1}^{K} w_k^{\mathrm{WHR}} \lambda_{k,j}(t \mid X_i = 0)}. \tag{21}$$

Estimation In the (stratified) parametric approach, the cause-specific hazards are estimated via an assumed underlying parametric distribution. The Nelson-Aalen estimator can be used to gain the (strata) and cause- and group-specific cumulative hazards. The predefined weights are multiplied to those and these weighted cause-specific (cumulative) hazards are summed up. The ratio of the group-specific weighted (cumulative) hazards describes the treatment effect.

Interpretation The weighted all-cause hazard ratio can be interpreted as the weighted average of cause-specific hazards/hazard ratios. Test-hypotheses for the weighted all-cause hazard ratio can be formulated analogously as described for the semi-parametric unweighted methods.

Assumptions The assumption for the non-parametric estimate is that equal baseline hazards for the different event types are given. However, it was recently shown that the non-parametric estimator is robust against deviations from the equal baseline-hazards assumption. The parametric approach does not need equal cause-specific baseline hazards but assumes a specific underlying event time distribution.

(Stratified) Wei-Lachin (WL) Approach

Model Specification The Wei-Lachin approach estimates cause-specific treatment effects separately for each endpoint. A Cox proportional hazards model is used for fatal events, while the AG or PWP (=stratified Wei-Lachin) model is applied for recurrent events. For each component, prespecified positive relative weights are assigned, which sum to 1. The weights are applied to the component-specific logarithmized (log) hazard ratios and are used to construct a weighted composite treatment effect across all components. For the unstratified Wei-Lachin approach, the component-specific hazards are

$$\lambda_k(t \mid X_i) = \lambda_{k,0}(t) \exp(\beta_k X_i), \tag{22}$$

where $\lambda_{k,0}(t)$ is the baseline hazard for component k and β_k is the corresponding component-specific log hazard-ratio parameter [9].

For the stratified approach, the recurrent-event hazards are stratified by event order and can be written as

$$\lambda_{k,j}(t \mid X_i) = \lambda_{k,0j}(t) \exp(\beta_k X_i), \tag{23}$$

where $\lambda_{k,0j}(t)$ is the stratum-specific baseline hazard for component k. The component-specific treatment effect β_k is assumed to be common across event-order strata.

For both approaches, the component-specific log hazard ratios are combined using the prespecified Wei-Lachin weights. The corresponding weighted treatment effect is

$$HR_{\mathrm{WL}} = \exp\left(\sum_{k=1}^{K} w_k^{WL} \beta_k\right), \tag{24}$$

where w_k^{WL} denotes the prespecified relevance weight for component k.

Estimation The Cox, AG, or PWP models are employed to estimate the weighted hazard ratio according to the Wei-Lachin method, following the procedures described in previous sections.

Interpretation The Wei-Lachin approach produces a weighted composite hazard ratio, derived by combining the weighted cause-specific log-hazard ratios. The influence of each event type is proportional to its weighted log-hazard ratio. This means that an event type with a higher log-hazard ratio has a stronger impact on the composite effect, which can increase variability if the number of events is small.

Interpretation of the resulting HR follows the same principles as in the Cox-based models. Hypothesis tests can be formulated analogously, but are based on the weighted effect estimate.

Assumptions The assumptions underlying the Wei-Lachin approach correspond to those of the Cox, AG, or PWP models used for estimating the cause-specific effects. This includes proportional hazards, independence assumptions for recurrent events (or stratification for PWP), and assumptions about the baseline hazard or rate structure, depending on the chosen model for each event type.

Approach by Bakal et al.

Model specification / Estimation / Interpretation Problematic for this approach is that no underlying model is defined and as a consequence, the estimand is unspecified. By this, there naturally also is no effect estimator. The approach is based on so-called "weighted survival functions"; however, the weighting scheme is only described at the estimation level. Therefore, the formulation of formal test hypotheses is not possible. An effect estimate cannot be gained via this approach but only a test decision can be made whether two or more of the weighted Kaplan-Meier curves are equal. The predefined non-negative relevance weights within this approach work recursively on the observed event counts, where the recursion is with respect to all previous events for an individual and hence produces a weighted individual score for event count and risk set.

Assumptions For the estimation, it is assumed that there are no differences between strata, i.e. no risk change for subsequent events. Furthermore, individuals are at risk as long as they are under observation but their contribution to the event number and number at risk changes for subsequent events.

Win ratio (WR) and win odds (WO)

Model Specification The win ratio (WR) and win odds (WO) approaches compare each patient in the treatment group with each patient in the control group with respect to the endpoints of interest. For example, for time-to-event endpoints, the event or censoring times are compared and it is counted how often the treatment-group patients survive longer, i.e. "wins" over the control-group patient. Analogously, a "loss" occurs if the control-group patients survive longer. A tie can occur if the two patients have the same survival time. Thus, for each patient pair, a win, loss, or tie is assigned. In the end, the proportions of wins, losses, and ties are evaluated, and the proportion of winners in the treatment and control groups is compared. Comparisons may sometimes be impossible due to censoring before an event occurs; such cases are ignored in the WR/WO analysis [10].

Let W, L, and T denote the numbers of treatment wins, treatment losses, and ties, respectively. The win ratio is

$$WR = \frac{W}{L}. \tag{25}$$

Equivalently, at the population level, let p_W, p_L, and p_T denote the probabilities of a treatment win, treatment loss, and tie, respectively. The WR estimand is $\frac{p_W}{p_L}$.

In contrast to the WR, the WO incorporates ties by assigning half of each tie to both the treatment-win and treatment-loss quantities. The win odds is

$$WO = \frac{W + \frac{1}{2}T}{L + \frac{1}{2}T}, \tag{26}$$

with corresponding population estimand is $\frac{p_W + \frac{1}{2}p_T}{p_L + \frac{1}{2}p_T}$.

Estimation The win ratio or win odds estimates the total number of wins in the treatment group divided by the number of losses (or wins in the control group), representing the proportion of wins. The win ratio ignores ties and thus only includes the subpopulation of comparable observations. When many ties exist, the win odds is recommended, as it incorporates ties into the analysis. Estimation can be performed using standard non-parametric approaches, with confidence intervals derived from the comparable pairs.

Interpretation The WR or WO is interpreted as follows when comparing treatment versus control:

- WR or WO > 1: more wins in the treatment group.
- WR or WO $= 1$: no difference in win proportions between groups.
- WR or WO < 1: fewer wins in the treatment group.

For example, a WR or WO of 1.25 indicates that the proportional win rate is 25% higher in the treatment group (for WR, this applies to the subset of patients whose outcomes can be classified as discrete wins or losses, ignoring ties).

To compare WR/WO with hazard ratios, the reciprocal (1/WR) can be used. Note that WR/WO does not indicate when an effect occurs, nor its durability or magnitude. The overall effect size cannot be directly interpreted from the WR alone, and understanding the population-level effect requires examining all paired comparisons, including tied outcomes.

Hypothesis tests for the inverted WR/WO can be formulated analogously to the methods described above.

Assumptions The win ratio and win odds approaches assume that the treatment effects are equal across the combined endpoints and that the endpoints are independent.

3.1.2.1 Generalized Pairwise Comparisons of Prioritized Outcomes

Model Specification As in the WR/WO analysis, generalized pairwise comparisons (GPC) compare each patient in the treatment group with each patient in the control group with respect to the prioritized outcomes of interest. Each comparison is classified as favorable for treatment, unfavorable, neutral, or uninformative (e.g., due to missing observations or censoring). The concept of a favorable comparison is analogous to a "win" in the win ratio approach. For a time-to-event outcome, a longer event-free time in the treatment group is favorable, whereas a shorter time is unfavorable. For an event-count outcome, a smaller number of events in the treatment group is favorable, a larger number is unfavorable, and equal counts are neutral.

Let $\ell = 1, \ldots, L$ index the prioritized outcome measures used in the GPC hierarchy, such as a time-to-event outcome or an event count. These outcome measures may be derived from the K components of the composite endpoint, and more than one outcome measure may be defined for the same component [11].

For a treatment–control pair, let i and i' denote two individuals such that $X_i = 1$ and $X_{i'} = 0$, respectively. The pairwise scoring indicator for outcome measure ℓ is defined as

$$s_{ii'}(\ell) = \begin{cases} 1, & \text{if the comparison is favorable for treatment,} \\ -1, & \text{if the comparison is unfavorable for treatment,} \\ 0, & \text{if the comparison is neutral.} \end{cases} \tag{27}$$

An uninformative comparison cannot be classified as favorable, unfavorable, or neutral on the basis of the available information. According to the prespecified hierarchy, lower-priority outcomes are considered only if the comparison at all higher-priority outcomes is neutral or uninformative; otherwise, the lower-priority outcomes are ignored for that pair.

At priority level ℓ, let $\delta(\ell)$ denote the level-specific contribution to the net chance of a better outcome. It is defined as the probability of a favorable comparison at that priority level minus the probability of an unfavorable comparison at that priority level.

For an event-count outcome, for which smaller values are preferable, this can be written as

$$\delta(\ell) = P\{\text{pair reaches level } \ell,\, Y_{i\ell} < Y_{i'\ell}\} - P\{\text{pair reaches level } \ell,\, Y_{i\ell} > Y_{i'\ell}\}\,, \quad (28)$$

where $Y_{i\ell}$ and $Y_{i'\ell}$ denote outcome measure ℓ for a randomly selected treatment individual i and control individual i', respectively. A pair reaches level ℓ if all higher-priority outcomes for that pair are neutral or uninformative. The cumulative net chance of a better outcome up to priority level ℓ is

$$\Delta(\ell) = \sum_{z=1}^{\ell} \delta(z). \quad (29)$$

The cumulative net chance at the lowest-priority outcome, $\Delta(L)$, is the overall proportion in favor of treatment (PiF), also referred to as the overall net chance of a better outcome:

$$\text{PiF} = \Delta(L) = \sum_{z=1}^{L} \delta(z). \quad (30)$$

For a time-to-event outcome measure ℓ, let $Y_{i\ell}$ and $Y_{i'\ell}$ denote the underlying event times for treatment individual i and control individual i', respectively, and let $U_{i\ell}$ and $U_{i'\ell}$ denote the corresponding censoring times. The observed times are

$$\widetilde{Y}_{i\ell} = \min(Y_{i\ell}, U_{i\ell}), \qquad \widetilde{Y}_{i'\ell} = \min(Y_{i'\ell}, U_{i'\ell}). \quad (31)$$

The corresponding event indicators are

$$d_{i\ell} = I(Y_{i\ell} \leq U_{i\ell}), \qquad d_{i'\ell} = I(Y_{i'\ell} \leq U_{i'\ell}), \quad (32)$$

where a value of 1 indicates an observed event and a value of 0 indicates right censoring. If a clinically relevant difference is prespecified for outcome measure ℓ, let $\eta_\ell \geq 0$ denote the corresponding threshold. If no such threshold is used, $\eta_\ell = 0$. The possible pairwise classifications under the standard GPC procedure are summarized in Table 1, adapted from Buyse [12].

Estimation The primary measure of treatment effect is the proportion in favor of treatment (PiF), corresponding to $\Delta(L)$ defined above. This is estimated as the net difference between the number of favorable and unfavorable pairs divided by the total number of treatment-control pairs. Randomization tests can be used to test the null hypothesis that the proportion in favor of treatment equals zero (no difference between groups), and to construct confidence intervals for PiF.

Interpretation For each endpoint, PiF indicates how often the treatment yields a better outcome compared to the control:

- $PiF < 0$: proportion in favor of treatment is lower than control (treatment worse)
- $PiF = 0$: no difference between groups
- $PiF > 0$: proportion in favor of treatment is higher than control (treatment better)

Table 1: Standard generalized pairwise comparisons for a time-to-event outcome in the presence of right censoring.

Censoring status	Pairwise comparison	Pair is
$d_{i\ell} = 1,\ d_{i'\ell} = 1$	$\widetilde{Y}_{i\ell} - \widetilde{Y}_{i'\ell} > \eta_\ell$	Favorable
	$\left\lvert \widetilde{Y}_{i\ell} - \widetilde{Y}_{i'\ell} \right\rvert \leq \eta_\ell$	Neutral
	$\widetilde{Y}_{i\ell} - \widetilde{Y}_{i'\ell} < -\eta_\ell$	Unfavorable
$d_{i\ell} = 0,\ d_{i'\ell} = 1$	$\widetilde{Y}_{i\ell} - \widetilde{Y}_{i'\ell} > \eta_\ell$	Favorable
	$\left\lvert \widetilde{Y}_{i\ell} - \widetilde{Y}_{i'\ell} \right\rvert \leq \eta_\ell$	Uninformative
	$\widetilde{Y}_{i\ell} - \widetilde{Y}_{i'\ell} < -\eta_\ell$	Uninformative
$d_{i\ell} = 1,\ d_{i'\ell} = 0$	$\widetilde{Y}_{i\ell} - \widetilde{Y}_{i'\ell} > \eta_\ell$	Uninformative
	$\left\lvert \widetilde{Y}_{i\ell} - \widetilde{Y}_{i'\ell} \right\rvert \leq \eta_\ell$	Uninformative
	$\widetilde{Y}_{i\ell} - \widetilde{Y}_{i'\ell} < -\eta_\ell$	Unfavorable
$d_{i\ell} = 0,\ d_{i'\ell} = 0$	Any observed time difference	Uninformative

The cumulative net difference across prioritized endpoints provides an overall measure of treatment benefit. Interpretation focuses on the relative frequency of favorable outcomes rather than direct hazard ratios or rates.

Assumptions The methods assume a combined effect for all endpoints (net effect).

3.2 Composite endpoint considering correlated competing events or no composite endpoints

(Semi-)parametric joint frailty (JF) model

Model Specification In the semi-parametric joint frailty model, the hazard for experiencing an event is decomposed into a combination of a baseline hazard, which describes the underlying event rate for all individuals, and a treatment effect, expressed as a hazard ratio. Unlike the simpler models described earlier, the joint frailty model specifies two hazard functions—one for the recurrent event process and another for the terminal (fatal) event—resulting in two distinct baseline hazards and two corresponding hazard ratios. The two hazards can also be modeled assuming a parametric distribution. These hazards are correlated through random effects (frailties), which capture the dependence between recurrent and terminal events. For example, a patient experiencing frequent recurrent events may also have an increased (or decreased) risk of a terminal event. In the semi-parametric formulation, the baseline hazards are treated as nuisance functions, and the focus lies on estimating the hazard ratios while allowing the baseline hazards to vary flexibly over time. The two processes in the joint frailty model can be written as

$$\lambda_i^R(t \mid v_i) = v_i \lambda_0^R(t) \exp\left(\beta^R X_i\right), \tag{33}$$

and

$$\lambda_i^T(t \mid v_i) = v_i^{\alpha} \lambda_0^T(t) \exp\left(\beta^T X_i\right). \tag{34}$$

Here, $\lambda_i^R(t \mid v_i)$ denotes the intensity of the event process entered into the recurrent-model part, and $\lambda_i^T(t \mid v_i)$ denotes the terminal-event hazard for individual i, conditional on the shared frailty v_i. The functions $\lambda_0^R(t)$ and $\lambda_0^T(t)$ are the corresponding baseline functions, and β^R and β^T are the treatment-effect parameters for the recurrent-model and terminal-event processes, respectively. The parameter α allows the shared frailty to have a different effect on the terminal-event process than on the recurrent-event process. Individual i may experience multiple events in the recurrent-model process, but these events arise from the same subject-specific intensity process $\lambda_i^R(t \mid v_i)$.

The frailty terms are assumed to be independently and identically distributed as

$$v_i \sim \text{Gamma}\left(\text{shape} = \frac{1}{\theta}, \text{rate} = \frac{1}{\theta}\right), \tag{35}$$

such that $E(v_i) = 1, \text{Var}(v_i) = \theta$.

The parameter θ represents the variance of the shared frailty and therefore quantifies unexplained between-individual heterogeneity. The parameter α determines how the shared frailty enters the terminal-event hazard and thereby contributes to the association between the recurrent-model and terminal-event processes.

Conditional on the shared frailty, the treatment effects for the two processes are

$$HR^R = \exp(\beta^R), \qquad HR^T = \exp(\beta^T), \tag{36}$$

where HR^R denotes the treatment-to-control hazard ratio for the recurrent-model process and HR^T the treatment-to-control hazard ratio for the terminal-event process [13].

Depending on the endpoint definition, two joint-model specifications can be considered. In the first specification, the recurrent-model process contains the recurrent non-fatal component(s) of the composite endpoint, whereas the fatal component of the composite endpoint is modeled as the terminal event. A separate competing fatal event is treated as independent right censoring.

In the second specification, the recurrent-model process represents the complete composite endpoint, including the recurrent non-fatal component(s) and one fatal component counted once. A separate competing fatal event is then modeled as the terminal event.

Estimation The joint frailty model estimates two treatment effects (HRs or AFs) while accounting for the correlation between the recurrent and terminal event processes and for independent right-censoring. Estimation is typically performed using penalized likelihood methods, where each observed event contributes to the overall likelihood. The risk set at each event time includes all individuals who remain under observation. The inclusion of frailty terms enables modeling of subject-specific heterogeneity

and correlation between event processes, providing a more realistic representation of clinical data where event risks are not independent.

Interpretation The resulting HRs/AFs are interpreted similarly to those from the Cox, AG, or parametric models, but with the distinction that they are estimated under correlated hazards. Hypothesis testing is also analogous, with the null hypothesis formulated for each treatment effect, but accounting for the correlation between event types.

Assumptions The semi-parametric joint frailty model assumes that recurrent and terminal events cannot occur simultaneously and that the proportional hazards assumption holds conditionally on the frailty terms. In the parametric joint frailty model, the PH assumption is relaxed. The same clinical relevance/treatment effect of the combined outcomes is assumed. It further assumes that the frailty distribution (often gamma-distributed) adequately captures unobserved heterogeneity and that the recurrent and terminal event processes share a common random effect that induces dependence between them. In a composite endpoint, the same clinical relevance for endpoints is assumed.

Multi-state models (MSM)

Model Specification In a multi-state model, states for events are described, and a treatment effect is estimated for each transition between those event states. Thereby, the already described semi-parametric Cox-based models as well as the parametric models can be used to describe the hazards between event transitions (e.g the AG and PWP models are also a specific formulation of a multi-state model).

Estimation One can use a semi-parametric Cox-based approach to gain an HR for the treatment comparison for the transitions between events (states). A parametric approach could be applied as well.

Interpretation For all defined transitions, effect estimates in terms of HRs (or AFs) can be obtained, which can be interpreted as already described for other models with HRs (or AFs).

Assumptions Often the process is assumed to be Markovian. If semi-parametric Cox-based models are used to estimate treatment effects for transitions, the PH assumption applies.

Ghosh and Lin (GL) Model

Model Specification The Ghosh and Lin model is a marginal model for recurrent events that accounts for death as a terminal event. The recurrent-event process is modeled marginally while accounting for the fact that the terminal event precludes

further recurrent events. Covariates are assumed to have multiplicative effects on the marginal mean and rate functions of recurrent events [14].

Let $N^R(t)$ denote the cumulative number of recurrent events up to time t, which does not increase after the terminal event, and define the marginal mean function as

$$\mu_R(t \mid X_i) = E\{N^R(t) \mid X_i\}. \tag{37}$$

For the time-invariant treatment indicator X_i, the proportional means model is

$$\mu_R(t \mid X_i) = \mu_{R,0}(t) \exp(\beta_R X_i), \tag{38}$$

where $\mu_{R,0}(t)$ is the unspecified baseline mean cumulative event function and β_R is the treatment-effect coefficient for the recurrent-event process.

For the two-group comparison, the treatment-to-control mean ratio is

$$RR = \frac{\mu_R(t \mid X_i = 1)}{\mu_R(t \mid X_i = 0)} = \exp(\beta_R). \tag{39}$$

Estimation The Ghosh and Lin model estimates cause-specific rate ratios. In the estimation procedure, inverse probability of censoring or survival weighting is used to account for terminal events. This approach ensures that the dependence between recurrent events and terminal events is properly addressed.

Interpretation The estimated rate ratios can be interpreted similarly to hazard ratios. Under the proportional rates model, the cause-specific rate function is analogous to a cause-specific hazard function in Cox models. Hypothesis tests can be formulated as in the Andersen-Gill model, with the difference that the tests are applied to separate treatment effects for each event type.

Assumptions The Ghosh and Lin model assumes proportional means or rates, which is conceptually similar to the proportional hazards assumption in Cox models. The same clinical relevance/treatment effect of the combined outcomes is assumed.

Area Under the Curve (AUC)

Model Specification The AUC approach summarizes cumulative event burden up to a prespecified fixed time point τ. The cumulative event experience is represented by the mean cumulative count curve, which describes the mean number of composite-endpoint events experienced per patient over time, while appropriately adjusted for loss to follow-up and terminal events that preclude subsequent events [15].

For treatment group $X_i = x$, with $x \in \{0, 1\}$, let $\mu_{CE}(t \mid X_i = x) = E\{N_{CE}(t) \mid X_i = x\}$ denote the mean cumulative number of composite-endpoint events by time t. The AUC up to time τ is defined as

$$A_x(\tau) = \int_0^\tau \mu_{CE}(t \mid X_i = x)\, dt. \tag{40}$$

The treatment-to-control AUC ratio is

$$\theta_{\text{AUC,ratio}}(\tau) = \frac{A_1(\tau)}{A_0(\tau)}. \tag{41}$$

The treatment-control AUC difference is

$$\theta_{\text{AUC,diff}}(\tau) = A_1(\tau) - A_0(\tau). \tag{42}$$

Estimation The AUC estimates the mean cumulative count of events over time. Group-specific AUCs can be compared using the AUC ratio or the AUC difference.

In the presence of censoring, the mean cumulative count curve can be estimated using the Nelson-Aalen estimator, provided there are no competing terminal events. If competing events are observed, the non-parametric estimator by Ghosh and Lin can be used to estimate the mean cumulative count curve.

Interpretation The AUC reflects the mean total event-free time lost due to multiple undesirable outcomes. If a patient experiences numerous recurrent events during follow-up, their total event-free time lost can exceed the duration of follow-up.

- AUC difference > 0 or AUC ratio > 1: event-time is increased for the intervention group.
- AUC difference $= 0$ or AUC ratio $= 1$: event-time is equal between groups.
- AUC difference < 0 or AUC ratio < 1: event-time is decreased for the intervention group, meaning that on average, patients spent less time under increased disease burden, and the corresponding total event-free time lost was reduced relative to control.

Hypothesis tests for the AUC ratio or AUC difference can be formulated analogously to semi-parametric methods, with null hypotheses such as AUC difference $=$ 0.

Assumptions The same clinical relevance/treatment effect of the combined outcomes is assumed.

'While-Alive' (WA) Strategy

Model specification The while-alive strategy considers recurrent events in the presence of death as a terminal event. Let D_i denote the terminal-event time for individual i; this may also be the time to a competing terminal event. Let $N_{D,i}(t) = I(D_i \leq t)$ denote the corresponding terminal-event counting process. For each recurrent non-fatal component k of the composite endpoint, let $N_{k,i}(t)$ denote the corresponding counting process. Since no recurrent events can occur after the terminal event, $N_{k,i}(t) = N_{k,i}(t \wedge D_i)$, where $t \wedge D_i = \min(t, D_i)$ [16].

The event history up to time t is denoted by

$$\mathcal{H}_i(t) = \{N_{D,i}(u), N_{1,i}(u), \ldots, N_{K,i}(u) : 0 \leq u \leq t\}. \tag{43}$$

For treatment group $X_i = x$, with $x \in \{0, 1\}$, the while-alive event rate for recurrent event type k over the interval $[0, \tau]$ is

$$r_{k,x}^{WA}(\tau) = \frac{E\{N_{k,i}(\tau) \mid X_i = x\}}{E(D_i \wedge \tau \mid X_i = x)}, \tag{44}$$

where the numerator is the expected cumulative number of recurrent events and the denominator is the restricted mean survival time (RMST).

More generally, Mao [17] extends the while-alive event rate by replacing the numerator with a generic loss function $\mathcal{L}(\mathcal{H}_i)(\cdot)$. Let $\mathcal{L}(\mathcal{H}_i)(dt) \geq 0$ denote the instantaneous loss incurred at time t. The loss function is required to satisfy

$$\mathcal{L}(\mathcal{H}_i)(dt) = 0 \quad \text{for all } t > D_i,$$

and

$$\mathcal{L}(\mathcal{H}_i)(dt) \text{ is a function only of } \mathcal{H}_i(t).$$

Thus, loss can only be accrued while the individual is alive and may depend on the present and past event history, but not on future events. The loss function may also incorporate a terminal event, allowing recurrent and terminal events to contribute jointly to a composite while-alive estimand.

The cumulative loss up to time τ is

$$\mathcal{L}(\mathcal{H}_i)(\tau) = \int_0^\tau \mathcal{L}(\mathcal{H}_i)(dt). \tag{45}$$

For treatment group $X_i = x$, the corresponding while-alive loss rate is

$$l_x(\tau) = \frac{E\{\mathcal{L}(\mathcal{H}_i)(\tau) \mid X_i = x\}}{E(D_i \wedge \tau \mid X_i = x)}. \tag{46}$$

It represents the average loss experienced per unit of time alive over the interval $[0, \tau]$. The same prespecified loss function must be used in both treatment groups to ensure comparability of the group-specific loss rates. The treatment contrast is expressed as the treatment-to-control while-alive loss-rate ratio,

$$RR(\tau) = \frac{l_1(\tau)}{l_0(\tau)}. \tag{47}$$

Estimation To quantify the relationship between recurrent events and death across treatment groups, a non-parametric summary measure can be used (i.e. Kaplan-Meier and Nelson-Aalen estimates can be used). This approach calculates the ratio of the expected number of recurrent events per group, adjusted for overall survival, effectively classifying recurrent event counts in relation to survival time.

Interpretation The resulting rate ratio (RR) can be interpreted as follows:

- RR $>$ 1: the rate of recurrent events is increased in the intervention group.
- RR $=$ 1: no difference in recurrent event rates between groups.
- RR $<$ 1: the rate of recurrent events is decreased in the intervention group.

Estimates should be interpreted in the context of the population and time period studied, and compared with other relevant analyses for a comprehensive understanding.

Assumptions The same clinical relevance/treatment effect of the combined outcomes is assumed.

3.3 Further data description

Model specification Since the described methods are non-parametric methods to describe the data, there is no real model. One could assume an underlying multi-state model and the methods are used to illustrate the transitions between the events (states).

Estimation The non-parametric Nelson-Aalen estimate can be used to estimate the cumulative (cause-specific) hazard or transition hazards for treatment groups.
The non-parametric Aalen-Johansen estimate can be used to estimate the cumulative incidences and transition probabilities for treatment groups and the state-occupation probabilities.

Interpretation The cumulative hazards display the event distribution in terms of accumulated event rates, i.e., a lower cumulative hazard is better. The same applies to the Aalen-Johansen estimate.

Assumptions The event process should be Markovian in case one wants to estimate the cumulative hazards or incidences. The state occupation probabilities are the results of a non-Markovian process. If events are combined for the estimation, the same clinical relevance is assumed for the endpoints.

4 Further approaches

- Cook and Lawless [18] described methods that use marginal event occurrence rates but are either limited in the range of situations they can handle or complicated and depend on assumptions about the failure time distribution that cannot be proved.
- Cheng and Cook [19] described a testing procedure for testing for differences in mean functions in the setting of multivariate recurrent events terminated by a fatal event. The idea is to construct marginal test statistics for each type of recurrent event and thereby adjust for the possibility of dependent termination. Afterwards, the evidence across all event types is combined by constructing a linear global test statistic.
- Frailty models: Wang et al. [20] described a method where the occurrence of recurrent events is modeled by a subject-specific nonstationary Poisson process via a latent variable. Thereby, they consider a multiplicative intensity model as the underlying model for non-parametric estimation of the cumulative rate function and

extend it to a semi-parametric approach. The distributions of the censoring and latent variables are treated as nuisance parameters and therefore not modeled or estimated. Liu et al. [21] described a shared frailty model, thereby assuming that the recurrent events follow a nonhomogeneous Poisson process conditional on the frailty. Thereby, the frailty effect is allowed to be different for the two processes. Huang and Wang [22] described a semi-parametric method for the joint modeling of a recurrent event process and a failure time where a common subject-specific latent variable (frailty) is used to model the association between the intensity of the recurrent event process and the hazard of the failure time. Thereby, the frailty distribution is treated as a nuisance parameter and not modeled parametrically. Ye et al. [23] described a semi-parametric method to jointly model the recurrent and terminal event processes where the dependence is modeled by a shared gamma frailty that is included in both the recurrent event rate and terminal event hazard function. Thereby, marginal models are used to estimate the regression effects on the terminal and recurrent event processes, and a Poisson model is used to estimate the dispersion of the frailty variable. Zeng and Lin [24] described a class of semi-parametric transformation models with shared random effects for the joint analysis of recurrent events and a terminal event. Thereby, they include proportional hazards/intensity and proportional odds models and estimate the model parameters by the non-parametric maximum likelihood approach.

- Additive models: Schaubel et al. [25] described a semi-parametric model for the marginal recurrent event rate, i.e. an additive rates model. Thereby, the treatment effect is assumed to add to an unspecified baseline rate. Hence, absolute effects are estimated, which are of less interest in the study of incidence or mortality. Zeng and Cai [26] described a semi-parametric additive rate model to model recurrent events in the presence of a terminal event with a non-parametric dependence between recurrent events and terminal events. Thereby, the baseline rate function is non-parametric and depends on latent random variables which are associated with the terminal event.
- Bayesian approaches: Osmani et al. [27] proposed a Bayesian approach for the joint frailty model with two recurrent and one fatal event/endpoint. They use the Markov Chain Monte Carlo (MCMC) method to estimate all model parameters. Li et al. [28] model recurrent gap times and a terminal event in a joint frailty model. They used a copula technique to model the correlation between recurrent events and a terminal event and used Metropolis–Hastings within the Gibbs Sampler algorithm for parameter estimation. Paulon et al. [29] also modeled recurrent gap times and a terminal event. Thereby, the frailty is modeled via a flexible Dirichlet process and a MCMC method is used for estimation. Van den Boom [30] extended these approaches to be more flexible (with fewer assumptions).
- Pseudo observations: Furberg et al. [31] described a bivariate marginal model with pseudo-observations, i.e. joint marginal regression models for both recurrence and death are considered and estimation of the parameters in the marginal models is based on pseudo-observations and hence estimates a mean ratio and a hazard ratio parameter for the recurrent event and terminal event process, respectively. The method is implemented in the R package recurrentpseudo [32].

- Restricted mean in favor [33]: The restricted mean time in favor (RMT-IF) of treatment is a non-parametric effect size in a multi-state setting. It is defined as the net average time the treatment group spends in a more favorable state than the control groups over a prespecified time window. It is a model-free method. The method is implemented in the rmt package in R [34].
- Parametric Estimation of the Mean Number of Events in the Presence of Competing Risks [35]: Recently, Entrop et al. introduced a parametric estimator for estimating the mean number of events in the presence of competing risks by jointly modeling the intensity function of the recurrent event and the survival function for the competing events. The method is implemented in the JointFPM package in R [36].

5 Interpretation of estimates in the EAST-AFNET 4 application

Table A1: Application-specific interpretation of the estimates

**Method**	**Estimate in application**	**Interpretation in this application**
**Composite endpoints without clinical weighting**		
AG model	HR = 0.76 (95% CI 0.67, 0.86)	For early rhythm control, a 24% lower hazard for the composite endpoint was observed compared to usual care, i.e. the hazard was 0.76 times that under usual care.
LWYY model	RR = 0.76 (95% CI 0.62, 0.93)	For early rhythm control, a 24% lower event rate of the composite endpoint was observed compared to usual care, i.e. the event rate was 0.76 times that under usual care.
PWP model	HR = 0.85 (95% CI 0.74, 0.97)	Considering the event order, for early rhythm control, a 15% lower hazard for the composite endpoint was observed compared to usual care, i.e. the hazard was 0.85 times that under usual care.
WLW model	HR = 0.75 (95% CI 0.66, 0.85)	For early rhythm control, a 25% lower marginal hazard for the composite endpoint was observed compared to usual care, i.e. the hazard was 0.75 times that under usual care.

Continued on next page

Table A1: Application-specific interpretation of the estimates (continued)

**Method**	**Estimate in application**	**Interpretation in this application**
NB model	IRR = 0.71 (95% CI 0.56, 0.90)	For early rhythm control, 29% fewer events per unit of follow-up (from the composite endpoint) were observed compared to usual care, i.e., the event rate was 0.71 times that under usual care.
Parametric time-to-event model with all events of all endpoints	1/AF = 0.74 (95% CI 0.64, 0.85)	For early rhythm control, composite-endpoint events occurred later than under usual care, i.e. the estimated event times were 1.35 times as long as those under usual care (AF = 1.35).
Stratified parametric time-to-event model with all events of all endpoints	1/AF = 0.79 (95% CI 0.66, 0.94)	Considering the event order, composite-endpoint events occurred later under early rhythm control, i.e. the estimated event times were 1.27 times as long as those under usual care (AF = 1.27).
**Methods considering clinical relevance**		
WLWYY model	Weighted RR = 0.76 (95% CI 0.63, 0.92)	For early rhythm control, a 24% lower weighted event rate of the composite endpoint was observed compared to usual care, i.e. the weighted rate was 0.76 times that under usual care.
Parametric WHR approach	Weighted all-cause HR = 0.81 (95% bootstrap CI 0.61, 1.08)	For early rhythm control, a 19% lower weighted hazard of the composite endpoint was observed compared to usual care, i.e. the weighted hazard was 0.81 times that under usual care.
Non-parametric WHR approach	Weighted all-cause HR = 0.71 (95% bootstrap CI 0.42, 1.03)	For early rhythm control, a 29% lower weighted hazard of the composite endpoint was observed compared to usual care, i.e. the weighted hazard was 0.71 times that under usual care.
WL approach	Weighted HR = 0.76 (95% CI 0.58, 0.93)	For early rhythm control, a 24% lower hazard of the composite endpoint was observed compared to usual care, i.e. the weighted hazard was 0.76 times that under usual care.

Continued on next page

Table A1: Application-specific interpretation of the estimates (continued)

**Method**	**Estimate in application**	**Interpretation in this application**
Stratified WL approach	Weighted HR = 0.82 (95% CI 0.64, 0.99)	Considering the event order, for early rhythm control, an 18% lower hazard of the composite endpoint was observed compared to usual care, i.e. the weighted hazard was 0.82 times that under usual care.
WR	1/WR = 0.76 (95% CI 0.65, 0.90)	For early rhythm control, the proportion of wins (i.e. fewer composite-endpoint events and, when event counts were equal, a later last event) was higher than the proportion of losses compared with usual care; among non-tied comparisons, the probability of a win was 1.32 times the probability of a loss (WR=1.32).
WO	1/WO = 0.91 (95% CI 0.86, 0.96)	For early rhythm control, the proportion of winners (i.e. fewer events and a later last event of the composite endpoint) was higher compared to usual care in the population considering ties, i.e. the probability of a win plus half the probability of a tie was 1.10 times the probability of a loss plus half the probability of a tie (WO=1.10).
**Joint-frailty and event-burden approaches**		
Semi-parametric joint frailty model	HR = 0.74 (95% CI 0.64, 0.87)	For early rhythm control, a 26% lower hazard of the composite endpoint was observed compared to usual care, considering a correlated competing event, i.e. the hazard was 0.74 times that under usual care.
Parametric joint frailty model	HR = 0.74 (95% CI 0.64, 0.87)	For early rhythm control, a 26% lower hazard of the composite endpoint was observed compared to usual care, considering a correlated competing event, i.e. the hazard was 0.74 times that under usual care.
AUC approach	AUC ratio = 0.77 (95% CI 0.62, 0.96)	For early rhythm control, the area under the mean cumulative composite-event count curve was 23% lower than under usual care over the observational period, i.e. the AUC was 0.77 times that under usual care after accounting for censoring.

Continued on next page

Table A1: Application-specific interpretation of the estimates (continued)

**Method**	**Estimate in application**	**Interpretation in this application**
WA estimate	WA loss-RR = 0.79 (95% CI 0.53, 1.19)	For early rhythm control, the average recurrent non-fatal event burden per unit time alive was 21% lower than under usual care over the observational period, i.e. the WA loss rate was 0.79 times that under usual care.

References

[1] Ozga, A.-K., Kieser, M., Rauch, G.: A systematic comparison of recurrent event models for application to composite endpoints. BMC Medical Research Methodology **18**(1), 2 (2018) https://doi.org/10.1186/s12874-017-0462-x

[2] Lin, D.Y., Wei, L.-J., Yang, I., Ying, Z.: Semiparametric regression for the mean and rate functions of recurrent events. Journal of the Royal Statistical Society: Series B (Statistical Methodology) **62**(4), 711–730 (2000) https://doi.org/10.1111/1467-9868.00259

[3] Kleinbaum, D., Klein, M.: Survival Analysis: A Self-Learning Text. 3rd Edition. Springer, New York (2012). https://doi.org/10.1007/978-1-4419-6646-9

[4] Zhang, Z.: Parametric regression model for survival data: Weibull regression model as an example. Annals of Translational Medicine **4**(24) (2016) https://doi.org/10.21037/atm.2016.08.45

[5] Wang, Y.C., Meyerson, L., Tang, Y.Q., Qian, N.: Statistical methods for the analysis of relapse data in MS clinical trials. Journal of the Neurological Sciences **285**(1–2), 206–211 (2009) https://doi.org/10.1016/j.jns.2009.07.017

[6] Mao, L., Lin, D.Y.: Semiparametric regression for the weighted composite endpoint of recurrent and terminal events. Biostatistics **17**(2), 390–403 (2016) https://doi.org/10.1093/biostatistics/kxv050

[7] Mao, L., Lin, Y.: Semiparametric regression for the weighted composite endpoint of recurrent and terminal events. Biostatistics **17**(1), 390–403 (2016) https://doi.org/0.1093/biostatistics/kxv050

[8] Ozga, A., , Rauch, G.: Introducing a new estimator and test for the weighted all-cause hazard ratio. BMC Med Res Methodol **19**(118), 1–16 (2019) https://doi.org/10.1186/s12874-019-0765-1

[9] Ozga, A.-K., Rauch, G.: Weighted composite time to event endpoints with recurrent events: comparison of three analytical approaches. BMC Medical Research Methodology **22**(1), 38 (2022) https://doi.org/10.1186/s12874-022-01511-1

[10] Pocock, S.J., Ariti, C.A., Collier, T.J., Wang, D.: The win ratio: a new approach to the analysis of composite endpoints in clinical trials based on clinical priorities. European Heart Journal **33**(2), 176–182 (2012) https://doi.org/10.1093/eurheartj/ehr352

[11] Péron, J., Buyse, M., Ozenne, B., Roche, L., Roy, P.: An extension of generalized pairwise comparisons for prioritized outcomes in the presence of censoring. Statistical Methods in Medical Research **27**(4), 1230–1239 (2018) https://doi.org/10.1177/0962280216658320

[12] Buyse, M.: Generalized pairwise comparisons of prioritized outcomes in the two-sample problem. Statistics in Medicine **29**(30), 3245–3257 (2010) https://doi.org/10.1002/sim.3923

[13] Rondeau, V., Mathoulin-Pelissier, S., Jacqmin-Gadda, H., Brouste, V., Soubeyran, P.: Joint frailty models for recurring events and death using maximum penalized likelihood estimation: application on cancer events. Biostatistics **8**(4), 708–721 (2007) https://doi.org/10.1093/biostatistics/kxl043

[14] Ghosh, D., Lin, D.Y.: Marginal regression models for recurrent and terminal events. Statistica Sinica **12**(3), 663–688 (2002)

[15] Claggett, B.L., McCaw, Z.R., Tian, L., McMurray, J.J.V., Jhund, P.S., Uno, H., Pfeffer, M.A., Solomon, S.D., Wei, L.-J.: Quantifying treatment effects in trials with multiple event-time outcomes. NEJM Evidence **1**(10), 2200047 (2022) https://doi.org/10.1056/EVIDoa2200047

[16] Wei, J., Mütze, T., Jahn-Eimermacher, A., Roger, J.: Properties of two while-alive estimands for recurrent events and their potential estimators. Statistics in Biopharmaceutical Research **15**(2), 257–267 (2023) https://doi.org/10.1080/19466315.2021.1994457

[17] Mao, L.: Nonparametric inference of general while-alive estimands for recurrent events. Biometrics **79**(3), 1749–1760 (2023) https://doi.org/10.1111/biom.13709

[18] Cook, R., Lawless, J.: Marginal analysis of recurrent events and a terminating event. Stat Med **16**(8), 911–924 (1997) https://doi.org/10.1002/(sici)1097-0258(19970430)

[19] Cheng, B., Cook, R.: Tests for multivariate recurrent events in the presence of a terminal event. Biostatistics **5**(1), 129–143 (2004) https://doi.org/10.1093/biostatistics/5.1.129

[20] Wang, M., Qin, J., Chinag, C.: Analyzing Recurrent Event Data With Informative Censoring. J Am Stat Assoc **96**(455), 1057–1065 (2001) https://doi.org/10.1198/016214501753209031

[21] Liu, L., Wolfe, R., Huang, X.: Shared Frailty Models for Recurrent Events and a Terminal Event. Biometrics **60**(3), 747–756 (2004) https://doi.org/10.1111/j.0006-341X.2004.00225.x

[22] Huang, C., Wang, M.: Joint Modeling and Estimation for Recurrent Event Processes and Failure Time Data. J Am Stat Assoc **99**(468), 1153–1165 (2004) https://doi.org/10.1198/016214504000001033

[23] Ye, Y., Kalbfleisch, J., Schaubel, D.: Semiparametric Analysis of Correlated Recurrent and Terminal Events. Biometrics **63**(1), 78–87 (2007) https://doi.org/

0.1111/j.1541-0420.2006.00677.x

[24] Zeng, D., Lin, D.: Semiparametric Transformation Models with Random Effects for Joint Analysis of Recurrent and Terminal Events. Biometrics **65**(3), 746–752 (2009) https://doi.org/0.1111/j.1541-0420.2008.01126.x.

[25] Schaubel, D., Zeng, D., Cai, J.: A semiparametric additive rates model for recurrent event data. Lifetime Data Anal **12**(4), 389–406 (2006) https://doi.org/10.1007/s10985-006-9017-x

[26] Zeng, D., Cai, J.: A semiparametric additive rate model for recurrent events with an informative terminal event. Biometrika **97**(3), 699–712 (2010) https://doi.org/0.1093/biomet/asq039

[27] Osmani, F., Hajizadeh, E., Rasekhi, A.: Bayesian Multivariate Joint Modeling for Multiple Events Time Data and Survival. Preprint with the Lancet (2018) https://doi.org/10.2139/ssrn.3255840

[28] Li, Z., Chinchilli, V., Wang, M.: A Bayesian joint model of recurrent events and a terminal event. Biom J **61**, 187–202 (2019) https://doi.org/10.1002/bimj.201700326

[29] Paulon, G., De Iorio, M., Guglielmi, A., Ieva, F.: Joint modeling of recurrent events and survival: a Bayesian non-parametric approach. Biostatistics **21**, 1–14 (2020) https://doi.org/10.1093/biostatistics/kxy026

[30] van den Boom, W., De Iorio, M., Tallarita, M.: Bayesian inference on the number of recurrent events: A joint model of recurrence and survival. Stat Methods Med Res **31**, 139–152 (2022) https://doi.org/10.1177/09622802211048059

[31] Furberg, J., Andersen, P., Korn, S., Overgaard, M., Ravn, H.: Bivariate pseudo-observations for recurrent event analysis with terminal events. Lifetime Data Analysis **29**, 256–287 (2023) https://doi.org/10.1007/s10985-021-09533-5

[32] Furberg, J.: recurrentpseudo: An R package for analysing recurrent events in the presence of terminal events using pseudo-observations (2022). https://cran.r-project.org/web/packages/recurrentpseudo/vignettes/recurrentpseudo.html.accessed2025

[33] Mao, L.: On restricted mean time in favor of treatment. Biometrics **79**(1), 61–72 (2023) https://doi.org/10.1111/biom.13570

[34] Mao, L.: rmt: Restricted Mean Time in Favor of Treatment (2021). https://cran.r-project.org/web/packages/rmt/index.html.accessed2025

[35] Entrop, J., Jakobsen, L., Crowther, M., Clements, M., Eloranta, S., Dietrich, C.:

Parametric estimation of the mean number of events in the presence of competing risks. Biometrical Journal **67**(1), 1–11 (2025) https://doi.org/10.1002/bimj.70038

[36] Entrop, J.: JointFPM: A Parametric Model for Estimating the Mean Number of Events (2025). https://cran.r-project.org/web/packages/JointFPM/index.html. accessed2026
