# Supplementary material for: Methods for analyzing recurrent and terminal events considering composite and competing endpoints in clinical trials: a practical overview
Source: BMC Med Res Methodol. 2026 Sep 12;26:206. doi: 10.1186/s12874-026-03008-7 (PMC13571326; doi:10.1186/s12874-026-03008-7)
Supplement: Supplementary file 2 [file 12874_2026_3008_MOESM2_ESM.pdf]

# Appendix 2: Methods for Analyzing Recurrent and Terminal Events considering composite and competing endpoints in Clinical Trials : A Practical Overview

R code and (further) results

Alidan Duoerkongjiang, Antonia Zapf, Paulus Kirchhof, and Ann-Kathrin Ozga

## Contents

**Load packages** **3**

**R code for application** **4**

- Composite endpoint not considering clinical relevance . . . 4
  - Data set structure for the AG, LWYY, and parametric models . . . 4
  - AG model . . . 4
  - LWYY model . . . 4
  - Parametric time-to-event model with all events of all endpoints . . . 5
  - Data set structure for the PWP and stratified parametric model . . . 6
  - PWP model . . . 6
  - Stratified parametric time-to-event model . . . 6
  - Data set structure for WLW model . . . 7
  - WLW model . . . 7
  - Data structure for negative binomial model . . . 8
  - Negative binomial . . . 8
- Composite endpoint considering clinical relevance via weights . . . 10
  - Data set structure for weighted LWYY model . . . 10
  - Weighted LWYY model . . . 10
  - Data set structure for the Weighted all-cause hazard ratio . . . 10
  - Weighted parametric all-cause hazard ratio . . . 11
  - Stratififed parametric Weighted all-cause hazard ratio . . . 11
  - Non-parametric Weighted all-cause hazard ratio . . . 11
  - Data set structure for Wei-Lachin approach . . . 12
  - Wei-Lachin approach . . . 12
  - Stratified Wei Lachin aproach . . . 12
  - Data set structure for the approach by Bakal et al. . . . 13
  - Bakal . . . 13
- Composite endpoint considering clinical relevance via ordered endpoints . . . 14
  - Data set structure for the Win ratio/Win odds and Generalized pairwise comparison . . . 14
  - Win ratio/Win odds . . . 14
  - Generalized pairwise comparison . . . 16
- Composite endpoint considering correlated competing events or no composite . . . 17
  - Data set structure for Joint Frailty and Ghosh and Lin models . . . 17
  - Semi parametric Joint Frailty model . . . 17
  - Parametric Joint Frailty . . . 19
  - Ghosh and Lin approach . . . 21

Data set structure for the a complex multistate model . . . 23
Complex multistate model - semi-parametric hazard ratios . . . 23
Data structure for AUC and While-Alive approach . . . 27
AUC . . . 27
While alive approach . . . 28
Further data description . . . 30
Data structure for Nelson-Aalen and Aalen-Johansen estimate (Version 1) . . . 30
Nelson Aalen . . . 30
Aalen Johansen . . . 32
Data structure for Nelson-Aalen and Aalen-Johansen estimate (Version 2) . . . 32
Nelson Aalen . . . 33
Aalen Johansen . . . 35
Figures to compare effect estimates . . . 38

**Software environment** **43**

## Load packages

```
# packages needed
library(tidyverse)    # data management
library(survival)     # basic survival analysis (for models: AG, PWP, WLW, LWYY, WHR, WL)
library(WINS)         # win ratio / win odds
library(frailtypack)  # joint frailty model
library(reReg)        # Ghosh and Lin
library(multcomp)     # for Wei-Lachin appraoch
library(flexsurv)     # parametric models
library(mvna)         # Nelson Aalen
library(etm)          # Aalen Johansen
library(Wcompo)       # weighted LWYY
library(BuyseTest)    # Generalized pairwise comparison
library(MCC)          # AUC
library(WA)           # While alive estimand
library(MASS)         # negative binomial model
library(emmeans)      # get effect estimate and CI
library(forestploter) # forestplot
library(ggpubr)       # arrange plots
library(cowplot)      # arrange plots
```

# R code for application

## Composite endpoint not considering clinical relevance

### Data set structure for the AG, LWYY, and parametric models

| subjectnr | study_arm | start_time | stop_time | statusCom |
|---|---|---|---|---|
| 1 | Control | 0 | 2505 | 1 |
| 1 | Control | 2505 | 3021 | 0 |
| 2 | Treatment | 0 | 3133 | 0 |
| 3 | Treatment | 0 | 1420 | 1 |
| 4 | Control | 0 | 3132 | 0 |
| 5 | Control | 0 | 1364 | 1 |

### AG model

```r
# model fit
fitAG <- coxph(Surv(time = start_time,  time2 = stop_time, event = statusCom) ~
                 study_arm, data = data)
# full model output
summary(fitAG)
```

```
## Call:
## coxph(formula = Surv(time = start_time, time2 = stop_time, event = statusCom) ~
##     study_arm, data = data)
##
##   n= 3562, number of events= 934
##
##                        coef exp(coef) se(coef)     z Pr(>|z|)
## study_armTreatment -0.27575   0.75900  0.06613 -4.17 3.05e-05 ***
## ---
## Signif. codes:  0 '***' 0.001 '**' 0.01 '*' 0.05 '.' 0.1 ' ' 1
##
##                    exp(coef) exp(-coef) lower .95 upper .95
## study_armTreatment     0.759      1.318    0.6667     0.864
##
## Concordance= 0.535  (se = 0.009 )
## Likelihood ratio test= 17.56  on 1 df,   p=3e-05
## Wald test            = 17.38  on 1 df,   p=3e-05
## Score (logrank) test = 17.5  on 1 df,   p=3e-05
```

```r
# save effect estimate and CI for plot
adj_eff <- contrast(emmeans(fitAG,~study_arm), method = "trt.vs.ctrl")
eff_AG <- confint(adj_eff ) %>% as_tibble() %>%
  dplyr::select(contrast, estimate, asymp.LCL, asymp.UCL)
```

### LWYY model

```r
# model fit
fitLWYY <- coxph(Surv(time =  start_time,  time2 =  stop_time, event = statusCom) ~
                   study_arm + cluster(subjectnr), data = data)
# full model output
summary(fitLWYY)
```

```
## Call:
## coxph(formula = Surv(time = start_time, time2 = stop_time, event = statusCom) ~
##     study_arm, data = data, cluster = subjectnr)
##
##   n= 3562, number of events= 934
##
##                         coef exp(coef) se(coef) robust se      z Pr(>|z|)
## study_armTreatment -0.27575   0.75900  0.06613   0.10110 -2.728  0.00638 **
## ---
## Signif. codes:  0 '***' 0.001 '**' 0.01 '*' 0.05 '.' 0.1 ' ' 1
##
##                    exp(coef) exp(-coef) lower .95 upper .95
## study_armTreatment     0.759      1.318    0.6226    0.9253
##
## Concordance= 0.535  (se = 0.013 )
## Likelihood ratio test= 17.56  on 1 df,   p=3e-05
## Wald test            = 7.44  on 1 df,   p=0.006
## Score (logrank) test = 17.5  on 1 df,   p=3e-05,   Robust = 7.25  p=0.007
##
##   (Note: the likelihood ratio and score tests assume independence of
##      observations within a cluster, the Wald and robust score tests do not).
```

```r
# save effect estimate and CI for plot
adj_eff <- contrast(emmeans(fitLWYY,~study_arm), method = "trt.vs.ctrl")
eff_LWYY <- confint(adj_eff ) %>% as_tibble() %>%
  dplyr::select(contrast, estimate, asymp.LCL, asymp.UCL)
```

**Parametric time-to-event model with all events of all endpoints**

```r
# model fit
para_AG <-flexsurvreg(Surv(start_time,stop_time,statusCom)~study_arm,
                      dist = "weibull", data = data)
# model output
para_AG
```

```
## Call:
## flexsurvreg(formula = Surv(start_time, stop_time, statusCom) ~
##     study_arm, data = data, dist = "weibull")
##
## Estimates:
##                    data mean  est       L95%      U95%      se        exp(est)
## shape                    NA   9.23e-01  8.68e-01  9.81e-01  2.87e-02        NA
## scale                    NA   5.14e+03  4.61e+03  5.73e+03  2.85e+02        NA
## study_armTreatment  4.85e-01   3.02e-01  1.60e-01  4.44e-01  7.23e-02  1.35e+00
##                    L95%      U95%
## shape                    NA        NA
## scale                    NA        NA
## study_armTreatment  1.17e+00  1.56e+00
##
## N = 3562,  Events: 934,  Censored: 2628
## Total time at risk: 5078139
## Log-likelihood = -8954.901, df = 3
## AIC = 17915.8
```

```r
# save effect plus CI for plot
p_AG <- exp(para_AG$res[3,1])
p_AG_CIL <- exp(para_AG$res[3,2])
p_AG_CIU <- exp(para_AG$res[3,3])
```

**Data set structure for the PWP and stratified parametric model**

| subjectnr | study_arm | start_time | stop_time | statusCom | enum |
|---|---|---|---|---|---|
| 1 | Control | 0 | 2505 | 1 | 1 |
| 1 | Control | 2505 | 3021 | 0 | 2 |
| 2 | Treatment | 0 | 3133 | 0 | 1 |
| 3 | Treatment | 0 | 1420 | 1 | 1 |
| 4 | Control | 0 | 3132 | 0 | 1 |
| 5 | Control | 0 | 1364 | 1 | 1 |

**PWP model**

```r
# model fit
fitPWP <- coxph(Surv(time = start_time,  time2 = stop_time, event = statusCom) ~
                  study_arm + strata(enum), data=data)

# save effect estimate and CI for plot
summary(fitPWP)
```

```
## Call:
## coxph(formula = Surv(time = start_time, time2 = stop_time, event = statusCom) ~
##     study_arm + strata(enum), data = data)
##
##   n= 3562, number of events= 934
##
##                       coef exp(coef) se(coef)      z Pr(>|z|)
## study_armTreatment -0.16211   0.85035  0.06769 -2.395   0.0166 *
## ---
## Signif. codes:  0 '***' 0.001 '**' 0.01 '*' 0.05 '.' 0.1 ' ' 1
##
##                    exp(coef) exp(-coef) lower .95 upper .95
## study_armTreatment    0.8504      1.176    0.7447     0.971
##
## Concordance= 0.53  (se = 0.011 )
## Likelihood ratio test= 5.77  on 1 df,   p=0.02
## Wald test            = 5.74  on 1 df,   p=0.02
## Score (logrank) test = 5.75  on 1 df,   p=0.02
```

```r
# save effect estimate and CI for plot
adj_eff <- contrast(emmeans(fitPWP,~study_arm), method = "trt.vs.ctrl")
eff_PWP <- confint(adj_eff ) %>% as_tibble() %>%
  dplyr::select(contrast, estimate, asymp.LCL, asymp.UCL)
```

**Stratified parametric time-to-event model**

```r
# model fit
para_PWP <-flexsurvreg(Surv(start_time,stop_time,statusCom)~study_arm + enum,
```

```r
                  anc = list(shape = ~ enum) , dist = "weibull", data = data)
# model output
para_PWP
```

```
## Call:
## flexsurvreg(formula = Surv(start_time, stop_time, statusCom) ~
##     study_arm + enum, anc = list(shape = ~enum), data = data,
##     dist = "weibull")
##
## Estimates:
##                       data mean  est        L95%       U95%       se
## shape                       NA    9.23e-01   8.55e-01   9.96e-01   3.61e-02
## scale                       NA    5.67e+04   3.85e+04   8.34e+04   1.12e+04
## study_armTreatment   4.85e-01    2.35e-01   5.79e-02   4.11e-01   9.02e-02
## enum                 1.39e+00   -1.75e+00  -2.00e+00  -1.50e+00   1.29e-01
## shape(enum)          1.39e+00   -1.43e-01  -1.71e-01  -1.14e-01   1.44e-02
##                       exp(est)   L95%       U95%
## shape                       NA          NA         NA
## scale                       NA          NA         NA
## study_armTreatment   1.26e+00    1.06e+00   1.51e+00
## enum                 1.74e-01    1.35e-01   2.23e-01
## shape(enum)          8.67e-01    8.43e-01   8.92e-01
##
## N = 3562,  Events: 934,  Censored: 2628
## Total time at risk: 5078139
## Log-likelihood = -8567.017, df = 5
## AIC = 17144.03
```

```r
# save effect plus CI for plot
p_PWP <- exp(para_PWP$res[3,1])
p_PWP_CIL <- exp(para_PWP$res[3,2])
p_PWP_CIU <- exp(para_PWP$res[3,3])
```

### Data set structure for WLW model

| subjectnr | study_arm | stop_time | statusCom | enum |
|---:|---|---:|---:|---:|
| 1 | Control | 2505 | 1 | 1 |
| 1 | Control | 3021 | 0 | 2 |
| 1 | Control | 3021 | 0 | 3 |
| 1 | Control | 3021 | 0 | 4 |
| 1 | Control | 3021 | 0 | 5 |
| 1 | Control | 3021 | 0 | 6 |

### WLW model

```r
# model fit
fitWLW <- coxph(Surv(time = stop_time, event = statusCom) ~
                  study_arm + strata(enum), data=dataWLW)

# save effect estimate and CI for plot
summary(fitWLW)
```

```
## Call:
```

```
## coxph(formula = Surv(time = stop_time, event = statusCom) ~ study_arm +
##     strata(enum), data = dataWLW)
##
##   n= 41835, number of events= 934
##
##                       coef exp(coef) se(coef)      z Pr(>|z|)
## study_armTreatment -0.29076   0.74769  0.06614 -4.396  1.1e-05 ***
## ---
## Signif. codes:  0 '***' 0.001 '**' 0.01 '*' 0.05 '.' 0.1 ' ' 1
##
##                    exp(coef) exp(-coef) lower .95 upper .95
## study_armTreatment    0.7477      1.337    0.6568    0.8512
##
## Concordance= 0.537  (se = 0.009 )
## Likelihood ratio test= 19.53  on 1 df,   p=1e-05
## Wald test            = 19.33  on 1 df,   p=1e-05
## Score (logrank) test = 19.47  on 1 df,   p=1e-05
```

```
# save effect estimate and CI for plot
adj_eff <- contrast(emmeans(fitWLW,~study_arm), method = "trt.vs.ctrl")
eff_WLW <- confint(adj_eff ) %>% as_tibble() %>%
  dplyr::select(contrast, estimate, asymp.LCL, asymp.UCL)
```

### Data structure for negative binomial model

| subjectnr | n | offset | study_arm | offsetlg |
|---|---|---|---|---|
| 1 | 1 | 2505 | Control | 7.826044 |
| 3 | 1 | 1420 | Treatment | 7.258412 |
| 5 | 2 | 2507 | Control | 7.826842 |
| 6 | 1 | 2050 | Control | 7.625595 |
| 7 | 1 | 783 | Treatment | 6.663133 |
| 8 | 1 | 1953 | Treatment | 7.577122 |

### Negative binomial

```
# model fit
fitNb <- glm.nb(n~study_arm+offset(offsetlg), data=data_count_all)
# model output
summary(fitNb)
```

```
##
## Call:
## glm.nb(formula = n ~ study_arm + offset(offsetlg), data = data_count_all,
##     init.theta = 0.1280973446, link = log)
##
## Coefficients:
##                    Estimate Std. Error z value Pr(>|z|)
## (Intercept)        -7.75261    0.08345 -92.903  < 2e-16 ***
## study_armTreatment -0.34282    0.12023  -2.851  0.00435 **
## ---
## Signif. codes:  0 '***' 0.001 '**' 0.01 '*' 0.05 '.' 0.1 ' ' 1
##
## (Dispersion parameter for Negative Binomial(0.1281) family taken to be 1)
```

```
##
##     Null deviance: 1557.9  on 2788  degrees of freedom
## Residual deviance: 1551.9  on 2787  degrees of freedom
## AIC: 4698
##
## Number of Fisher Scoring iterations: 1
##
##
##               Theta:  0.12810
##           Std. Err.:  0.00864
##
##  2 x log-likelihood:  -4691.96200
```

```r
# effects plus CI
adj_eff <- contrast(emmeans(fitNb,~study_arm,  offset = 0, type="response"),
                    method = "trt.vs.ctrl")
# save effect estimate and CI for plot
eff_NB <- confint(adj_eff ) %>% as_tibble() %>%
  dplyr::select(contrast, ratio, asymp.LCL, asymp.UCL)
# effect output
eff_NB
```

```
## # A tibble: 1 x 4
##   contrast            ratio asymp.LCL asymp.UCL
##   <fct>               <dbl>     <dbl>     <dbl>
## 1 Treatment / Control 0.710     0.561     0.898
```

### Composite endpoint considering clinical relevance via weights

#### Data set structure for weighted LWYY model

| subjectnr | study_armOld2 | stop_time | statusAll |
|---:|---:|---:|---:|
| 1 | 0 | 2505 | 3 |
| 1 | 0 | 3021 | 0 |
| 2 | 1 | 3133 | 0 |
| 3 | 1 | 1420 | 1 |
| 4 | 0 | 3132 | 0 |
| 5 | 0 | 1364 | 2 |

#### Weighted LWYY model

```
# model fit
fit_LWYYweight <- CompoML(id = data$subjectnr, time = data$stop_time,
                          status = data$statusAll, Z = data$study_armOld2,
                          w = c(1, 0.8,0.5,0.5))
# model output
fit_LWYYweight
```

```
##
## Call:
## CompoML(id = data$subjectnr, time = data$stop_time, status = data$statusAll,
##     Z = data$study_armOld2, w = c(1, 0.8, 0.5, 0.5))
##
## Proportional means regression models for composite endpoint
##  (Mao and Lin, 2016, Biostatistics):
##
##        Event 1 (Death) Event 2 Event 3 Event 4
## Weight               1     0.8     0.5     0.5
##
##
## Newton-Raphson algorithm converges in 2 iterations.
##
## Estimates for Regression parameters:
##
##       Estimate        se z.value  p.value
## [1,] -0.274956  0.095087 -2.8916 0.003832 **
## ---
## Signif. codes:  0 '***' 0.001 '**' 0.01 '*' 0.05 '.' 0.1 ' ' 1
##
##
## Point and interval estimates for the mean ratios:
##
##      Mean Ratio 95% lower CL 95% higher CL
## [1,]  0.7596056    0.6304495      0.915221
```

```
# save estimate plus CI for plot
eff_LWYYweight <- 0.7596056
eff_LWYYweightlCU <- 0.6304495
eff_LWYYweightuCU <-  0.915221
```

#### Data set structure for the Weighted all-cause hazard ratio

| subjectnr | study_arm | start_time | stop_time | statusRec | statusCVdeath | enum |
|---|---|---|---|---|---|---|
| 1 | Control | 0 | 2505 | 2 | 0 | 1 |
| 1 | Control | 2505 | 3021 | 0 | 0 | 2 |
| 2 | Treatment | 0 | 3133 | 0 | 0 | 1 |
| 3 | Treatment | 0 | 1420 | 0 | 1 | 1 |
| 4 | Control | 0 | 3132 | 0 | 0 | 1 |
| 5 | Control | 0 | 1364 | 3 | 0 | 1 |

### Weighted parametric all-cause hazard ratio

```
# use own function
out <- WHR_function_para(data,id="subjectnr" ,start="start_time", stop="stop_time",
                       statusR="statusRec",statusD = "statusCVdeath",
                       group="study_arm",eventNr = 4,
                       weights = c(0.5,0.5,0.8,1), bootNr=1000)

out
```

```
## weighted all-cause HR              CI lower              CI upper
##             0.8054679             0.6100691             1.0811864
```

```
# save estimate and CI for plot
WHR_p <- out[1]
ciHRW_p <- out[2:3]
```

### Stratififed parametric Weighted all-cause hazard ratio

```
# use own function
out <- WHR_function_para(data,id="subjectnr" ,start="start_time", stop="stop_time",
                       statusR="statusRec",statusD = "statusCVdeath",
                       group="study_arm",eventNr = 4, enum="enum",
                       weights = c(0.5,0.5,0.8,1), bootNr=1000)

out
```

```
## weighted all-cause HR              CI lower              CI upper
##            0.66425627            0.02329356           11.92294150
```

```
#save estimate and CI for plot
WHR_p2 <- out[1]
ciHRW_p2 <- out[2:3]
```

### Non-parametric Weighted all-cause hazard ratio

```
# use own function
out <- WHR_function_np(data,id="subjectnr" ,start="start_time", stop="stop_time",
                       statusR="statusRec",statusD = "statusCVdeath",
                       group="study_arm",eventNr = 4, enum="enum",
                       weights = c(0.5,0.5,0.8,1), bootNr=1000)

out
```

```
## weighted all-cause HR              CI lower              CI upper
##             0.7101451             0.4204919             1.0305256
```

```r
# save estimate and CI for plot
WHR <- out[1]
ciHRW <- out[2:3]
```

### Data set structure for Wei-Lachin approach

| subjectnr | group | start_time | stop | statusRec | statusCVdeath | enum |
|---|---|---|---|---|---|---|
| 1 | Control | 0 | 2505 | 2 | 0 | 1 |
| 1 | Control | 2505 | 3021 | 0 | 0 | 2 |
| 2 | Treatment | 0 | 3133 | 0 | 0 | 1 |
| 3 | Treatment | 0 | 1420 | 0 | 1 | 1 |
| 4 | Control | 0 | 3132 | 0 | 0 | 1 |
| 5 | Control | 0 | 1364 | 3 | 0 | 1 |

### Wei-Lachin approach

```r
# use own function
out <- WL_function_sp(data,  start="start_time", stop="stop_time", statusR="statusRec",
               statusD = "statusCVdeath",group="study_arm",eventNr = 4,
               weights = c(1-((1+0.5+0.8)/(0.5+0.5+0.8+1)),
                        1-((1+0.5+0.8)/(0.5+0.5+0.8+1)),
                        1-((1+0.5+0.5)/(0.5+0.5+0.8+1)),
                        1/(0.5+0.5+0.8+1)))

out
```

```
## weighted HR    CI lower    CI upper
##   0.7552759   0.5840844   0.9264674
```

```r
# save estimate and CI for plot
hrWL2 <- out[1]
hrWL_CIL2 <- out[2]
hrWL_CIU2 <- out[3]
```

### Stratified Wei Lachin aproach

```r
# use own function
out <- WL_function_sp(data,  start="start_time", stop="stop_time", statusR="statusRec",
               statusD = "statusCVdeath",group="study_arm",eventNr = 4, enum="enum",
               weights = c(1-((1+0.5+0.8)/(0.5+0.5+0.8+1)),
                        1-((1+0.5+0.8)/(0.5+0.5+0.8+1)),
                        1-((1+0.5+0.5)/(0.5+0.5+0.8+1)),
                        1/(0.5+0.5+0.8+1)))

out
```

```
## weighted HR    CI lower    CI upper
##   0.8151937   0.6437992   0.9865882
```

```r
# save estimate and CI for plot
hrWL <- out[1]
hrWL_CIL <- out[2]
hrWL_CIU <- out[3]
```

**Data set structure for the approach by Bakal et al.**

| subjectnr | study_arm | stop_time | statusRec | statusCVdeath | enum |
|---:|---|---:|---:|---:|---:|
| 1 | Control | 2505 | 2 | 0 | 1 |
| 1 | Control | 3021 | 0 | 0 | 2 |
| 2 | Treatment | 3133 | 0 | 0 | 1 |
| 3 | Treatment | 1420 | 0 | 1 | 1 |
| 4 | Control | 3132 | 0 | 0 | 1 |
| 5 | Control | 1364 | 3 | 0 | 1 |

**Bakal**

```
# use own function
Bakal_function(data,id="subjectnr", stop="stop_time", statusR="statusRec",
               statusD="statusCVdeath", group="study_arm",
               weights = c(0.5,0.5,0.8,1), eventNr = 4)
```

```
## [1] "p-value: 0.00214966290885847"
```

### Composite endpoint considering clinical relevance via ordered endpoints

#### Data set structure for the Win ratio/Win odds and Generalized pairwise comparison

| id | arm | Delta_1 | Y_1 | Y_2 | Delta_3 | Y_3 | Y_4 | Delta_5 | Y_5 | Y_6 | Delta_7 | Y_7 |
|---|---|---|---|---|---|---|---|---|---|---|---|---|
| 1 | Control | 0 | 3021 | 0 | 0 | 3021 | 1 | 1 | 2505 | 0 | 0 | 3021 |
| 2 | Treatment | 0 | 3133 | 0 | 0 | 3133 | 0 | 0 | 3133 | 0 | 0 | 3133 |
| 3 | Treatment | 1 | 1420 | 0 | 0 | 1420 | 0 | 0 | 1420 | 0 | 0 | 1420 |
| 4 | Control | 0 | 3132 | 0 | 0 | 3132 | 0 | 0 | 3132 | 0 | 0 | 3132 |
| 5 | Control | 0 | 3129 | 1 | 1 | 1364 | 1 | 1 | 2507 | 0 | 0 | 3129 |
| 6 | Control | 0 | 3125 | 0 | 0 | 3125 | 1 | 1 | 2050 | 0 | 0 | 3125 |

#### Win ratio/Win odds

```
# model fit
res_tte <- win.stat(data = dat_for_func2,
                    ep_type = c("tte","continuous","tte","continuous","tte",
                                "continuous","tte"),
                    arm.name = c("Treatment","Control"),

                    priority = c(1,2,3,4,5,6,7), alpha = 0.05, digit = 5,
                    np_direction = c("larger","smaller","larger","smaller",
                                     "larger","smaller","larger"),

                    stratum.weight = "unstratified", pvalue = "two-sided",
                    summary.print = FALSE)
```

```
## This analysis is unstratified.
```

```
# model output
res_tte
```

```
## $Win_prop
##   stratum     P_trt     P_con
## 1       1 0.2018389 0.1538694
##
## $Win_statistic
## $Win_statistic$Win_Ratio
##       WR     WR_L     WR_U
## 1.311755 1.111081 1.548673
##
## $Win_statistic$Net_Benefit
##         NB       NB_L       NB_U
## 0.04796954 0.01843992 0.07749915
##
## $Win_statistic$Win_Odds
##       WO     WO_L     WO_U
## 1.100773 1.037645 1.167742
##
##
## $z_statistic
##       zstat_WR zstat_NB zstat_WO
## value 3.203388 3.183874 3.186319
##
```

```
## $p_value
##          pvalue_WR   pvalue_NB   pvalue_WO
## value 0.001358208 0.001453183 0.001440955
##
## $summary_ep
## $summary_ep$Trt_Endpoint1
##   Stratum Count Proportion
## 1       1 99344 0.05108632
##
## $summary_ep$Con_Endpoint1
##   Stratum Count Proportion
## 1       1 72722 0.03739632
##
## $summary_ep$Trt_Endpoint2
##   Stratum Count Proportion
## 1       1 69324 0.03564894
##
## $summary_ep$Con_Endpoint2
##   Stratum Count Proportion
## 1       1 47637 0.02449669
##
## $summary_ep$Trt_Endpoint3
##   Stratum Count   Proportion
## 1       1   777 0.0003995619
##
## $summary_ep$Con_Endpoint3
##   Stratum Count   Proportion
## 1       1   899 0.0004622987
##
## $summary_ep$Trt_Endpoint4
##   Stratum  Count Proportion
## 1       1 171989 0.08844305
##
## $summary_ep$Con_Endpoint4
##   Stratum  Count Proportion
## 1       1 131518 0.06763137
##
## $summary_ep$Trt_Endpoint5
##   Stratum Count  Proportion
## 1       1  3677 0.001890848
##
## $summary_ep$Con_Endpoint5
##   Stratum Count  Proportion
## 1       1  3846 0.001977754
##
## $summary_ep$Trt_Endpoint6
##   Stratum Count Proportion
## 1       1 46821 0.02407707
##
## $summary_ep$Con_Endpoint6
##   Stratum Count Proportion
## 1       1 42033 0.02161491
##
## $summary_ep$Trt_Endpoint7
```

```
##   Stratum Count   Proportion
## 1       1   570 0.0002931149
##
## $summary_ep$Con_Endpoint7
##   Stratum Count   Proportion
## 1       1   564 0.0002900295
```

```r
# save inverse effects plus CI for plot
WR <- 1/res_tte$Win_statistic$Win_Ratio["WR"]
WR_CIL <- 1/res_tte$Win_statistic$Win_Ratio["WR_L"]
WR_CIU <- 1/res_tte$Win_statistic$Win_Ratio["WR_U"]

WO <- 1/res_tte$Win_statistic$Win_Odds["WO"]
WO_CIL <- 1/res_tte$Win_statistic$Win_Odds["WO_L"]
WO_CIU <- 1/res_tte$Win_statistic$Win_Odds["WO_U"]
```

#### Generalized pairwise comparison

```r
# model fit
BT <- BuyseTest(arm ~ TTE(Y_1, status=Delta_1)+ cont(Y_2) + TTE(Y_3, status=Delta_3)+
                  cont(Y_4)+ TTE(Y_5, status=Delta_5)+ cont(Y_6)+ TTE(Y_7, status=Delta_7),
                hierarchical = TRUE, data=dat_for_func2, trace = FALSE)

# full model output
summary(BT)
```

```
##        Generalized pairwise comparisons with 7 prioritized endpoints
##
##  - statistic       : net treatment benefit  (delta: endpoint specific, Delta: global)
##  - null hypothesis : Delta == 0
##  - confidence level: 0.95
##  - inference       : H-projection of order 1 after atanh transformation
##  - treatment groups: Treatment (treatment) vs. Control (control)
##  - censored pairs  : probabilistic score based on the survival curves
##  - neutral pairs   : re-analyzed using lower priority endpoints
##  - uninformative pairs: no contribution at the current endpoint, analyzed at later endpoints
##  - results
##  endpoint total(%) favorable(%) unfavorable(%) neutral(%) uninf(%)   delta
##       Y_1   100.00        11.95           7.21       0.00    80.83  0.0474
##       Y_2    80.83         2.18           2.91      75.74     0.00 -0.0073
##       Y_3    75.74         7.11           1.35       0.00    67.28  0.0576
##       Y_4    67.28         5.20           6.85      55.23     0.00 -0.0165
##       Y_5    55.23         7.15           8.02       0.00    40.06 -0.0087
##       Y_6    40.06         1.25           1.39      37.43     0.00 -0.0014
##       Y_7    37.43         1.76           2.14       0.00    33.53 -0.0038
##   Delta CI [2.5% ; 97.5%]  p.value
##  0.0474  [-0.0055;0.1001] 0.079062 .
##  0.0401  [-0.0142;0.0942] 0.147537
##  0.0977  [-0.0073;0.2006] 0.068173 .
##  0.0812   [-0.027;0.1875] 0.141015
##  0.0725  [-0.0497;0.1927] 0.244661
##  0.0712  [-0.0513;0.1916] 0.254763
##  0.0674   [-0.0635;0.196] 0.312808
```

## Composite endpoint considering correlated competing events or no composite

### Data set structure for Joint Frailty and Ghosh and Lin models

| subjectnr | study_arm | start_time | stop_time | statusCom | statusDnonCV | event_status | statusCVdeath |
|---|---|---|---|---|---|---|---|
| 1 | Control | 0 | 2505 | 1 | 0 | 1 | 0 |
| 1 | Control | 2505 | 3021 | 0 | 0 | 0 | 0 |
| 2 | Treatment | 0 | 3133 | 0 | 0 | 0 | 0 |
| 3 | Treatment | 0 | 1420 | 1 | 0 | 0 | 1 |
| 4 | Control | 0 | 3132 | 0 | 0 | 0 | 0 |
| 5 | Control | 0 | 1364 | 1 | 0 | 1 | 0 |

### Semi parametric Joint Frailty model

Model with composite in recurrent event part and competing event in terminal part

```
# model fit 1
fitJF <- frailtyPenal(Surv( start_time, stop_time, statusCom)~study_arm +
                        cluster(subjectnr) + terminal(statusDnonCV),
                      formula.terminalEvent=~study_arm,data=data,
                      n.knots=6,kappa=c(5000,5000),recurrentAG=TRUE)
```

```
##
## Be patient. The program is computing ...
## The program took 1214.39 seconds
```

```
fitJF
```

```
## Call:
## frailtyPenal(formula = Surv(start_time, stop_time, statusCom) ~
##     study_arm + cluster(subjectnr) + terminal(statusDnonCV),
##     formula.terminalEvent = ~study_arm, data = data, recurrentAG = TRUE,
##     n.knots = 6, kappa = c(5000, 5000))
##
##
##   Joint gamma frailty model for recurrent and a terminal event processes
##   using a Penalized Likelihood on the hazard function
##
## Recurrences:
## -------------
##                        coef exp(coef) SE coef (H) SE coef (HIH)        z
## study_armTreatment -0.297412  0.742738    0.078898      0.110339 -2.69544
##                           p
## study_armTreatment 0.0070295
##
## Terminal event:
## ----------------
##                         coef exp(coef) SE coef (H) SE coef (HIH)          z
## study_armTreatment 0.00116726   1.00117    0.177575      0.180711 0.00645926
##                         p
## study_armTreatment 0.99485
##
##  Frailty parameters:
##    theta (variance of Frailties, w): 1.0618 (SE (HIH): 0.0287563 ) p = < 1e-16
##    alpha (w^alpha for terminal event): 1.21257 (SE (HIH): 0.173335 ) p = 2.6427e-12
```

```
##
## Penalized marginal log-likelihood = -0.24
##    Convergence criteria:
##    parameters = 3.76e-07 likelihood = 0.000117 gradient = 9.36e-07
##
## Likelihood Cross-Validation (LCV) criterion in the semi parametrical case:
##    approximate LCV = 0.00641236
##
##    n observations= 3562  n subjects= 2789
##    n recurrent events= 934
##    n terminal events= 141
##    n censored events= 2628
##    number of iterations:  8
##    Number of nodes for the Gauss-Laguerre quadrature:  20
##
##    Exact number of knots used:  6
##    Value of the smoothing parameters:  5000 5000
```

```
# extract effect and CI for plot
effJF <- (fitJF$coef)
ciJFU <- ((fitJF$coef+1.96*sqrt(diag(fitJF$varH))[3:4]))
ciJFL <- ((fitJF$coef-1.96*sqrt(diag(fitJF$varH))[3:4]))
```

Model with non-fatal events in recurrent event part and cv death in terminal part

```
# model fit 2
fitJF2 <- frailtyPenal(Surv( start_time, stop_time, event_status)~study_arm +
                         cluster(subjectnr) + terminal(statusCVdeath),
                       formula.terminalEvent=~study_arm,data=data,
                       n.knots=6,kappa=c(5000,5000),recurrentAG=TRUE)
```

```
##
## Be patient. The program is computing ...
## The program took 1458.03 seconds
```

```
fitJF2
```

```
## Call:
## frailtyPenal(formula = Surv(start_time, stop_time, event_status) ~
##     study_arm + cluster(subjectnr) + terminal(statusCVdeath),
##     formula.terminalEvent = ~study_arm, data = data, recurrentAG = TRUE,
##     n.knots = 6, kappa = c(5000, 5000))
##
##
##   Joint gamma frailty model for recurrent and a terminal event processes
##   using a Penalized Likelihood on the hazard function
##
## Recurrences:
## -------------
##                         coef exp(coef) SE coef (H) SE coef (HIH)        z
## study_armTreatment -0.291912  0.746834   0.0841294      0.117073 -2.49343
##                           p
## study_armTreatment 0.012652
##
## Terminal event:
## ----------------
##                         coef exp(coef) SE coef (H) SE coef (HIH)        z
```

```
## study_armTreatment -0.360821  0.697104    0.187409      0.191329 -1.88586
##                            p
## study_armTreatment 0.059314
##
##  Frailty parameters:
##    theta (variance of Frailties, w): 1.03215 (SE (HIH): 0.0285069 ) p = < 1e-16
##    alpha (w^alpha for terminal event): 2.10456 (SE (HIH): 0.269425 ) p = 5.6621e-15
##
## Penalized marginal log-likelihood = -0.21
##    Convergence criteria:
##    parameters = 3.65e-06 likelihood = 0.000511 gradient = 2.21e-06
##
## Likelihood Cross-Validation (LCV) criterion in the semi parametrical case:
##    approximate LCV = 0.00615395
##
##    n observations= 3562  n subjects= 2789
##    n recurrent events= 773
##    n terminal events= 161
##    n censored events= 2789
##    number of iterations:  9
##    Number of nodes for the Gauss-Laguerre quadrature:  20
##
##    Exact number of knots used:  6
##    Value of the smoothing parameters:  5000 5000
```

```r
# extract effect and CI for plot
effJF2 <- (fitJF2$coef)
ciJFU2 <- ((fitJF2$coef+1.96*sqrt(diag(fitJF2$varH))[3:4]))
ciJFL2 <- ((fitJF2$coef-1.96*sqrt(diag(fitJF2$varH))[3:4]))
```

### Parametric Joint Frailty

Model with composite in recurrent event part and competing event in terminal part

```r
# model fit 1
fitJFp <- frailtyPenal(Surv( start_time, stop_time, statusCom)~
                         study_arm + cluster(subjectnr)+
                         terminal(statusDnonCV),
                       formula.terminalEvent=~study_arm,data=data,hazard = "Weibull")
```

```
##
## Be patient. The program is computing ...
## The program took 20.9 seconds
```

```r
# model output
fitJFp
```

```
## Call:
## frailtyPenal(formula = Surv(start_time, stop_time, statusCom) ~
##     study_arm + cluster(subjectnr) + terminal(statusDnonCV),
##     formula.terminalEvent = ~study_arm, data = data, hazard = "Weibull")
##
##
##   Joint gamma frailty model for recurrent and a terminal event processes
##   using a Parametrical approach for the hazard function
##
```

```
## Recurrences:
## -------------
##                        coef exp(coef) SE coef (H)        z          p
## study_armTreatment -0.298122  0.742211   0.0790045 -3.77348 0.00016099
##
## Terminal event:
## ----------------
##                        coef exp(coef) SE coef (H)        z       p
## study_armTreatment 0.0758306   1.07878    0.168526 0.449964 0.65274
##
##  Frailty parameters:
##    theta (variance of Frailties, w): 1.13726 (SE (HIH): 0.0489395 ) p = < 1e-16
##    alpha (w^alpha for terminal event): 0.0526725 (SE (HIH): 0.125761 ) p = 0.67534
##
##    marginal log-likelihood = -10325.31
##    Convergence criteria:
##    parameters = 0.000148 likelihood = 5.72e-07 gradient = 4.14e-12
##
##    AIC = Aikaike information Criterion     = 2.90099
##
## The expression of the Aikaike Criterion is:
##         'AIC = (1/n)[np - l(.)]'
##
##       Scale for the weibull hazard function is : 4642.11 22847.6
##       Shape for the weibull hazard function is : 0.95 1.29
##
## The expression of the Weibull hazard function is:
##         'lambda(t) = (shape.(t^(shape-1)))/(scale^shape)'
## The expression of the Weibull survival function is:
##         'S(t) = exp[- (t/scale)^shape]'
##
##    n observations= 3562  n subjects= 2789
##    n recurrent events= 934
##    n terminal events= 141
##    n censored events= 2628
##    number of iterations:  22
##    Number of nodes for the Gauss-Laguerre quadrature:  32
```

```
# save effect plus CI for plot
effJFp <- (fitJFp$coef)
ciJFUp <- ((fitJFp$coef+1.96*sqrt(diag(fitJFp$varH))[3:4]))
ciJFLp <- ((fitJFp$coef-1.96*sqrt(diag(fitJFp$varH))[3:4]))
```

Model with non-fatal events in recurrent event part and cv death in terminal part

```
# model fit 2
fitJF2p <- frailtyPenal(Surv( start_time, stop_time, event_status)~
                          study_arm + cluster(subjectnr)+
                          terminal(statusCVdeath),
                        formula.terminalEvent=~study_arm,data=data,
                        hazard = "Weibull",  RandDist = "LogN")
```

```
##
## Be patient. The program is computing ...
```

```
## The program took 6.78 seconds
```

```r
# model output
fitJF2p
```

```
## Call:
## frailtyPenal(formula = Surv(start_time, stop_time, event_status) ~
##     study_arm + cluster(subjectnr) + terminal(statusCVdeath),
##     formula.terminalEvent = ~study_arm, data = data, hazard = "Weibull",
##     RandDist = "LogN")
##
##
##   Joint Log-Normal frailty model for recurrent and a terminal event processes
##   using a Parametrical approach for the hazard function
##
##    Convergence criteria:
##    parameters = 31.1 likelihood = 84.2 gradient = 1
##
##    n= 3562
##    n recurrent events= 773
##    n terminal events= 161
##    Number of nodes for the Gauss-Hermite quadrature:  32
```

### Ghosh and Lin approach

```r
# datamanagement
data <- data %>% mutate(status_num=as.numeric(event_status),
                        statusCVdeath_num=as.numeric(statusCVdeath))
```

```r
# model fit
fm2 <- with(data, Recur(time=start_time %2% stop_time, id=subjectnr, event=status_num,
                        terminal=statusCVdeath_num)~ study_arm)
fitGL2 <- reReg(fm2, data = data, model = "cox.GL", B = 200)

# full model output
summary(fitGL2)
```

```
## Call:
## reReg(formula = fm2, data = data, model = "cox.GL", B = 200)
##
## Fitted with the Cox model of Ghosh and Lin (2002):
## Recurrent event process:
##                     Estimate   StdErr z.value p.value
## study_armTreatment -0.26422  0.10732  -2.462 0.01382 *
##
## Terminal event:
##                     Estimate   StdErr z.value p.value
## study_armTreatment -0.32627  0.15991 -2.0403 0.04132 *
```

```r
# effect
effGL_rec <- summary(fitGL2)$coefficients.rec[1]
effGL_D <- summary(fitGL2)$coefficients.haz[1]

# CI
effGL_rec_CIL <- summary(fitGL2)$coefficients.rec[1]-1.96*summary(fitGL2)$coefficients.rec[2]
effGL_rec_CIU <- summary(fitGL2)$coefficients.rec[1]+1.96*summary(fitGL2)$coefficients.rec[2]
```

```
effGL_D_CIL <- summary(fitGL2)$coefficients.haz[1]-1.96*summary(fitGL2)$coefficients.haz[2]
effGL_D_CIU <- summary(fitGL2)$coefficients.haz[1]+1.96*summary(fitGL2)$coefficients.haz[2]
```

### Data set structure for the a complex multistate model

| ID | start | stop | status | Group1 | Group2 | Group3 | Group4 | Group5 | Group6 | Group7 | Group8 | Group9 | Group10 |
|---|---|---|---|---|---|---|---|---|---|---|---|---|---|
| 1 | 0 | 2505 | 1 | 0 | 0 | 0 | 0 | 0 | 0 | 0 | 0 | 0 | 0 |
| 1 | 0 | 2505 | 0 | 0 | 0 | 0 | 0 | 0 | 0 | 0 | 0 | 0 | 0 |
| 1 | 0 | 2505 | 0 | 0 | 0 | 0 | 0 | 0 | 0 | 0 | 0 | 0 | 0 |
| 1 | 0 | 2505 | 0 | 0 | 0 | 0 | 0 | 0 | 0 | 0 | 0 | 0 | 0 |
| 1 | 0 | 2505 | 0 | 0 | 0 | 0 | 0 | 0 | 0 | 0 | 0 | 0 | 0 |
| 1 | 2505 | 3021 | 0 | 0 | 0 | 0 | 0 | 0 | 0 | 0 | 0 | 0 | 0 |

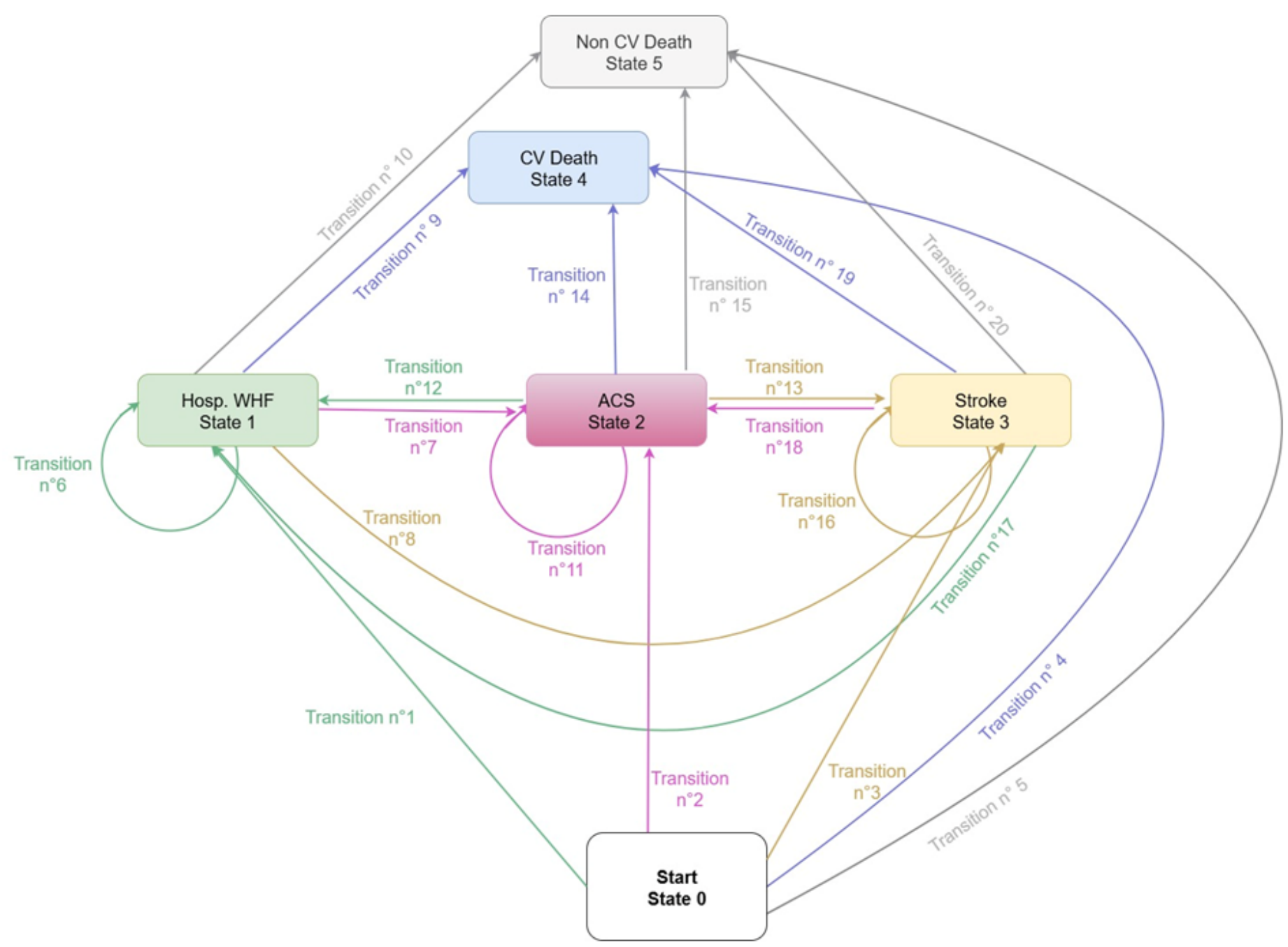


Figure 1: Multi-state

### Complex multistate model - semi-parametric hazard ratios

```
# model fit
fit1 <- coxph(Surv(start, stop, status) ~ Group1+Group2+Group3+Group4+Group5+
                Group6+ Group7+Group8+Group9+Group10+Group11+Group12+Group13+
                Group14+Group15+Group16+Group17+Group18+Group19+Group20+
                strata(trans) , data = datAllMulit)

# full model output
summary(fit1)
```

```
## Call:
## coxph(formula = Surv(start, stop, status) ~ Group1 + Group2 +
##     Group3 + Group4 + Group5 + Group6 + Group7 + Group8 + Group9 +
##     Group10 + Group11 + Group12 + Group13 + Group14 + Group15 +
##     Group16 + Group17 + Group18 + Group19 + Group20 + strata(trans),
##     data = datAllMulit)
```

```
##
##   n= 17810, number of events= 1075
##
##               coef  exp(coef)   se(coef)      z Pr(>|z|)
## Group1  -1.798e-01  8.354e-01  1.200e-01 -1.499   0.1339
## Group2  -2.054e-01  8.143e-01  1.926e-01 -1.067   0.2861
## Group3  -3.563e-01  7.003e-01  2.177e-01 -1.636   0.1017
## Group4  -3.978e-01  6.718e-01  2.160e-01 -1.842   0.0655 .
## Group5   1.209e-01  1.129e+00  2.113e-01  0.572   0.5672
## Group6  -1.276e-01  8.802e-01  1.432e-01 -0.891   0.3727
## Group7  -2.994e-01  7.412e-01  7.112e-01 -0.421   0.6737
## Group8  -7.182e-01  4.876e-01  6.791e-01 -1.058   0.2903
## Group9   3.267e-01  1.386e+00  2.998e-01  1.090   0.2758
## Group10  1.787e-01  1.196e+00  3.457e-01  0.517   0.6051
## Group11  1.794e-01  1.196e+00  4.390e-01  0.409   0.6828
## Group12 -8.925e-01  4.096e-01  4.808e-01 -1.856   0.0634 .
## Group13 -8.038e-01  4.476e-01  1.167e+00 -0.689   0.4909
## Group14 -1.822e-01  8.334e-01  5.604e-01 -0.325   0.7450
## Group15  2.912e-01  1.338e+00  7.644e-01  0.381   0.7032
## Group16  7.399e-01  2.096e+00  6.485e-01  1.141   0.2539
## Group17  1.804e-01  1.198e+00  5.588e-01  0.323   0.7469
## Group18  1.332e+01  6.088e+05  7.381e+02  0.018   0.9856
## Group19 -1.426e+00  2.402e-01  7.655e-01 -1.863   0.0624 .
## Group20 -9.396e-01  3.908e-01  8.060e-01 -1.166   0.2437
## ---
## Signif. codes:  0 '***' 0.001 '**' 0.01 '*' 0.05 '.' 0.1 ' ' 1
##
##         exp(coef) exp(-coef) lower .95 upper .95
## Group1  8.354e-01  1.197e+00   0.66038     1.057
## Group2  8.143e-01  1.228e+00   0.55829     1.188
## Group3  7.003e-01  1.428e+00   0.45707     1.073
## Group4  6.718e-01  1.488e+00   0.43996     1.026
## Group5  1.129e+00  8.861e-01   0.74585     1.708
## Group6  8.802e-01  1.136e+00   0.66479     1.165
## Group7  7.412e-01  1.349e+00   0.18390     2.988
## Group8  4.876e-01  2.051e+00   0.12884     1.846
## Group9  1.386e+00  7.213e-01   0.77038     2.495
## Group10 1.196e+00  8.363e-01   0.60729     2.354
## Group11 1.197e+00  8.358e-01   0.50606     2.829
## Group12 4.096e-01  2.441e+00   0.15963     1.051
## Group13 4.476e-01  2.234e+00   0.04545     4.408
## Group14 8.334e-01  1.200e+00   0.27788     2.500
## Group15 1.338e+00  7.474e-01   0.29910     5.986
## Group16 2.096e+00  4.771e-01   0.58798     7.470
## Group17 1.198e+00  8.350e-01   0.40053     3.581
## Group18 6.088e+05  1.642e-06   0.00000       Inf
## Group19 2.402e-01  4.163e+00   0.05358     1.077
## Group20 3.908e-01  2.559e+00   0.08051     1.897
##
## Concordance= 0.529  (se = 0.01 )
## Likelihood ratio test= 27.56  on 20 df,   p=0.1
## Wald test            = 23.9  on 20 df,   p=0.2
## Score (logrank) test = 26.2  on 20 df,   p=0.2
```

```
beta <- fit1$coefficients
ciL <- confint(fit1)[,1]
ciU <- confint(fit1)[,2]
se <- summary(fit1)$coef[,3]

df_multiComp <- tibble(namen=c(c("Start->Hosp. WHF","Start->ACS","Start->Stroke",
                                 "Start->CV Death", "Start->Non CV Death",
                         "Hosp. WHF->Hosp. WHF","Hosp. WHF->ACS","Hosp. WHF->Stroke",
                         "Hosp. WHF->CV Death","Hosp. WHF->Non CV Death",
                         "ACS->ACS","ACS->Hosp. WHF","ACS->Stroke","ACS->CV Death",
                         "ACS->Non CV Death", "Stroke->Stroke","Stroke->Hosp. WHF",
                         "Stroke->ACS","Stroke->CV Death","Stroke->Non CV Death")),

             HR=beta,
             SE=se,
             CIL=ciL,
             CIU=ciU)

df_multiComp$HR[18] <- NA
df_multiComp$SE[18] <- NA
df_multiComp$CIL[18] <- NA
df_multiComp$CIU[18] <- NA
```

```
# Code for forest plot of effects
matGr <- datAllMulit[,9:28]
sumMatGr <- apply(matGr, 1, sum)
matEv <- cbind(datAllMulit$trans, datAllMulit$status, sumMatGr)
evs <- table(matEv[,1], matEv[,2], matEv[,3])

ggforestplot::forestplot(df=df_multiComp,name=namen, estimate=HR, se=SE, logodds = TRUE)+
  scale_x_continuous(limits = c(0.005,10), trans = "log10", breaks = c(0.1,0.5,1,2.5,5,10))+
  scale_y_discrete(expand = expansion(mult = c(0.05, .2)))+
 xlab(
   expression(
     atop("                                         HR",
                      paste(
                        atop
                        ("                                                         early rhythm control
                      "
                                                                         "%<-%"                ")",
                      " ",atop("usual care better",""%->%"")))))+
  theme(axis.text.y = element_text(hjust = 0))+
 annotate("text", x = rep(0.02,21), y = seq(21,1,-1),
           label = c("HR (95% CI)",paste0(formatC(exp(df_multiComp$HR),
                                                  format = "f",digits = 2),
                           " (",formatC(exp(df_multiComp$CIL),
                                        format = "f",digits = 2),",",
                                 formatC(exp(df_multiComp$CIU),
                                         format = "f",digits = 2),")")), size=2.5)+
  annotate("text", x = rep(0.0082,21), y = seq(21,1,-1),
           label = c("usual care",evs[,2,1]), size=2.5)+
   annotate("text", x = rep(0.0052,21),y = seq(21,1,-1),
            label = c("early rhythm control",evs[,2,2]), size=2.5)+
   annotate("text", x = rep(0.007,21),y = 22,
```

```
            label = c("Event nr."), size=2.5)
```

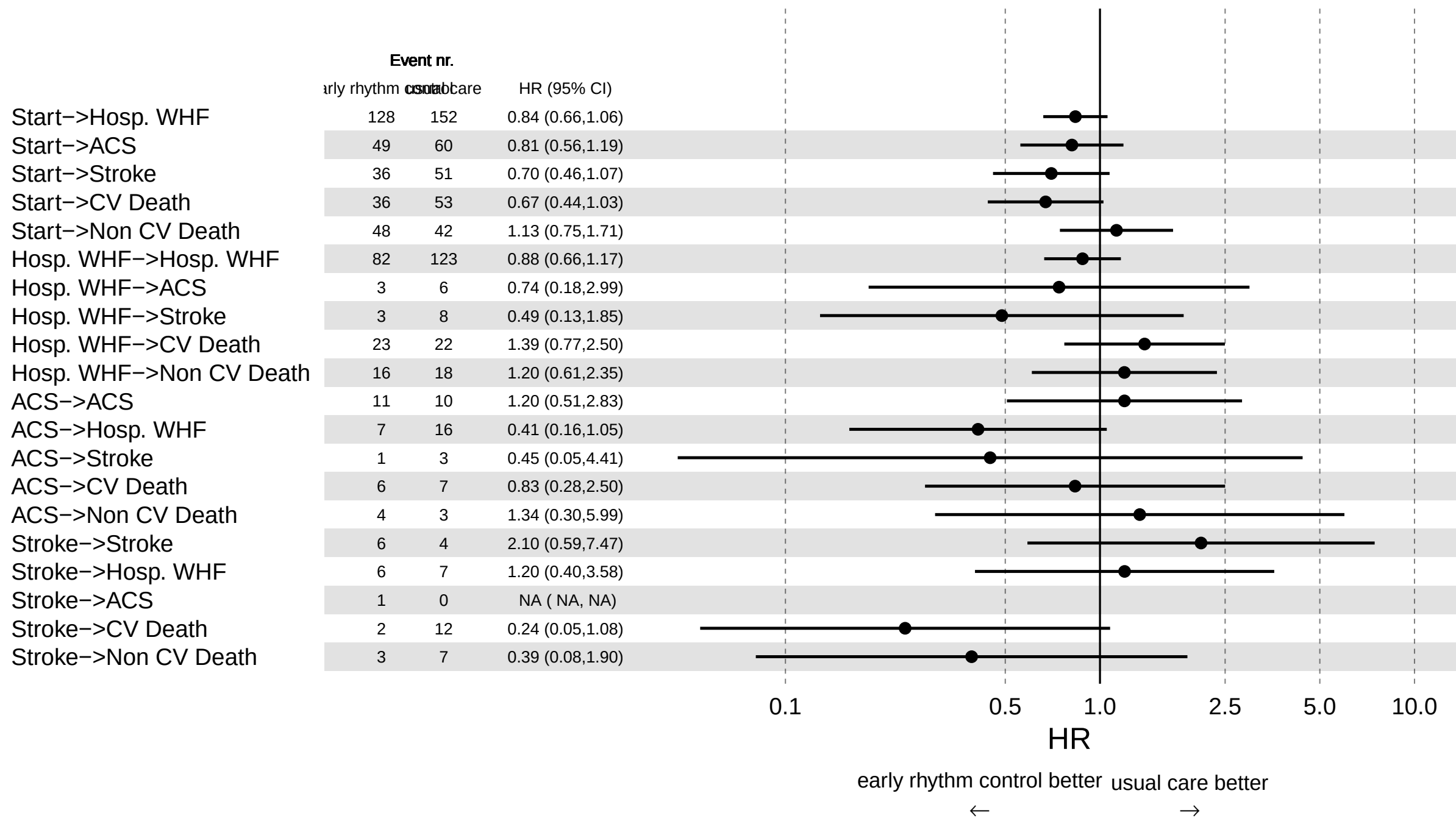


Figure 2: Multi-state model results

**Data structure for AUC and While-Alive approach**

| idx | arm | time | status | weights |
|---|---|---|---|---|
| 1 | 0 | 2505 | 1 | 1 |
| 1 | 0 | 3021 | 0 | 1 |
| 2 | 1 | 3133 | 0 | 1 |
| 3 | 1 | 1420 | 2 | 1 |
| 4 | 0 | 3132 | 0 | 1 |
| 5 | 0 | 1364 | 1 | 1 |

**AUC**

```
# AUC fit
aucs <- CompareAUCs(
  data=data_sel,
  alpha = 0.05,
  arm_name = "arm",
  idx_name = "idx",
  status_name = "status",
  time_name = "time"
)

# model output
show(aucs)
```

```
## Marginal Areas:
##   arm    n area   se  tau
## 1   0 1394  813 62.3 3132
## 2   1 1395  629 49.7 3132
##
##
## CIs:
##       method contrast observed      se    lower  upper
## 1 asymptotic    A1-A0 -184.000 79.7000 -340.000 -28.10
## 2 asymptotic    A1/A0    0.773  0.0851    0.623   0.96
##
##
## P-values:
##       method contrast observed      p
## 1 asymptotic    A1-A0 -184.000 0.0207
## 2 asymptotic    A1/A0    0.773 0.0195
```

```
# save effect plus CI for plot
aucRatio <- aucs@CIs$observed[2]
aucRatio_CIL <- aucs@CIs$lower[2]
aucRatio_CIU <- aucs@CIs$upper[2]

# Plot AUC
PlotMCFs(
  data_sel,
  arm_name = "arm",
  color_labs = c("usual care", "early rhythm control"),
  ctrl_color = "#56B4E9",
  idx_name = "idx",
```

```
  status_name = "status",
  time_name = "time",
  trt_color = "#E69F00",
  x_name = "Time [days]",
  y_name = "Mean Cumulative Count"
) + theme(axis.title = element_text(size=20),
          axis.text = element_text(size=15),
          legend.position = c(0.3,0.9),
          legend.text = element_text(size=15))
```

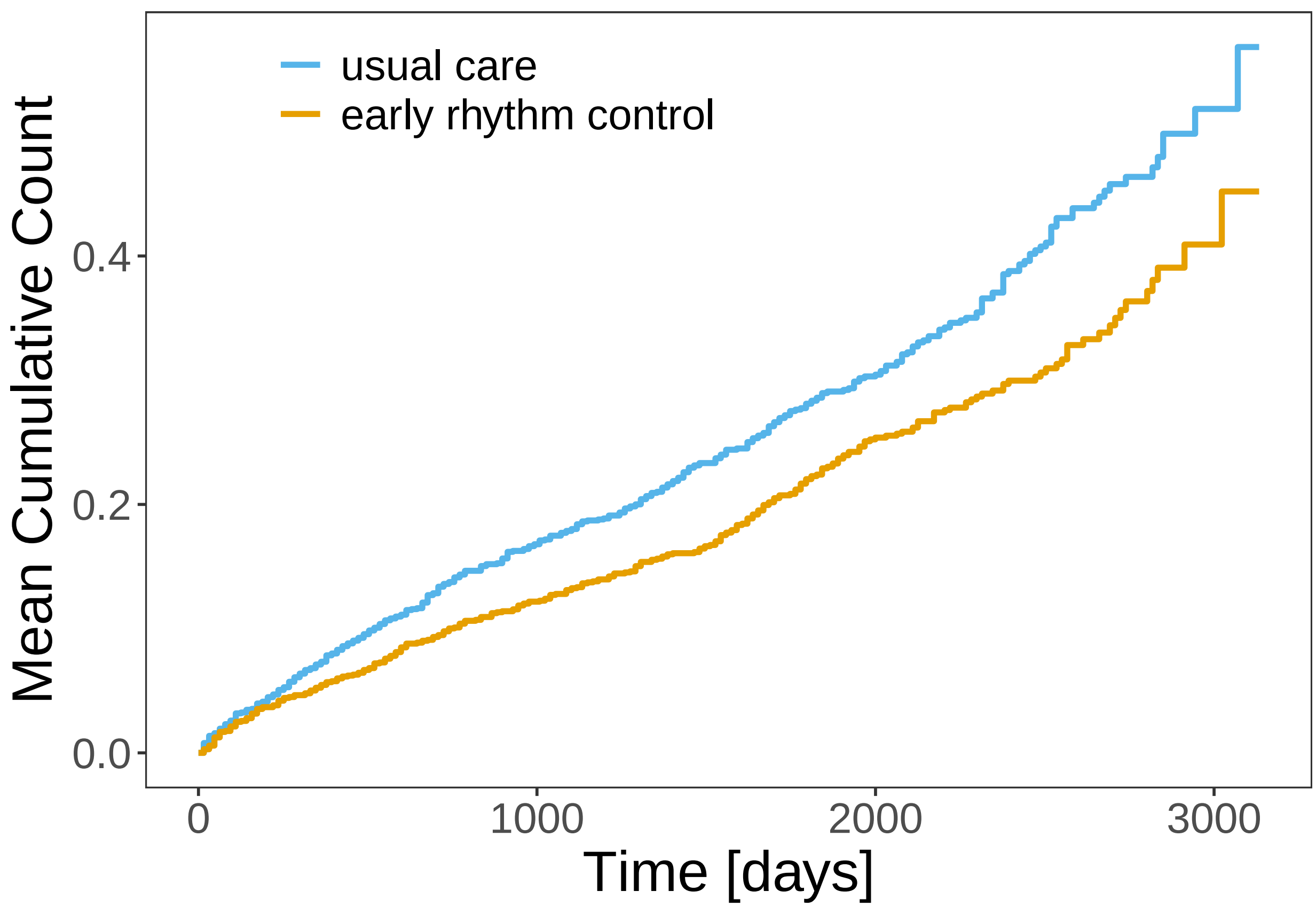


Figure 3: AUC

**While alive approach**

```
# datamanagement
data_sel <- data_sel %>%
  mutate(armOld=arm, arm=factor(arm, levels=c(0,1), labels=c("usual care","early rhythm control")))

# model fit
obj <- LRfit(data_sel$idx, data_sel$time, data_sel$status, data_sel$arm)

# save effect plus CI for plot
eff_WA <- exp(summary(obj,tau=max(data_sel$time),joint.test=T)$LRtab[2])
eff_WA_CIL <- exp(summary(obj,tau=max(data_sel$time),joint.test=T)$LRtab[2]-
                    1.96*summary(obj,tau=max(data_sel$time),joint.test=T)$LRtab[4])
```

```r
eff_WA_CIU <- exp(summary(obj,tau=max(data_sel$time),joint.test=T)$LRtab[2]+
                    1.96*summary(obj,tau=max(data_sel$time),joint.test=T)$LRtab[4])

# plot
pWA <- plot(obj,conf=T,xlab="Time [days]",xlim=c(0, 2000),ylim=c(0,0.5),
            group.col=c("#56B4E9", "#E69F00"))
```

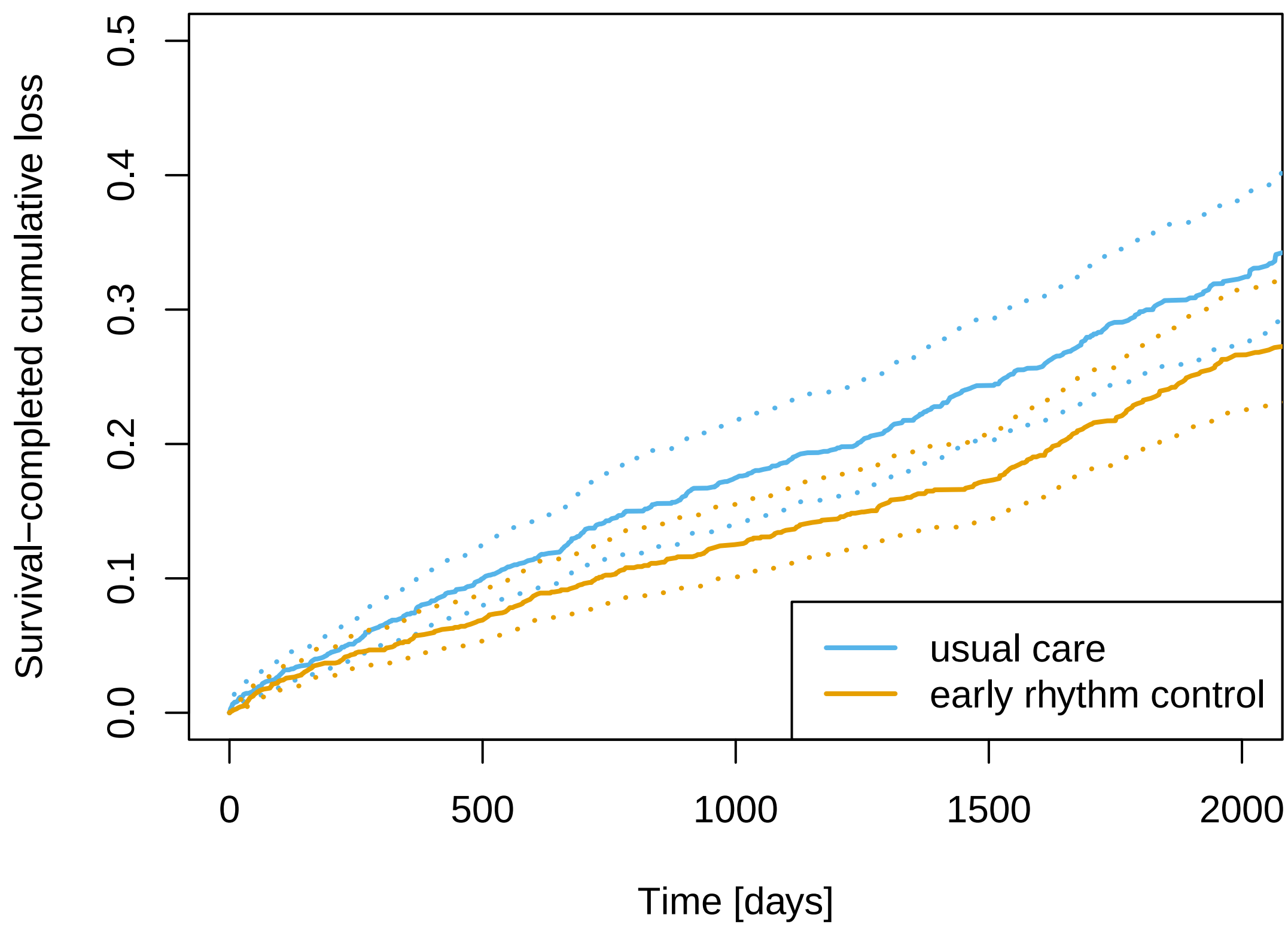


Figure 4: While-alive

## Further data description

### Data structure for Nelson-Aalen and Aalen-Johansen estimate (Version 1)

| id | entry | exit | to2 | from | to | Group |
|---|---|---|---|---|---|---|
| 1 | 0 | 2505 | 1 | start | compositeRec | 0 |
| 1 | 2505 | 3021 | cens | composite | cens | 0 |
| 2 | 0 | 3133 | cens | start | cens | 1 |
| 3 | 0 | 1420 | 1 | start | compositeRec | 1 |
| 4 | 0 | 3132 | cens | start | cens | 0 |
| 5 | 0 | 1364 | 1 | start | compositeRec | 0 |

### Nelson Aalen

```
# Nelson Aalen estimates
na1 <- mvna(testall4 %>% filter(Group==0),c("start","composite","compositeRec",
                                            "nonCVdeath"),tra,"cens")
na2 <- mvna(testall4 %>% filter(Group==1),c("start","composite","compositeRec",
                                            "nonCVdeath"),tra,"cens")

# plots
gglist <- list()

gglist[[1]] <- ggplot() + geom_step(data=na1[[1]] %>% mutate(gr1="usual care"),
                                    aes(x=time, y=na, color=gr1), size=1.1) +
  geom_step(data=na2[[1]] %>% mutate(gr1="early rhythm control"),
            aes(x=time, y=na, color=gr1), size=1.1) +
  scale_x_continuous(limits = c(0,3200)) +
  scale_color_manual(values = c("#E69F00","#56B4E9"))+
  ggtitle("    Nelson-Aalen estimate for transition between
           start and event of composite endpoint      ")+
  xlab("Time [days]") +
  ylab("Nelson-Aalen estimate")+
  theme_bw() +
  theme(panel.grid = element_blank(),
        legend.title = element_blank(),
        legend.position = c(0.1,0.85),
        plot.title = element_text(size=8, face="bold", hjust=0.5),
        axis.title = element_text(size=8),
         axis.text = element_text(size=8, color="black"))


gglist[[2]] <- ggplot() + geom_step(data=na1[[3]] %>% mutate(gr1="usual care"),
                                    aes(x=time, y=na, color=gr1), size=1.1) +
  geom_step(data=na2[[3]] %>% mutate(gr1="early rhythm control"),
            aes(x=time, y=na, color=gr1), size=1.1) +
  scale_x_continuous(limits = c(0,3200)) +
  scale_color_manual(values = c("#E69F00","#56B4E9"))+
  ggtitle("   Nelson-Aalen estimate for transition between event of
      composite and recurrent event of composite endpoint   ")+
  xlab("Time [days]") +
  ylab("Nelson-Aalen estimate")+
  theme_bw() +
```

```r
  theme(panel.grid = element_blank(),
        legend.title = element_blank(),
        legend.position = c(0.1,0.85),
        plot.title = element_text(size=8, face="bold", hjust = 0.5),
        axis.title = element_text(size=8),
         axis.text = element_text(size=8, color="black"))


gg <- ggarrange(gglist[[1]], gglist[[2]],
          ncol=2, common.legend = TRUE, legend = "bottom")

gg
```

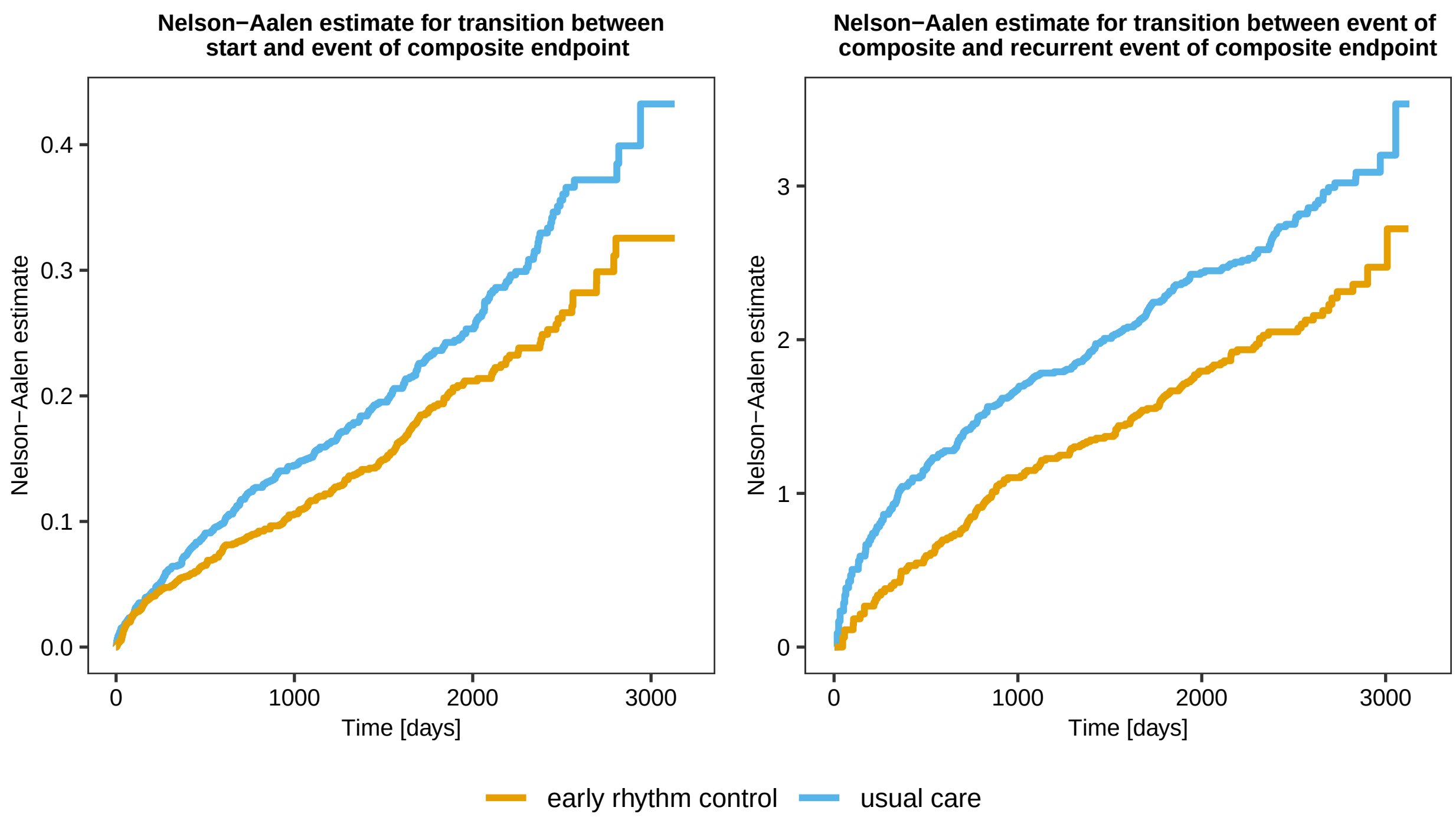


Figure 5: Nelson-Aalen

**Aalen Johansen**

```
# Aalen Johansen estimates
AJ1<-etm(testall4 %>% filter(Group==0),c("start","composite","compositeRec",
                                          "nonCVdeath"),tra,"cens",s=0)

AJ2<-etm(testall4 %>% filter(Group==1),c("start","composite","compositeRec",
                                          "nonCVdeath"),tra,"cens",s=0)

layout.matrix <- matrix(c(1, 2, 3, 0), nrow = 2, ncol = 2, byrow = TRUE)

layout(mat = layout.matrix,
       heights = c(2, 0.25), # Heights of the two rows
       widths = c(2, 2)) # Widths of the two columns

plot(AJ1, tr.choice=c("start compositeRec"), conf.int=FALSE, col="#56B4E9",
     main="Aalen-Johansen estimate for transition between
     start and event of composite endpoint",
     legend = FALSE,
     xlim= c(0,3200), ylim=c(0,1),ylab="Aalen-Johansen estimate",
     xlab="Time [days]",
     lwd=2, lty=1,cex.main=1)
lines(AJ2, tr.choice=c("start compositeRec"),
      conf.int=FALSE, lwd=2, col="#E69F00", lty=1)

plot(AJ1, tr.choice=c("composite compositeRec"), conf.int=FALSE, col="#56B4E9",
     main="Aalen-Johansen estimate for transition between
     event of composite and recurrent event of composite endpoint",
     legend = FALSE,
     xlim= c(0,3200), ylim=c(0,1),ylab="Aalen-Johansen estimate",
     xlab="Time [days]",
     lwd=2, lty=1,cex.main=1)
lines(AJ2, tr.choice=c("composite compositeRec"),
      conf.int=FALSE, lwd=2, col="#E69F00", lty=1)

par(mar=c(0,0,0,0)) # Make the margins 0
plot(1, type = "n", axes=F, xlab="", ylab="") # Create empty plot
legend("top", c("usual care", "early rhythm control"), horiz=TRUE,
lty=c(1,1), col=c("#56B4E9", "#E69F00"))
```

```
## pdf
##   2
```

**Data structure for Nelson-Aalen and Aalen-Johansen estimate (Version 2)**

| id | entry | exit | to2 | from | to | Group |
|---|---|---|---|---|---|---|
| 1 | 0 | 2505 | 2 | start | recWHF2 | 0 |
| 1 | 2505 | 3021 | cens | recWHF | cens | 0 |
| 2 | 0 | 3133 | cens | start | cens | 1 |
| 3 | 0 | 1420 | 4 | start | cvd | 1 |
| 4 | 0 | 3132 | cens | start | cens | 0 |
| 5 | 0 | 1364 | 3 | start | recStroke2 | 0 |

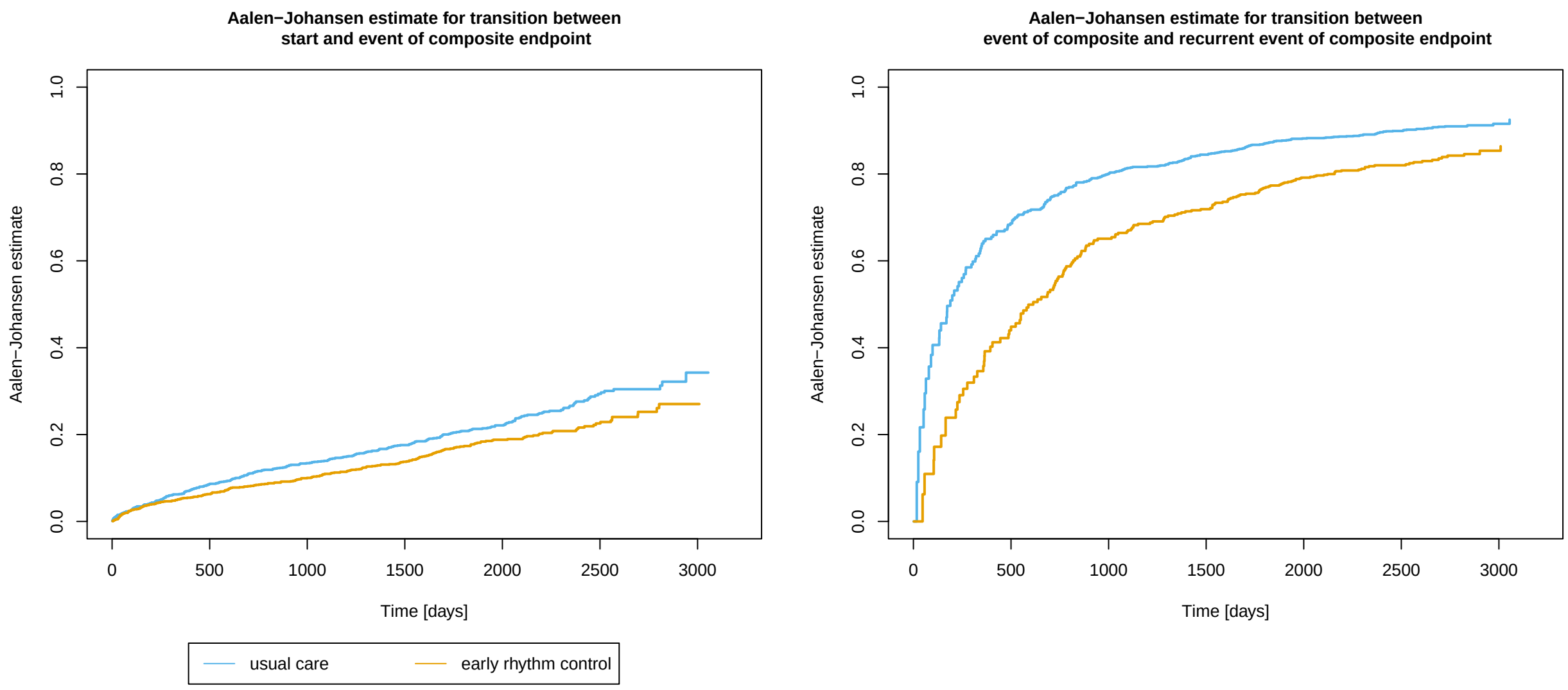


Figure 6: Aalen-Johansen

### Nelson Aalen

```
# Nelson Aalen estimates
na1 <- mvna(testall4 %>% filter(Group==0),c("start","recWHF","recACS","recStroke",
                                            "recWHF2","recACS2","recStroke2","cvd",
                                            "ncvd"),tra,"cens")
na2 <- mvna(testall4 %>% filter(Group==1),c("start","recWHF","recACS","recStroke",
                                            "recWHF2","recACS2","recStroke2","cvd",
                                            "ncvd"),tra,"cens")

# plots
gglist <- list()

for(i in 1:20){
gglist[[i]] <- ggplot() + geom_step(data=na1[[i]] %>% mutate(gr1="usual care"),
                                    aes(x=time, y=na, color=gr1), size=1.1) +
  geom_step(data=na2[[i]]%>% mutate(gr1="early rhythm control"),aes(x=time, y=na,
                                                                    color=gr1), size=1.1) +
  scale_x_continuous(limits = c(0,3200)) +
  #scale_y_continuous(limits=(c(0, 0.3))) +
  scale_color_manual(values = c("#E69F00","#56B4E9"))+
  ggtitle(paste0("Nelson-Aalen estimate for\ntransition: " ,names(na1)[i]))+
  xlab("Time [days]") +
  ylab("Nelson-Aalen estimate")+
  theme_bw() +
  theme(panel.grid = element_blank(),
        legend.title = element_blank(),
        legend.position = c(0.1,0.85),
        plot.title = element_text(size=8, face="bold"),
        axis.title = element_text(size=8),
         axis.text = element_text(size=8, color="black"))
}
```

```r
gg <- ggarrange(gglist[[1]], gglist[[2]], gglist[[3]], gglist[[4]], gglist[[5]], gglist[[6]],
                gglist[[7]], gglist[[8]], gglist[[9]],
          gglist[[10]], gglist[[11]], gglist[[12]], gglist[[13]], gglist[[14]], gglist[[15]],
          gglist[[16]], gglist[[17]], gglist[[18]], gglist[[19]], gglist[[20]],
          nrow=4, ncol=5, common.legend = TRUE, legend = "bottom")

gg
```

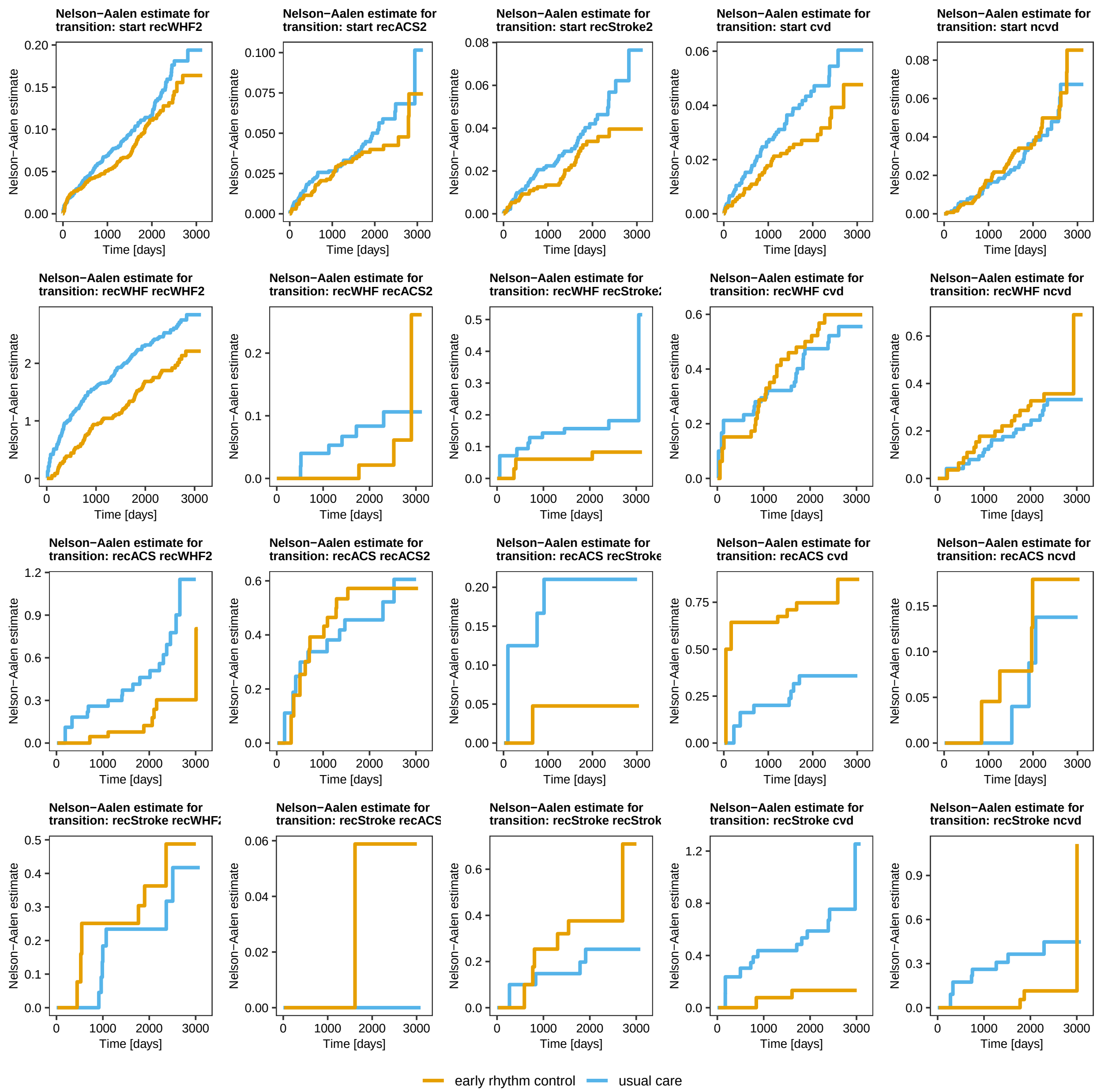


Figure 7: Nelson-Aalen

**Aalen Johansen**

```
# Aalen Johansen estimates
AJ1<-etm(testall4 %>% filter(Group==0),c("start","recWHF","recACS","recStroke",
                                          "recWHF2","recACS2","recStroke2","cvd",
                                          "ncvd"),tra,"cens",s=0)

AJ2<-etm(testall4 %>% filter(Group==1),c("start","recWHF","recACS","recStroke",
                                          "recWHF2","recACS2","recStroke2","cvd",
                                          "ncvd"),tra,"cens",s=0)

# plot
par(mfrow = c(4,5))
plot(AJ1, tr.choice=c("start recWHF2"), conf.int=FALSE, col="#56B4E9",
     main="Aalen-Johansen estimate for\n transition start recWHF", legend = FALSE,
     xlim= c(0,3200), ylim=c(0,0.5),ylab="Aalen-Johansen estimate", lwd=2, lty=1,cex.main=1)
lines(AJ2, tr.choice=c("start recWHF2"), conf.int=FALSE, lwd=2, col="#E69F00", lty=1)

plot(AJ1, tr.choice=c("start recACS2"), conf.int=FALSE, col="#56B4E9",
     main="Aalen-Johansen estimate for\n transition start recACS", legend = FALSE,
     xlim= c(0,3200), ylim=c(0,0.5),ylab="Aalen-Johansen estimate", lwd=2, lty=1,cex.main=1)
lines(AJ2, tr.choice=c("start recACS2"), conf.int=FALSE, lwd=2, col="#E69F00", lty=1)

plot(AJ1, tr.choice=c("start recStroke2"), conf.int=FALSE, col="#56B4E9",
     main="Aalen-Johansen estimate for\n transition start recStroke", legend = FALSE,
     xlim= c(0,3200), ylim=c(0,0.5),ylab="Aalen-Johansen estimate", lwd=2, lty=1,cex.main=1)
lines(AJ2, tr.choice=c("start recStroke2"), conf.int=FALSE, lwd=2, col="#E69F00", lty=1)

plot(AJ1, tr.choice=c("start cvd"), conf.int=FALSE, col="#56B4E9",
     main="Aalen-Johansen estimate for\n transition start cvd", legend = FALSE,
     xlim= c(0,3200), ylim=c(0,0.5),ylab="Aalen-Johansen estimate", lwd=2, lty=1,cex.main=1)
lines(AJ2, tr.choice=c("start cvd"), conf.int=FALSE, lwd=2, col="#E69F00", lty=1)

plot(AJ1, tr.choice=c("start ncvd"), conf.int=FALSE, col="#56B4E9",
     main="Aalen-Johansen estimate for\n transition start ncvd", legend = FALSE,
     xlim= c(0,3200), ylim=c(0,0.5),ylab="Aalen-Johansen estimate", lwd=2, lty=1,cex.main=1)
lines(AJ2, tr.choice=c("start ncvd"), conf.int=FALSE, lwd=2, col="#E69F00", lty=1)

plot(AJ1, tr.choice=c("recWHF recWHF2"), conf.int=FALSE, col="#56B4E9",
     main="Aalen-Johansen estimate for\n transition recWHF recWHF", legend = FALSE,
     xlim= c(0,3200),ylab="Aalen-Johansen estimate", lwd=2, lty=1,cex.main=1)
lines(AJ2, tr.choice=c("recWHF recWHF2"), conf.int=FALSE, lwd=2, col="#E69F00", lty=1)

plot(AJ1, tr.choice=c("recWHF recACS2"), conf.int=FALSE, col="#56B4E9",
     main="Aalen-Johansen estimate for\n transition recWHF recACS", legend = FALSE,
     xlim= c(0,3200),ylab="Aalen-Johansen estimate", lwd=2, lty=1,cex.main=1)
lines(AJ2, tr.choice=c("recWHF recACS2"), conf.int=FALSE, lwd=2, col="#E69F00", lty=1)

plot(AJ1, tr.choice=c("recWHF recStroke2"), conf.int=FALSE, col="#56B4E9",
     main="Aalen-Johansen estimate for\n transition recWHF recStroke", legend = FALSE,
     xlim= c(0,3200),ylab="Aalen-Johansen estimate", lwd=2, lty=1,cex.main=1)
lines(AJ2, tr.choice=c("recWHF recStroke2"), conf.int=FALSE, lwd=2, col="#E69F00", lty=1)

plot(AJ1, tr.choice=c("recWHF cvd"), conf.int=FALSE, col="#56B4E9",
```

```r
     main="Aalen-Johansen estimate for\n transition recWHF cvd", legend = FALSE,
     xlim= c(0,3200),ylab="Aalen-Johansen estimate", lwd=2, lty=1,cex.main=1)
lines(AJ2, tr.choice=c("recWHF cvd"), conf.int=FALSE, lwd=2, col="#E69F00", lty=1)

plot(AJ1, tr.choice=c("recWHF ncvd"), conf.int=FALSE, col="#56B4E9",
     main="Aalen-Johansen estimate for\n transition recWHF ncvd", legend = FALSE,
     xlim= c(0,3200),ylab="Aalen-Johansen estimate", lwd=2, lty=1,cex.main=1)
lines(AJ2, tr.choice=c("recWHF ncvd"), conf.int=FALSE, lwd=2, col="#E69F00", lty=1)

plot(AJ1, tr.choice=c("recACS recWHF2"), conf.int=FALSE, col="#56B4E9",
     main="Aalen-Johansen estimate for\n transition recACS recWHF", legend = FALSE,
     xlim= c(0,3200),ylab="Aalen-Johansen estimate", lwd=2, lty=1,cex.main=1)
lines(AJ2, tr.choice=c("recACS recWHF2"), conf.int=FALSE, lwd=2, col="#E69F00", lty=1)

plot(AJ1, tr.choice=c("recACS recACS2"), conf.int=FALSE, col="#56B4E9",
     main="Aalen-Johansen estimate for\n transition recACS recACS", legend = FALSE,
     xlim= c(0,3200),ylab="Aalen-Johansen estimate", lwd=2, lty=1,cex.main=1)
lines(AJ2, tr.choice=c("recACS recACS2"), conf.int=FALSE, lwd=2, col="#E69F00", lty=1)

plot(AJ1, tr.choice=c("recACS recStroke2"), conf.int=FALSE, col="#56B4E9",
     main="Aalen-Johansen estimate for\n transition recACS recStroke", legend = FALSE,
     xlim= c(0,3200),ylab="Aalen-Johansen estimate", lwd=2, lty=1,cex.main=1)
lines(AJ2, tr.choice=c("recACS recStroke2"), conf.int=FALSE, lwd=2, col="#E69F00", lty=1)

plot(AJ1, tr.choice=c("recACS cvd"), conf.int=FALSE, col="#56B4E9",
     main="Aalen-Johansen estimate for\n transition recACS cvd", legend = FALSE,
     xlim= c(0,3200),ylab="Aalen-Johansen estimate", lwd=2, lty=1,cex.main=1)
lines(AJ2, tr.choice=c("recACS cvd"), conf.int=FALSE, lwd=2, col="#E69F00", lty=1)

plot(AJ1, tr.choice=c("recACS ncvd"), conf.int=FALSE, col="#56B4E9",
     main="Aalen-Johansen estimate for\n transition recACS ncvd", legend = FALSE,
     xlim= c(0,3200),ylab="Aalen-Johansen estimate", lwd=2, lty=1,cex.main=1)
lines(AJ2, tr.choice=c("recACS ncvd"), conf.int=FALSE, lwd=2, col="#E69F00", lty=1)

plot(AJ1, tr.choice=c("recStroke recWHF2"), conf.int=FALSE, col="#56B4E9",
     main="Aalen-Johansen estimate for\n transition recStroke recWHF", legend = FALSE,
     xlim= c(0,3200),ylab="Aalen-Johansen estimate", lwd=2, lty=1,cex.main=1)
lines(AJ2, tr.choice=c("recStroke recWHF2"), conf.int=FALSE, lwd=2, col="#E69F00", lty=1)

plot(AJ1, tr.choice=c("recStroke recACS2"), conf.int=FALSE, col="#56B4E9",
     main="Aalen-Johansen estimate for\n transition recStroke recACS", legend = FALSE,
     xlim= c(0,3200),ylab="Aalen-Johansen estimate", lwd=2, lty=1,cex.main=1)
lines(AJ2, tr.choice=c("recStroke recACS2"), conf.int=FALSE, lwd=2, col="#E69F00", lty=1)

plot(AJ1, tr.choice=c("recStroke recStroke2"), conf.int=FALSE, col="#56B4E9",
     main="Aalen-Johansen estimate for\n transition recStroke recStroke", legend = FALSE,
     xlim= c(0,3200),ylab="Aalen-Johansen estimate", lwd=2, lty=1,cex.main=1)
lines(AJ2, tr.choice=c("recStroke recStroke2"), conf.int=FALSE, lwd=2, col="#E69F00", lty=1)

plot(AJ1, tr.choice=c("recStroke cvd"), conf.int=FALSE, col="#56B4E9",
     main="Aalen-Johansen estimate for\n transition recStroke cvd", legend = FALSE,
     xlim= c(0,3200),ylab="Aalen-Johansen estimate", lwd=2, lty=1,cex.main=1)
lines(AJ2, tr.choice=c("recStroke cvd"), conf.int=FALSE, lwd=2, col="#E69F00", lty=1)
```

```r
plot(AJ1, tr.choice=c("recStroke ncvd"), conf.int=FALSE, col="#56B4E9",
     main="Aalen-Johansen estimate for\n transition recStroke ncvd", legend = FALSE,
     xlim= c(0,3200),ylab="Aalen-Johansen estimate", lwd=2, lty=1,cex.main=1)
lines(AJ2, tr.choice=c("recStroke ncvd"), conf.int=FALSE, lwd=2, col="#E69F00", lty=1)
```

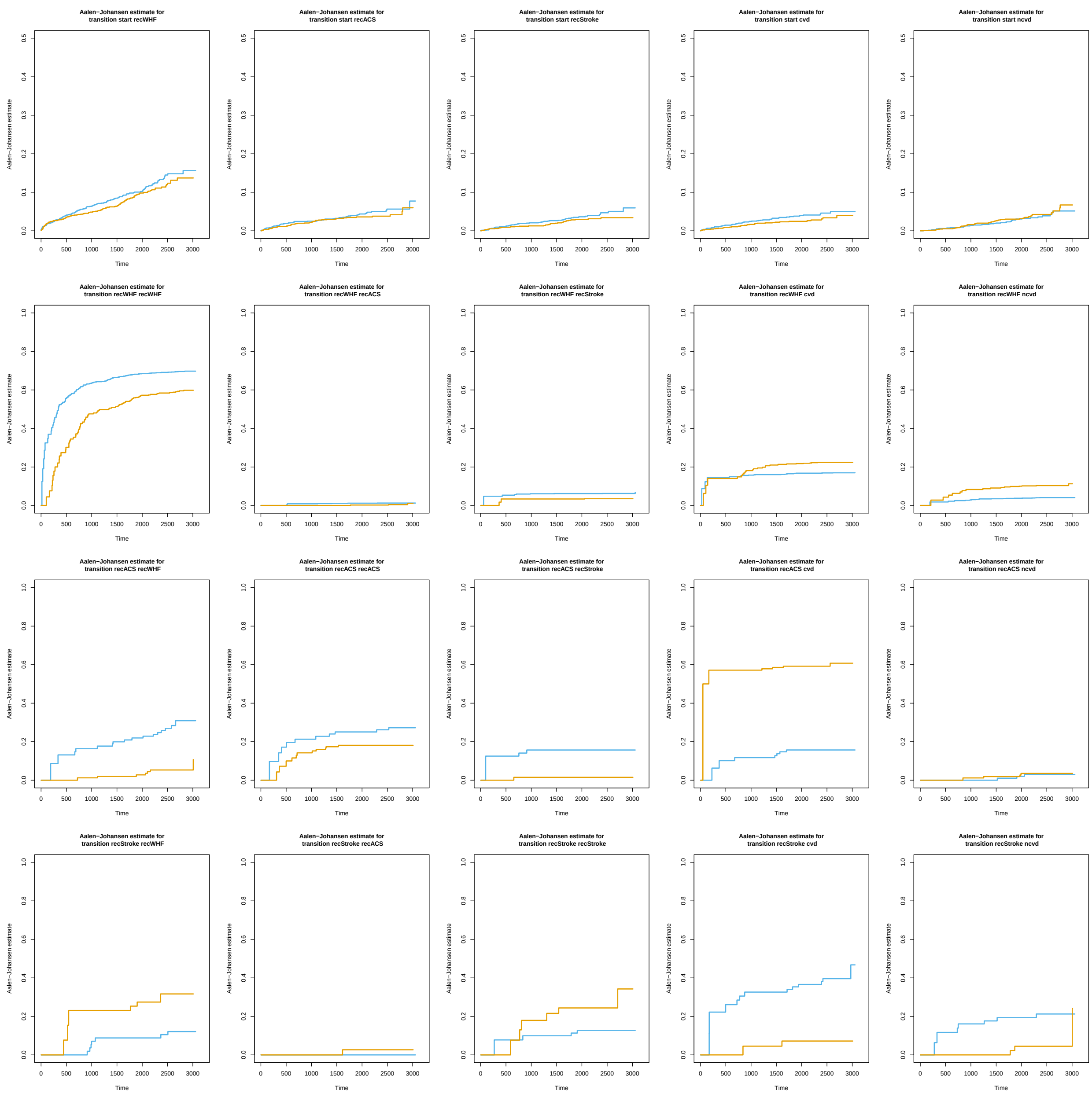


Figure 8: Aalen-Johansen

### Figures to compare effect estimates

```
data_all_eff <- tibble(Model=c("AG","LWYY","PWP","WLW","NB"),
                       ratio=c(exp(eff_AG$estimate),exp(eff_LWYY$estimate),
                               exp(eff_PWP$estimate),exp(eff_WLW$estimate),
                               eff_NB$ratio),
                       CIL=c(exp(eff_AG$asymp.LCL),exp(eff_LWYY$asymp.LCL),
                             exp(eff_PWP$asymp.LCL),exp(eff_WLW$asymp.LCL),
                             eff_NB$asymp.LCL),
                       CIU=c(exp(eff_AG$asymp.UCL),exp(eff_LWYY$asymp.UCL),
                             exp(eff_PWP$asymp.UCL),exp(eff_WLW$asymp.UCL),
                             eff_NB$asymp.UCL)) %>%
  mutate(` `=paste(rep("       ", 5), collapse = " "),"Ratio (95%CI)"=
           paste0(formatC(ratio, digits=2, format="f"),
                  " (",formatC(CIL, digits=2, format="f"),", ",
                  formatC(CIU, digits=2, format="f"),")")) %>%
  dplyr::select(Model,` `,everything())



# Define a custom forest theme with adjusted column spacing
theme_forest <- forest_theme(
    base_size = 10,
    refline_col = "red",
    box_col = "blue",
    line_col = "black",
    summary_fill = "gray", colgap = unit(5, "mm"),
    vertline_width = 1.2
)


# Generate the forest plot
g0 <- forest(
    data = data_all_eff[,-c(3:5)],
    est = data_all_eff$ratio,
    lower = data_all_eff$CIL,
    upper = data_all_eff$CIU,
    x_trans = "log",
    ref_line = 1,
    xlim = c(0.4, 1.51),
    ticks_at = c(0.5,0.75, 1,1.5),
    ci_column = 2,
    fontsize = 10,
    arrow_lab = c("early rhythm control better", "usual care better"),
    align = c("l", "c", "r", "r"),
    title = "Composite\n(clinical relevance not considered)\n(semi)-parametric models\n",
    theme = theme_forest
)
```

```
data_all_eff <- tibble("Model\n"=c("param.","stratified param."),
                       AF=c(1/p_AG, 1/p_PWP),
                       CIU=c(1/p_AG_CIL, 1/p_PWP_CIL),
                       CIL=c(1/p_AG_CIU, 1/p_PWP_CIU)) %>%
  mutate(` `=paste(rep("    ", 5), collapse = " "),"1/AF (95%CI)"=
```

```
           paste0(formatC(AF, digits=2, format="f"),
                  " (",formatC(CIL, digits=2, format="f"),", ",
                  formatC(CIU, digits=2, format="f"),")")) %>%
  dplyr::select(`Model\n`,` `,everything())


# Generate the forest plot
g1 <- forest(
    data = data_all_eff[,-c(3:5)],
    est = data_all_eff$AF,
    lower = data_all_eff$CIL,
    upper = data_all_eff$CIU,
    x_trans = "log",
    ref_line = 1,
    xlim = c(0.5, 2),
    ticks_at = c(0.5,0.75,1,1.5),
    ci_column = 2,
    fontsize = 10,
    arrow_lab = c("early rhythm control better\n", "usual care better\n"),
    align = c("l", "c", "r", "r"),
    title = "Composite\n(clinical relevance not considered)\nparametric models\n",
    theme = theme_forest
)
```

```
data_all_eff <- tibble(Model=c("weighted LWYY","WHR param.",
                              #"WHR stratified param.",
                               "WHR non param.",
                              "WL","stratified WL"),
                       ratio=c(eff_LWYYweight,WHR_p,
                              # WHR_p2,
                               WHR,hrWL2,hrWL),
                       CIL=c(
                             eff_LWYYweightlCU,ciHRW_p[1],
                           # ciHRW_p2[1],
                             ciHRW[1],hrWL_CIL2,hrWL_CIL),
                       CIU=c(
                             eff_LWYYweightuCU, ciHRW_p[2],
                             #ciHRW_p2[2],
                             ciHRW[2],hrWL_CIU2,hrWL_CIU
                           )) %>%
  mutate(` `=paste(rep("      ", 3), collapse = " "),"Ratio (95%CI)"=
           paste0(formatC(ratio, digits=2, format="f"),
                  " (",formatC(CIL, digits=2, format="f"),", ",
                  formatC(CIU, digits=2, format="f"),")")) %>%
  dplyr::select(Model,` `,everything())



g2 <- forest(
    data = data_all_eff[,-c(3:5)],
    est = data_all_eff$ratio,
    lower = data_all_eff$CIL,
    upper = data_all_eff$CIU,
    x_trans = "log",
```

```
    ref_line = 1,
    xlim = c(0.4, 1.51),
    ticks_at = c(0.5,0.75, 1,1.5),
    ci_column = 2,
    fontsize = 10,
    arrow_lab = c("early rhythm control better", "usual care better"),
    align = c("l", "c", "r", "r"),
    title = "Composite\n(clinical relevance considered\nvia weights)\n",
    theme = theme_forest
)
```

```
data_all_eff <- tibble("Model\n"=c("1/win ratio","1/win odds"),
                       ratio=c(WR,WO),
                       CIL=c(WR_CIU,WO_CIU),
                       CIU=c(WR_CIL,WO_CIL)) %>%
  mutate(` `=paste(rep("     ", 5), collapse = " "),"Ratio (95%CI)"=
           paste0(formatC(ratio, digits=2, format="f"),
                  " (",formatC(CIL, digits=2, format="f"),", ",
                  formatC(CIU, digits=2, format="f"),")")) %>%
  dplyr::select(`Model\n`,` `,everything())



g3 <- forest(
    data = data_all_eff[,-c(3:5)],
    est = data_all_eff$ratio,
    lower = data_all_eff$CIL,
    upper = data_all_eff$CIU,
    x_trans = "log",
    ref_line = 1,
    xlim = c(0.4, 1.51),
    ticks_at = c(0.5,0.75, 1,1.5),
    ci_column = 2,
    fontsize = 10,
    arrow_lab = c("early rhythm control better\n", "usual care better\n"),
    align = c("l", "c", "r", "r"),
    title = "Composite\n(clinical relevance considered\nvia ordered endpoints)\n",
    theme = theme_forest
)
```

```
data_all_eff <- tibble("Model\n"=c("semi-param. JF","param. JF"),
                       ratio=c(exp(effJF[1]),exp(effJFp[1])),
                       CIL=c(exp(ciJFL[1]),exp(ciJFLp[1])),
                       CIU=c(exp(ciJFU[1]),exp(ciJFUp[1]))) %>%
  mutate(` `=paste(rep("    ", 4), collapse = " "),"Ratio (95%CI)"=
           paste0(formatC(ratio, digits=2, format="f"),
                  " (",formatC(CIL, digits=2, format="f"),", ",
                  formatC(CIU, digits=2, format="f"),")")) %>%
  dplyr::select(`Model\n`,` `,everything())



g4 <- forest(
    data = data_all_eff[,-c(3:5)],
```

```
    est = data_all_eff$ratio,
    lower = data_all_eff$CIL,
    upper = data_all_eff$CIU,
    x_trans = "log",
    ref_line = 1,
    xlim = c(0.4, 1.51),
    ticks_at = c(0.5,0.75, 1,1.5),
    ci_column = 2,
    fontsize = 10,
    arrow_lab = c("early rhythm control better\n", "usual care better\n"),
    align = c("l", "c", "r", "r"),
    title = "Composite\n(with correlated competing event)\n",
    theme = theme_forest
)
```

```
data_all_eff <- tibble("Model\n"=c("AUC","while-alive"),
                       ratio=c(aucRatio,eff_WA),
                       CIL=c(aucRatio_CIL,eff_WA_CIL),
                       CIU=c(aucRatio_CIU,eff_WA_CIU)) %>%
  mutate(` `=paste(rep("     ", 5), collapse = " "),"Ratio (95%CI)"=
           paste0(formatC(ratio, digits=2, format="f"),
                  " (",formatC(CIL, digits=2, format="f"),", ",
                  formatC(CIU, digits=2, format="f"),")")) %>%
  dplyr::select(`Model\n`,` `,everything())



g5 <- forest(
    data = data_all_eff[,-c(3:5)],
    est = data_all_eff$ratio,
    lower = data_all_eff$CIL,
    upper = data_all_eff$CIU,
    x_trans = "log",
    ref_line = 1,
    xlim = c(0.4, 1.51),
    ticks_at = c(0.5,0.75, 1,1.5),
    ci_column = 2,
    fontsize = 10,
    arrow_lab = c("early rhythm control better\n", "usual care better\n"),
    align = c("l", "c", "r", "r"),
    title = "Composite\n(terminated by death)\n",
    theme = theme_forest
)
```

```
plot1 <- plot_grid(g0, g1, g2, g3 , g4, g5,
                   labels = c("A","B", "C","D","E","F"),
                   align = "hv", axis = "tblr", nrow = 3 )


title_theme <- ggdraw() +
  draw_label("Analysis results for EAST-AFNET 4, the example study", hjust = 0, x=0.05,
             fontface = "bold")

plot_grid(title_theme, plot1, ncol=1,rel_heights = c(0.1,1))
```

Analysis results for EAST−AFNET 4, the example study

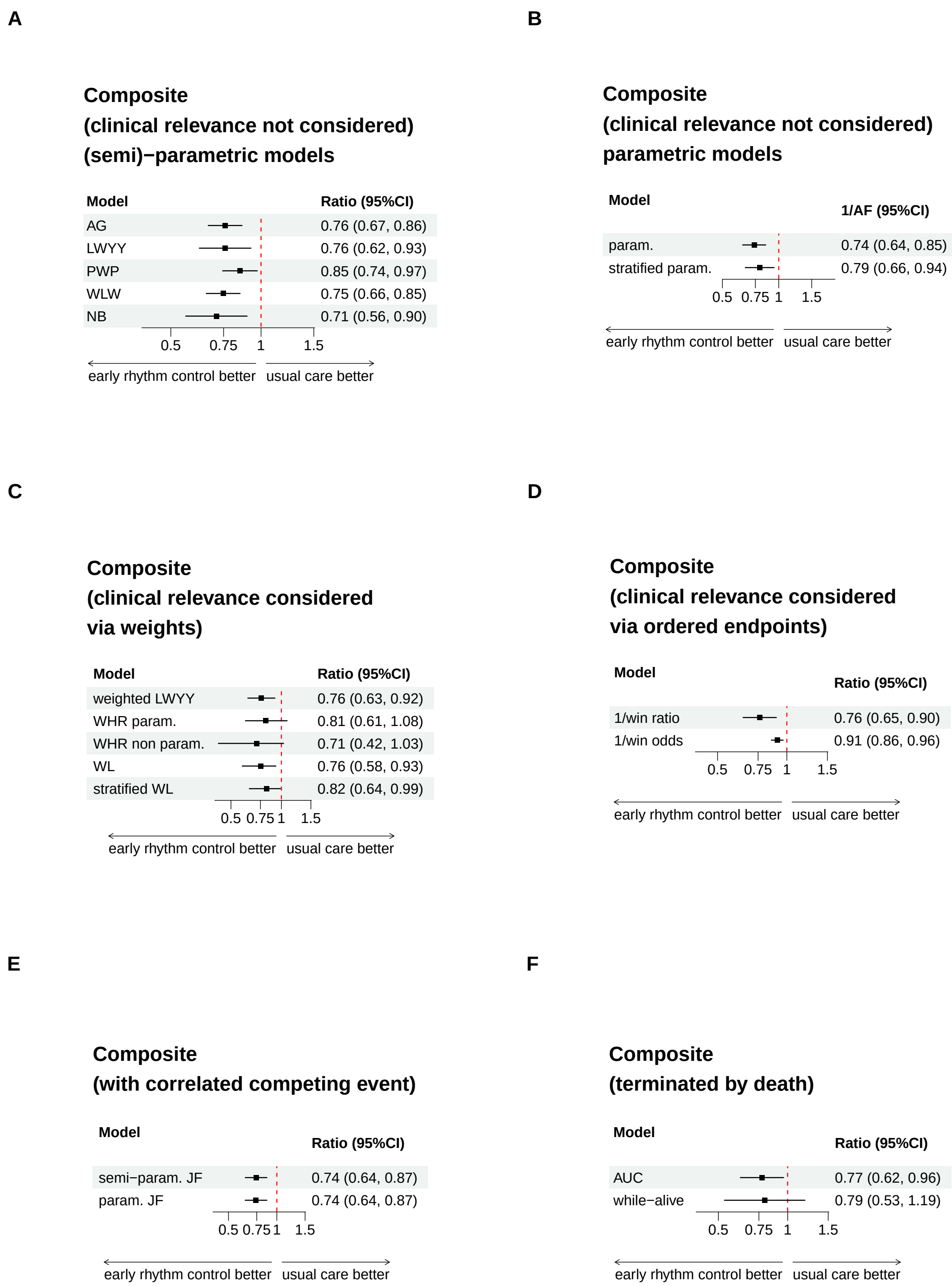

## Software environment

```
pander::pander(sessionInfo(), compact = TRUE)
```

**R version 4.4.2 (2024-10-31 ucrt)**

**Platform:** x86_64-w64-mingw32/x64

**locale:** *LC_COLLATE=German_Germany.utf8*, *LC_CTYPE=German_Germany.utf8*, *LC_MONETARY=German_Germa*
*LC_NUMERIC=C* and *LC_TIME=German_Germany.utf8*

**attached base packages:** *stats*, *graphics*, *grDevices*, *utils*, *datasets*, *methods* and *base*

**other attached packages:** *cowplot(v.1.2.0)*, *ggpubr(v.0.6.2)*, *forestploter(v.1.1.3)*, *emmeans(v.2.0.0)*, *WA(v.1.0)*, *MCC(v.0.7.1)*, *BuyseTest(v.3.3.4)*, *Rcpp(v.1.1.0)*, *Wcompo(v.1.0)*, *etm(v.1.1.2)*, *mvna(v.2.0.1)*, *flexsurv(v.2.3.2)*, *multcomp(v.1.4-29)*, *TH.data(v.1.1-5)*, *mvtnorm(v.1.3-3)*, *reReg(v.1.4.7)*, *frailtypack(v.3.8.0)*, *survC1(v.1.0-3)*, *MASS(v.7.3-65)*, *doBy(v.4.7.1)*, *boot(v.1.3-31)*, *WINS(v.1.5.1)*, *survival(v.3.8-3)*, *lubridate(v.1.9.4)*, *forcats(v.1.0.1)*, *stringr(v.1.6.0)*, *dplyr(v.1.1.4)*, *purrr(v.1.2.2)*, *readr(v.2.1.5)*, *tidyr(v.1.3.1)*, *tibble(v.3.3.0)*, *ggplot2(v.4.0.1)* and *tidyverse(v.2.0.0)*

**loaded via a namespace (and not attached):** *RColorBrewer(v.1.1-3)*, *rstudioapi(v.0.18.0)*, *magrittr(v.2.0.3)*, *ggstance(v.0.3.7)*, *estimability(v.1.5.1)*, *farver(v.2.1.2)*, *nloptr(v.2.2.1)*, *rmarkdown(v.2.31)*, *ragg(v.1.4.0)*, *vctrs(v.0.6.5)*, *splines2(v.0.5.4)*, *SQUAREM(v.2026.1)*, *rstatix(v.0.7.3)*, *tinytex(v.0.59)*, *htmltools(v.0.5.8.1)*, *haven(v.2.5.5)*, *broom(v.1.0.12)*, *deSolve(v.1.42)*, *Formula(v.1.2-5)*, *parallelly(v.1.47.0)*, *pracma(v.2.4.6)*, *plyr(v.1.8.9)*, *sandwich(v.3.1-1)*, *rootSolve(v.1.8.2.4)*, *zoo(v.1.8-14)*, *lifecycle(v.1.0.5)*, *iterators(v.1.0.14)*, *pkgconfig(v.2.0.3)*, *sjlabelled(v.1.2.0)*, *optimx(v.2025-4.9)*, *Matrix(v.1.7-1)*, *R6(v.2.6.1)*, *fastmap(v.1.2.0)*, *future(v.1.70.0)*, *digest(v.0.6.37)*, *numDeriv(v.2016.8-1.1)*, *colorspace(v.2.1-2)*, *textshaping(v.1.0.1)*, *dfoptim(v.2023.1.0)*, *labeling(v.0.4.3)*, *reda(v.0.5.6)*, *timechange(v.0.3.0)*, *abind(v.1.4-8)*, *mgcv(v.1.9-1)*, *compiler(v.4.4.2)*, *microbenchmark(v.1.5.0)*, *pander(v.0.6.6)*, *withr(v.3.0.2)*, *doParallel(v.1.0.17)*, *S7(v.0.2.0)*, *backports(v.1.5.0)*, *carData(v.3.0-5)*, *ggsignif(v.0.6.4)*, *BB(v.2026.1.0)*, *lava(v.1.9.0)*, *tools(v.4.4.2)*, *otel(v.0.2.0)*, *mstate(v.0.3.3)*, *future.apply(v.1.20.2)*, *glue(v.1.8.0)*, *quadprog(v.1.5-8)*, *nlme(v.3.1-166)*, *grid(v.4.4.2)*, *generics(v.0.1.4)*, *gtable(v.0.3.6)*, *tzdb(v.0.5.0)*, *data.table(v.1.18.2.1)*, *hms(v.1.1.4)*, *utf8(v.1.2.6)*, *car(v.3.1-3)*, *Deriv(v.4.2.0)*, *foreach(v.1.5.2)*, *pillar(v.1.11.1)*, *splines(v.4.4.2)*, *lattice(v.0.22-7)*, *SparseM(v.1.84-2)*, *directlabels(v.2026.4.23)*, *tidyselect(v.1.2.1)*, *muhaz(v.1.2.6.4)*, *knitr(v.1.51)*, *gridExtra(v.2.3)*, *urca(v.1.3-4)*, *forecast(v.9.0.2)*, *stats4(v.4.4.2)*, *xfun(v.0.57)*, *statmod(v.1.5.1)*, *timeDate(v.4052.112)*, *scam(v.1.2-22)*, *stringi(v.1.8.7)*, *lazyeval(v.0.2.2)*, *yaml(v.2.3.10)*, *evaluate(v.1.0.5)*, *codetools(v.0.2-20)*, *cli(v.3.6.5)*, *systemfonts(v.1.3.1)*, *xtable(v.1.8-8)*, *modelr(v.0.1.11)*, *globals(v.0.19.1)*, *coda(v.0.19-4.1)*, *marqLevAlg(v.2.0.8)*, *parallel(v.4.4.2)*, *MatrixModels(v.0.5-4)*, *fracdiff(v.1.5-3)*, *ggforestplot(v.0.1.0)*, *listenv(v.0.10.1)*, *matrixcalc(v.1.0-6)*, *scales(v.1.4.0)*, *prodlim(v.2026.03.11)*, *insight(v.1.5.0)*, *nleqslv(v.3.3.7)* and *rlang(v.1.2.0)*
